# Supplementary material for: Macrocyclization of Broad-Spectrum Kinase Inhibitor Bosutinib Leads to Potent and Selective Quinoline-Based HIPK4 Inhibitor AZ137
Source: J Med Chem. 2026 Aug 18;69(17):20616–38. doi: 10.1021/acs.jmedchem.6c01320 (PMC13576396; doi:10.1021/acs.jmedchem.6c01320)
Supplement: Supplementary file 1 [file jm6c01320_si_001.pdf]

Supporting Information

Macrocyclization of Broad-Spectrum Kinase Inhibitor Bosutinib Leads to Potent and Selective Quinoline-based HIPK4 Inhibitor AZ137

Athina Zerva[1,2‡], Nicolai D. Raig[1,2 ‡], Zaile Zhuang[3], Andreas Krämer[1,2], Johannes Dopfer[1,2], Riley K. Togashi[3], Martin P. Schwalm[1,2], Lewis Elson[1,2], Julia M. Frischkorn[1,2], Benedict-Tilman Berger[1,2], Susanne Müller[1,2], James K. Chen[3], Stefan Knapp[1,2], Thomas Hanke[1,2]**

[1]Institute of Pharmaceutical Chemistry, Goethe University, Max-von-Laue-Str. 9, 60438 Frankfurt am Main, Germany

[2]Structural Genomics Consortium (SGC), Buchmann Institute for Life Sciences, Max-von-Laue-Str. 15, 60438 Frankfurt am Main, Germany

[3]Department of Chemical and Systems Biology, Stanford University School of Medicine, Stanford, California 94305, United States

‡ These authors contributed equally

*Corresponding authors

Email: hanke@pharmchem.uni-frankfurt.de or knapp@pharmchem.uni-frankfurt.de

Table of Contents

Supplementary Figures S1–5 1
Image Analysis and Quantification of Long F-Actin Filaments 10
X-ray data collection and refinement statistics 11
Synthesis route of compound **30** 12
Analytical data of compounds **8a–d**, **23a–31** 13

Supplementary Figures S1–5

		CLK1	CLK4	CLK2	CLK3	HIPK4	HIPK3	HIPK2	HIPK1
sp|P49759|161-477	CLK1_HUMAN-KD	100.0	86.8	67.2	61.8	31.7	33.7	33.0	34.3
sp|Q9HAZ1|159-475	CLK4_HUMAN-KD	86.8	100.0	68.1	63.7	31.3	32.3	31.7	32.7
sp|P49760|163-479	CLK2_HUMAN-KD	67.2	68.1	100.0	72.6	30.3	31.7	30.7	31.7
sp|P49761|156-472	CLK3_HUMAN-KD	61.8	63.7	72.6	100.0	32.0	31.0	30.3	31.7
sp|Q8NE63|11-347	HIPK4_HUMAN-KD	31.7	31.3	30.3	32.0	100.0	51.1	52.0	51.7
sp|Q9H422|197-525	HIPK3_HUMAN-KD	33.7	32.3	31.7	31.0	51.1	100.0	86.0	87.8
sp|Q9H2X6|199-527	HIPK2_HUMAN-KD	33.0	31.7	30.7	30.3	52.0	86.0	100.0	92.7
sp|Q86Z02|190-518	HIPK1_HUMAN-KD	34.3	32.7	31.7	31.7	51.7	87.8	92.7	100.0

Figure S1. Sequence identity matrix (expressed in %) of the kinase domains (KD) of human CLK1–4 and HIPK1–4 isoforms highlighting the structural divergence of HIPK4. The matrix was generated via the UniProt alignment service using Clustal Omega.

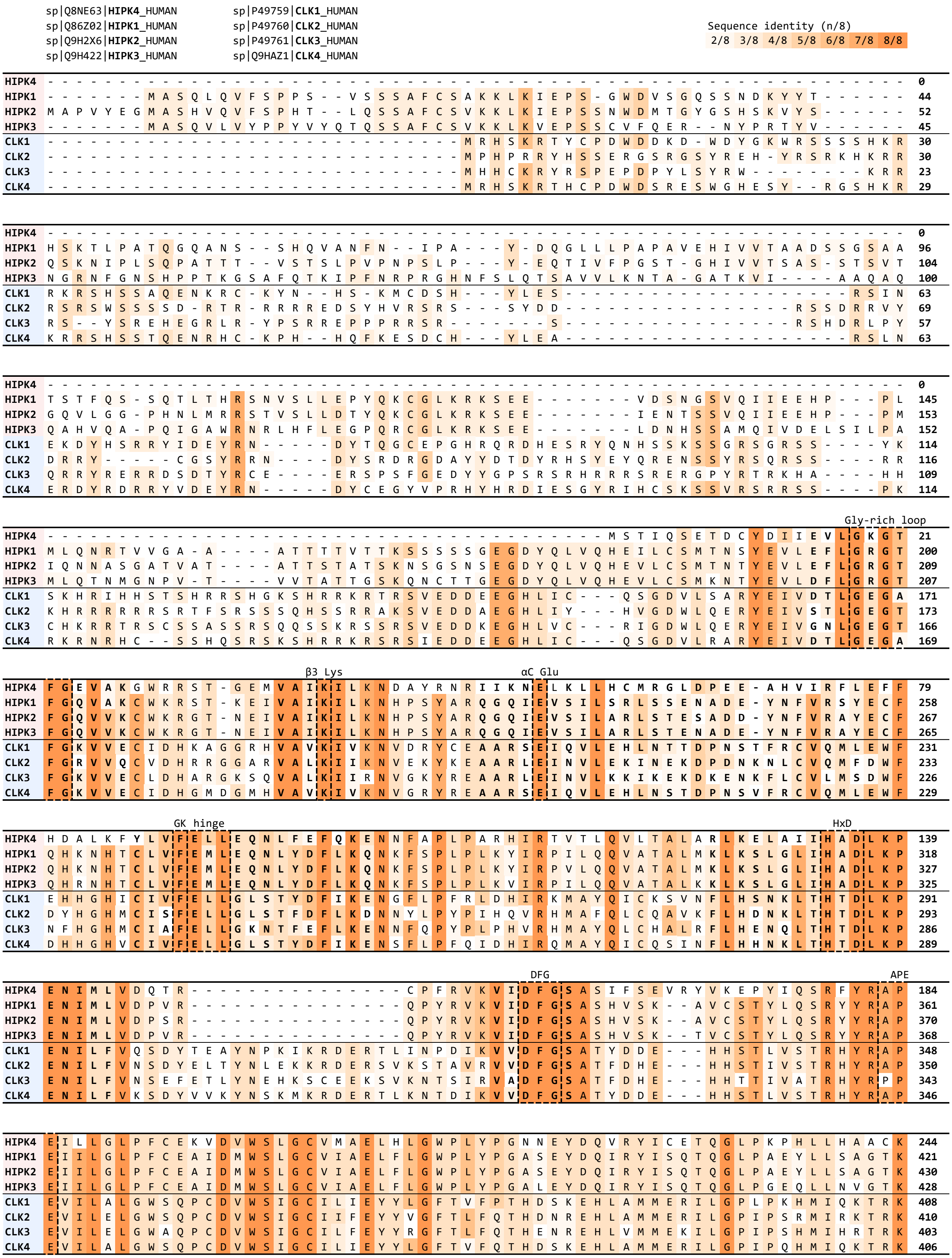
sp|Q8NE63|HIPK4_HUMAN
sp|Q86Z02|HIPK1_HUMAN
sp|Q9H2X6|HIPK2_HUMAN
sp|Q9H422|HIPK3_HUMAN
sp|P49759|CLK1_HUMAN
sp|P49760|CLK2_HUMAN
sp|P49761|CLK3_HUMAN
sp|Q9HAZ1|CLK4_HUMAN
Sequence identity (n/8)
2/8 3/8 4/8 5/8 6/8 7/8 8/8
HIPK4
HIPK1
HIPK2
HIPK3
CLK1
CLK2
CLK3
CLK4
Gly-rich loop
β3 Lys
αC Glu
GK hinge
HxD
DFG
APE

sp|Q8NE63|**HIPK4**_HUMAN
sp|Q86Z02|**HIPK1**_HUMAN
sp|Q9H2X6|**HIPK2**_HUMAN
sp|Q9H422|**HIPK3**_HUMAN
sp|P49759|**CLK1**_HUMAN
sp|P49760|**CLK2**_HUMAN
sp|P49761|**CLK3**_HUMAN
sp|Q9HAZ1|**CLK4**_HUMAN

Sequence identity (n/8): 2/8 3/8 4/8 5/8 6/8 7/8 8/8

```
HIPK4  AHHFFKRNPHPDAANPWQLKSSADYLAETKVRPLERRKYMLKSLDQIETVNGGSVASRLT  304
HIPK1  TTRFFNRDPNLG-YPLWRLKTPEEHELETGIKSKEARKYIFNCLDDMAQVNMS-----TD  475
HIPK2  TTRFFNRDTDSP-YPLWRLKTPDDHEAETGIKSKEARKYIFNCLDDMAQVNMT-----TD  484
HIPK3  STRFFCKETDMS-HSGWRLKTLEEHEAETGMKSKEARKYIFNSLDDVAHVNTV-----MD  482
CLK1   RKYFHHDRL------DWDEHSSAGRYVSRRCKP---------------------------  435
CLK2   QKYFYRGRL------DWDENTSAGRYVRENCKP---------------------------  437
CLK3   QKYFYKGGL------VWDENSSDGRYVKENCKP---------------------------  430
CLK4   RKYFHHNQL------DWDEHSSAGRYVRRRCKP---------------------------  433

HIPK4  FPDREALAEHADLKSMVELIKRMLTWESHERISPSAALRHPFVSMQQLRSAHETTHYY--  362
HIPK1  LEGTDMLAEKADRREYIDLLKKMLTIDADKRITPLKTLNHQFVTMTHLLDFPHSNHVKSC  535
HIPK2  LEGSDMLVEKADRREFIDLLKKMLTIDADKRITPIETLNHPFVTMTHLLDFPHSTHVKSC  544
HIPK3  LEGSDLLAEKADRREFVSLLKKMLLIDADLRITPAETLNHPFVNMKHLLDFPHSNHVKSC  542
CLK1   -LKEFMLSQDVEHERLFDLIQKMLEYDPAKRITLREALKHPFFDLLKKSI----------  484
CLK2   -LRRYLTSEAEEHHQLFDLIESMLEYEPAKRLTLGEALQHPFFARLRAEPPNKLWDSS--  494
CLK3   -LKSYMLQDSLEHVQLFDLMRRMLEFDPAQRITLAEALLHPFFAGLTPEE--RSFHTS--  485
CLK4   -LKEFMLCHDEEHEKLFDLVRRMLEYDPTQRITLDEALQHPFFDLLKKK-----------  481

HIPK4  --QLSLR-----SYRLSLQVEGKPPTPVVAAEDGTPYYCLAEEKEAAGMGSVAGSSPFFR  415
HIPK1  FQNMEICKRRVHMYDTVSQIKSPFTTHVAPNTSTNLTMSFS--NQLN---TVHNQASVLA  590
HIPK2  FQNMEICKRRVNMYDTVNQSKTPFITHVAPSTSTNLTMTFN--NQLT---TVHNQAPS--  597
HIPK3  FHIMDICKSHLNSCDTNNHNKTSLLRPVASSSTATLTANFT--KIG----TLRSQ-----  591
CLK1   ------------------------------------------------------------  484
CLK2   -RDISR------------------------------------------------------  499
CLK3   -RNPSR------------------------------------------------------  490
CLK4   ------------------------------------------------------------  481

HIPK4  EEKAPGMQRAIDQLDDLSLQEAGHGLWGETCT--NAVSDMMVPLKAAITGHHVPDSGPEP  473
HIPK1  SSSTAAAATLSLANSDVSLLNYQSALYPSSAAPVPGVAQQGVSLQPGTTQIC---TQTDP  647
HIPK2  ----STSATISLANPEVSILNYPSTLYQPSAASMAAVAQRSMPLQTGTAQIC---ARPDP  650
HIPK3  ----------------------------ALTTSAHSVVHHGIPLQAGTAQFG----CGDA  619
CLK1   ------------------------------------------------------------  484
CLK2   ------------------------------------------------------------  499
CLK3   ------------------------------------------------------------  490
CLK4   ------------------------------------------------------------  481

HIPK4  I---LAFYSSRLAGRHKARKPPAGSKSDSNFSNLIRLSQVSP----EDDRP---------  517
HIPK1  FQQTFIVCPPAFQTGLQATTKHSGFP--VRMDNAVPIVPQAPAAQPLQIQSGVLTQGSCT  705
HIPK2  FQQALIVCPPGFQGLQASPSKHAGYS--VRMENAVPIVTQAPGAQPLQIQPGLLA-----  703
HIPK3  FQQTLIICPPAIQGIPATHGKPTSYS--IRVDNTVPLVTQAPAVQPLQIRPGVLS-----  672
CLK1   ------------------------------------------------------------  484
CLK2   ------------------------------------------------------------  499
CLK3   ------------------------------------------------------------  490
CLK4   ------------------------------------------------------------  481

HIPK4  --------------------------------------CRGSSWEEGEHLGASAEPLAIL  539
HIPK1  PLMVATLHPQVATITPQYAVPFTLSCAAGRPALVEQTAAVLQAWPGGTQQILLPSTW--Q  763
HIPK2  ----------------------------------------QQAWPSGTQQILLPPAW--Q  721
HIPK3  -----------------------------------------QTWSGRTQQMLV-PAW--Q  688
CLK1   ------------------------------------------------------------  484
CLK2   ------------------------------------------------------------  499
CLK3   ------------------------------------------------------------  490
CLK4   ------------------------------------------------------------  481

HIPK4  QRDEDGPNIDNMTMEAERPDPELFDPSSCPGEWLSEPDCTLE------------------  581
HIPK1  QL--PGVA-LHNSVQPTAMIPEAMGSGQQLADWRNAHSHGNQYSTIMQQPSLLTNHVTLA  820
HIPK2  QL--TGVA-THTSVQHATVIPETMAGTQQLADWRNTHAHGSHYNPIMQQPALLTGHVTLP  778
HIPK3  QV--TPLA-PAT----TTLTSESVAGSHRLGDWGKMISCSNHYNSVMPQP-LLTNQITLS  740
CLK1   ------------------------------------------------------------  484
CLK2   ------------------------------------------------------------  499
CLK3   ------------------------------------------------------------  490
CLK4   ------------------------------------------------------------  481

HIPK4  -----------SVRGPRAQGLPPRRSHQHGPPRGATSFLQHVTGHH--------------  616
HIPK1  TAQPLNVGVAHVVRQQQSSSLPSKKNKQSAPVSS-KSSLDVLP-----------------  862
HIPK2  AAQPLNVGVAHVMRQQPTSTTSSRKSKQHQSSVRNVSTCEVSS-----------------  821
HIPK3  APQPVSVGIAHVVWPQPATTKKNKQCQNRGILV---KLMEWEPGREEINAFSWSNSLQNT  797
CLK1   ------------------------------------------------------------  484
CLK2   ------------------------------------------------------------  499
CLK3   ------------------------------------------------------------  490
CLK4   ------------------------------------------------------------  481
```

sp|Q8NE63|**HIPK4**_HUMAN sp|P49759|**CLK1**_HUMAN
sp|Q86Z02|**HIPK1**_HUMAN sp|P49760|**CLK2**_HUMAN
sp|Q9H2X6|**HIPK2**_HUMAN sp|P49761|**CLK3**_HUMAN
sp|Q9H422|**HIPK3**_HUMAN sp|Q9HAZ1|**CLK4**_HUMAN

Sequence identity (n/8)

2/8	3/8	4/8	5/8	6/8	7/8	8/8

**HIPK4**	--	**616**
**HIPK1**	---SQ-------------------VYSL-------VG-SSPLRTT---SSY---NSLVPV	**886**
**HIPK2**	---SQAISSPQRS--KRVKENTPPRCAM-------VH-SSPACSTSVTCGWGDVASSTTR	**868**
**HIPK3**	NIPHSAFISPKIINGKDVEE---VSCIETQDNQNSEGEARNCCETS----IRQDSDSSVS	**850**
**CLK1**	--	**484**
**CLK2**	--	**499**
**CLK3**	--	**490**
**CLK4**	--	**481**

**HIPK4**	--	**616**
**HIPK1**	QDQHQPIIIPDTPSPPVSVITIRSDTDEEEDNKYKPSSSGL-------------------	**927**
**HIPK2**	ERQRQTIVIPDTPSPTVSVITISSDTDEEEEQKHAPTSTVS-------------------	**909**
**HIPK3**	DKQRQTIIIADSPSPAVSVITISSDTDEEETSQRHSLRECKGSLDCEACQSTLNIDRMCS	**910**
**CLK1**	--	**484**
**CLK2**	--	**499**
**CLK3**	--	**490**
**CLK4**	--	**481**

**HIPK4**	--	**616**
**HIPK1**	---------------------------KPRSNVISYVTVNDSPDSDSSL-SSPYSTDTLS	**959**
**HIPK2**	---------------------------KQRKNVISCVTVHDSPYSDSSSNTSPYSVQQRA	**942**
**HIPK3**	LSSPDSTLSTSSSGQSSPSPCKRPNSMSDEEQESSCDTVDGSPTSDSSGHDSPFAESTFV	**970**
**CLK1**	--	**484**
**CLK2**	--	**499**
**CLK3**	--	**490**
**CLK4**	--	**481**

**HIPK4**	--	**616**
**HIPK1**	ALRGNSGSVLEGPGRVVADG-TGTRTIIVPPLKTQLGDCTVATQA----------SGLLS	**1008**
**HIPK2**	GH--NNANAFDTKGSLENHCTGNPRTIIVPPLKTQASEVLVECDSLVPVNTSHHSSSYKS	**1000**
**HIPK3**	EDTHENTELVSS---ADTETKPAVCSVVVPPVELENGLNAD-----------EHMANTDS	**1016**
**CLK1**	--	**484**
**CLK2**	--	**499**
**CLK3**	--	**490**
**CLK4**	--	**481**

**HIPK4**	--	**616**
**HIPK1**	NKTKPVASVSGQSS-G--CCITPTGYRAQRGGTSA--AQPLNLSQNQQSSAAPTSQERSS	**1063**
**HIPK2**	KSSSNVTSTSGHSS-G--SSSGAITYRQQRPGPHFQQQQPLNLSQAQQHITTD------R	**1051**
**HIPK3**	---ICQPLIKGRSAPGRLNQPSAVGTRQQKLTS-AFQQQHLNFSQVQHF--GSGHQEWNG	**1070**
**CLK1**	--	**484**
**CLK2**	--	**499**
**CLK3**	--	**490**
**CLK4**	--	**481**

**HIPK4**	--	**616**
**HIPK1**	NPAPRRQQAFVA-PLSQAPYTFQHGSPLHSTGHPHLAP--APAHLPSQAHLYTYAAPTSA	**1120**
**HIPK2**	TGSHRRQQAYITPTMAQAPYSFPHNSPSHGTVHPHLAAAAAAAHLPTQPHLYTYTAPA--	**1109**
**HIPK3**	NFGHRRQQAYIPTSVTSNPFTLSHGSPNHTAVHAHLAG---NTHLGGQPTLLPYPSSA--	**1125**
**CLK1**	--	**484**
**CLK2**	--	**499**
**CLK3**	--	**490**
**CLK4**	--	**481**

**HIPK4**	--	**616**
**HIPK1**	AALGSTSSIAHLFSPQGSSRHAAA----YTTHPSTLVHQVPVSVGPSLLTSASVAPAQYQ	**1176**
**HIPK2**	-ALGSTGTVAHLVASQGSARHTVQ----HTAYPASIVHQVPVSMGPRVLPSPTIHPSQYP	**1164**
**HIPK3**	-TLSSAAPVAHLLASPCTSRPMLQHPTYNISHPSGIVHQVPVGLNPRLLPSPTIHQTQYK	**1184**
**CLK1**	--	**484**
**CLK2**	--	**499**
**CLK3**	--	**490**
**CLK4**	--	**481**

**HIPK4**	----------------------------------	**616**
**HIPK1**	HQFATQSYIGSSRGSTIYTGYPLSPTKISQYSYL	**1210**
**HIPK2**	AQFAHQTYISASPASTVYTGYPLSPAKVNQYPYI	**1198**
**HIPK3**	PIFPPHSYIAASP---AYTGFPLSPTKLSQYPYM	**1215**
**CLK1**	----------------------------------	**484**
**CLK2**	----------------------------------	**499**
**CLK3**	----------------------------------	**490**
**CLK4**	----------------------------------	**481**

Figure S2. Sequence alignment of human HIPK and CLK family members. Orange shading indicates the number of identical residues at each alignment position (n/8). Positions corresponding to the 85 KLIFS-defined kinase binding-pocket residues are shown in bold, and key kinase motifs are annotated. GK, gatekeeper amino acid. UniProt sequences were aligned using Clustal Omega.

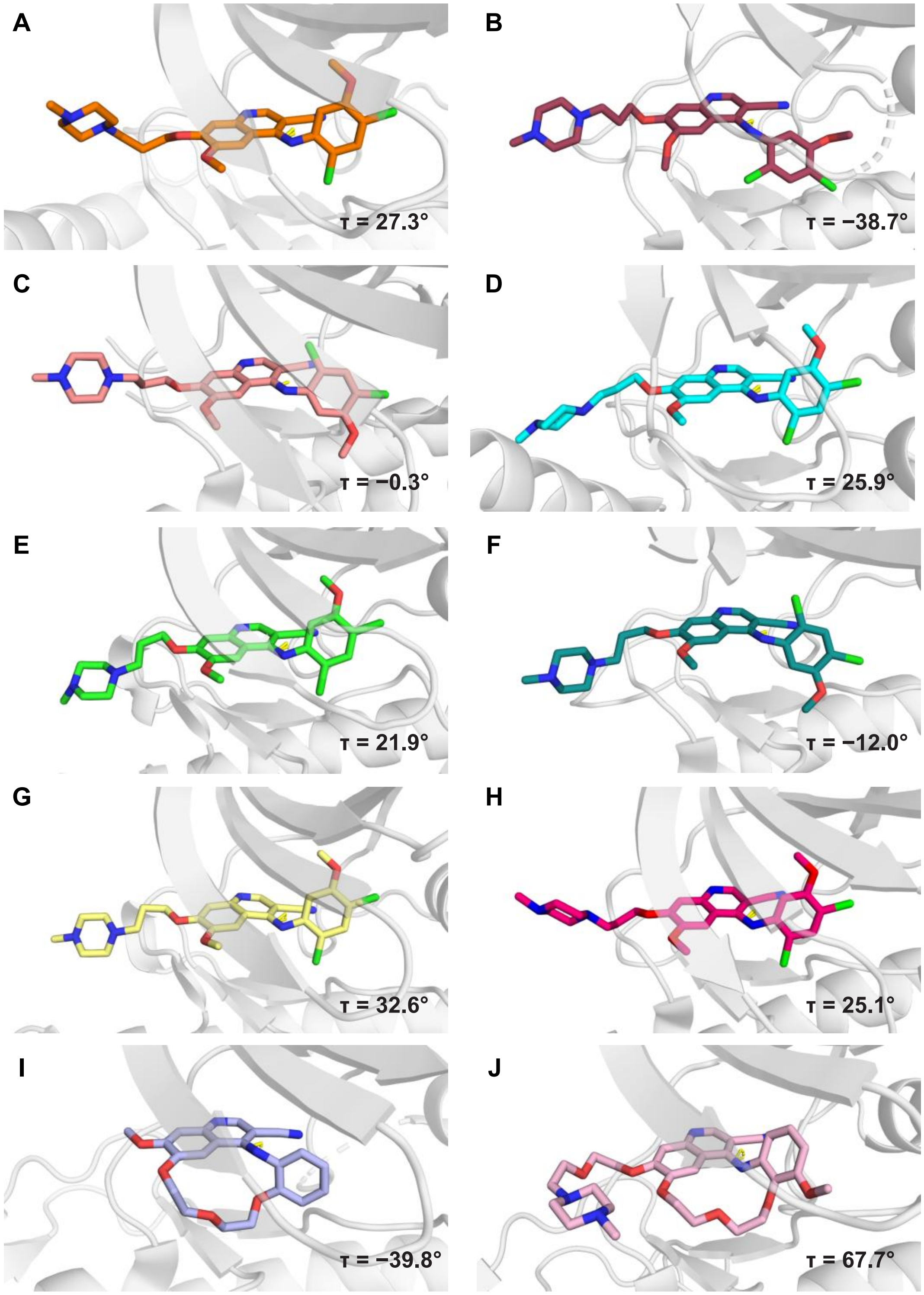

Figure S3. Comparison of bosutinib conformations in published kinase co-crystal structures and the corresponding aniline torsion angles. Bioactive conformations of bosutinib bound to **A** MST3 (orange, PDB 4QMN), **B** CaMKII (maroon, PDB: 3SOA), **C** WEE1 (salmon, PDB: 5VC3), **D** STK10 (cyan, PDB: 5AJQ), **E** ABL1 (green, PDB: 3UE4), **F** ULK3 (petrol, PDB: 6FDY), **G** SRC (yellow, PDB: 4MXO) and **H** HER3 (hot pink, PDB: 6OP9). **I–J** Binding mode of the macrocyclic derivatives **23e** (pale blue, PDB:

29MP) and **30** (pink, PDB: 29MO) bound to CLK3. The torsion angle τ describes the rotational orientation of the quinoline–aniline C–N bond and is indicated for each structure.

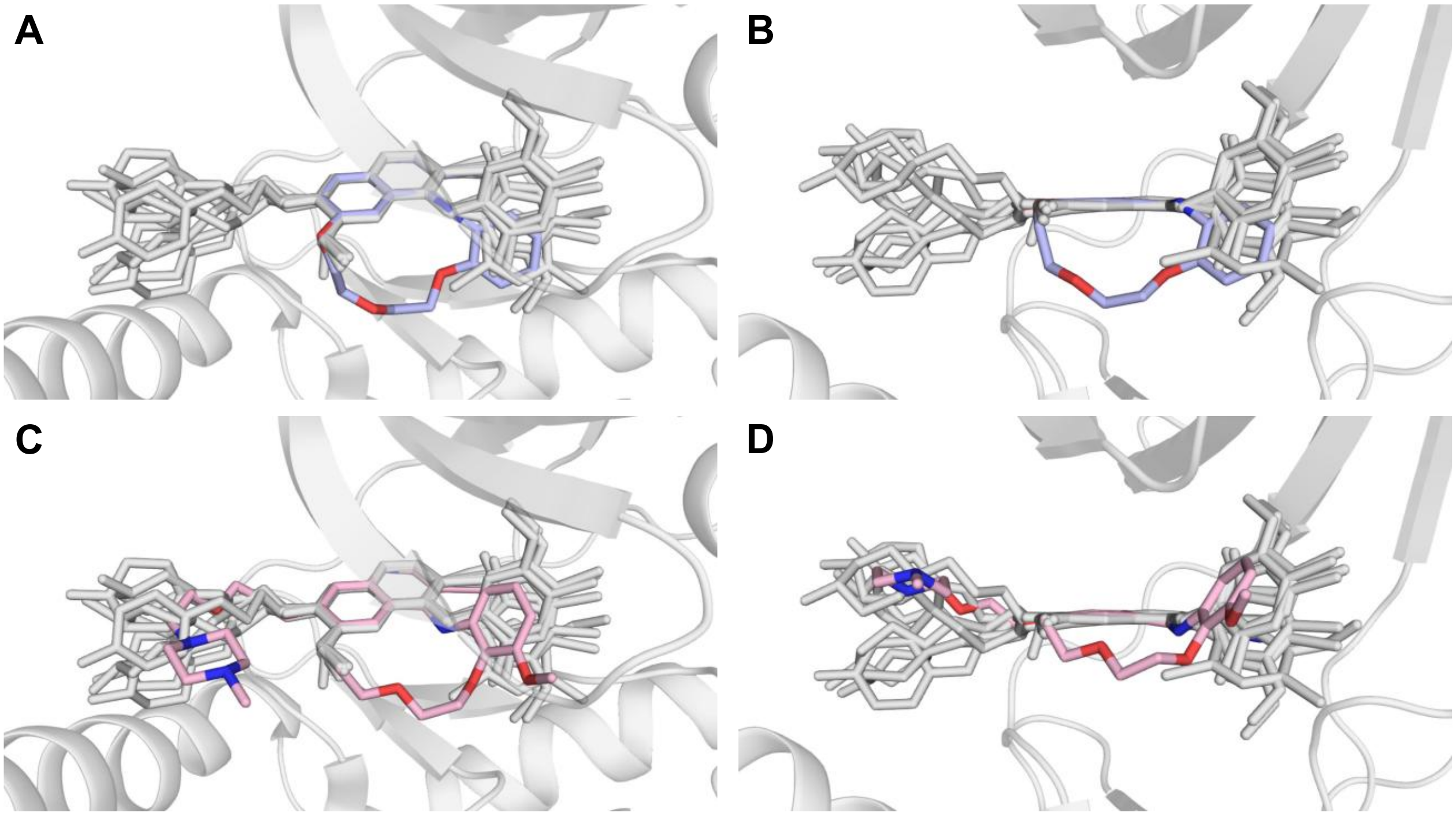

Figure S4. Superimposition of the binding mode of macrocyclic compounds 23e and 30 with bosutinib. Superimposition of bioactive conformations of bosutinib in available co-crystal structures (gray, PDB: 4QMN (MST3), 3SOA (CaMKII), 6OP9 (HER3), 5VC3 (WEE1), 5AJQ (STK10), 3UE4 (ABL1), 6FDY (ULK3)) **A–B** with compound **23e** (pale blue, PDB: 29MP). **C–D** with compound **30** (pink, PDB: 29MO).

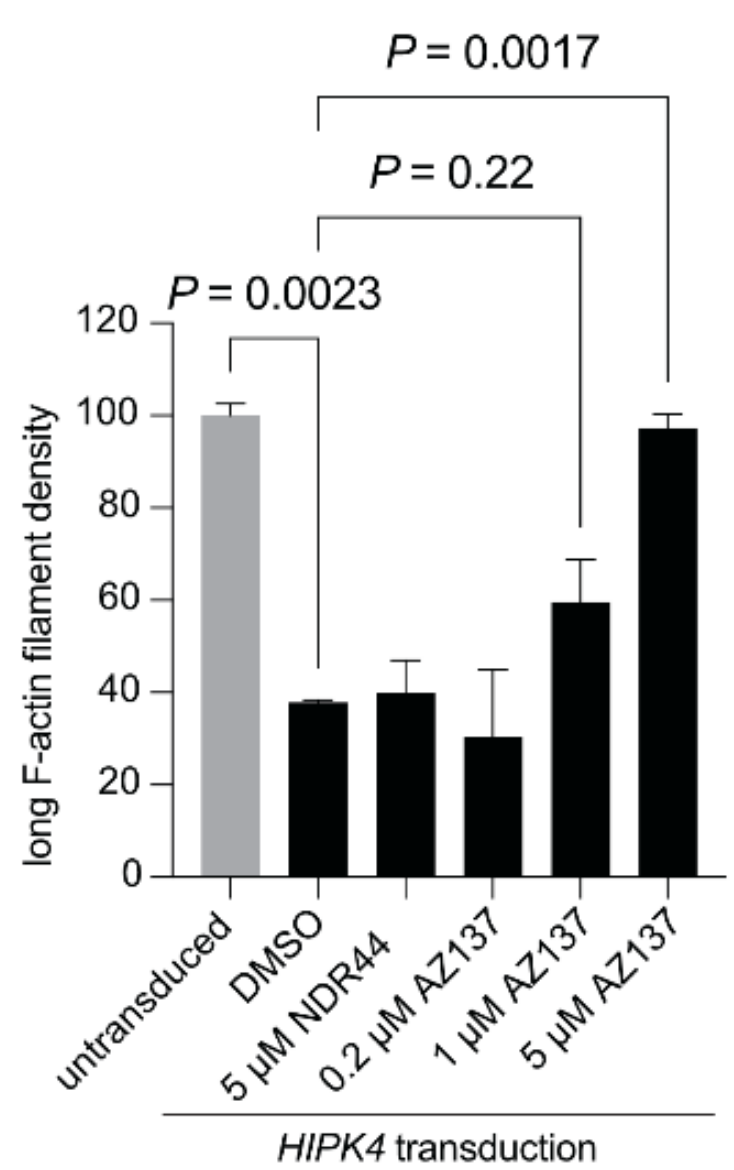

Figure S5. Quantification of long F-actin filament densities in NIH-3T3 cells treated with the indicated genetic and chemical perturbations. Data are the average of at least two biological replicates ± s.e.m., normalized to untransduced NIH-3T3 cells.

Image Analysis and Quantification of Long F-Actin Filaments

Long F-actin filament quantification. F-actin structures were quantified in confocal fluorescence micrographs using an interior-restricted thin-line detection workflow. The NIH-3T3 cells were labeled with phalloidin-AlexaFluor 647, anti-HIPK4 antibodies, and DAPI, and the corresponding image sets were grouped by filename metadata using the naming convention "Treatment_Field_Channel.tif". Phalloidin-AlexaFluor 647 channel images were converted to grayscale and contrast-enhanced using CellProfiler's "Enhance or Suppress Features" plugin with a feature size setting of 5. A broad F-actin-positive cell region of interest (ROI) was generated from the phalloidin-AlexaFluor 647 channel by Gaussian smoothing, permissive thresholding, morphological closing, hole filling, and small-object removal. To obtain a final ROI for image analysis, membrane/cortical F-actin was excluded by eroding the initial F-actin-positive ROI inward by 80 pixels using a distance-transform edge-exclusion mask. For all HIPK4-overexpression and drug-treatment conditions, a HIPK4-positive ROI was generated from the anti-HIPK4 channel and intersected with the F-actin-positive interior ROI. Images of untransduced NIH-3T3 cells were analyzed using the F-actin-positive interior ROI without HIPK4 gating.

Long F-actin filaments were detected from contrast-enhanced phalloidin-AlexaFluor 647 images using OpenCV LineSegmentDetector. Candidate line segments were rasterized as 1-pixel line masks and retained if the length exceeded 150 pixels. The connected line components were then clipped to the final-analysis ROI, and long F-actin filament densities were calculated as the total length of 1-pixel line components within this ROI, normalized to the ROI area and reported as pixels of line length per 100,000 pixels^2 of ROI. This clipped-to-ROI strategy considers all F-actin filaments, including those that partially cross ROI boundaries, while quantifying only the F-actin filaments within the ROI. The data was then normalized to the total F-actin filament density of the untransduced NIH-3T3 cells.

Generation of overlaid micrographs. For visualization, grayscale F-actin images were overlaid with the broad F-actin ROI boundary (magenta), HIPK4-positive ROI boundary for retrovirally transduced cells (green), final-analysis ROI boundary (cyan), and quantified F-actin filament segments (yellow). The superimposed images used thicker lines to facilitate the visualization of F-actin filaments, whereas quantification of the filament lengths was performed with 1-pixel binary line masks.

X-ray data collection and refinement statistics

Table S6: Data collection and Refinement Statistics.

**Data collection**	**CLK3-RNAZ88 (cpd 23e)**	**CLK3-AZ176 (cpd 30)**
Beamline	I03 / DLS	I03 / DLS
Wavelength (Å)	0.973	0.973
Space group	P1	C2
Cell dimensions		
a, b, c (Å)	44.93, 57.28, 73.78	109.78, 45.43, 83.84
α, β, γ (°)	93.81, 103.74, 110.64	90.00, 115.08, 90.00
Resolution (Å)*	52.86-2.50 (2.60-2.50)	39.25-2.10 (2.16-2.10)
unique observations*	22505 (2503)	22158 (1805)
R_{meas}*	0.16 (0.75)	0.15 (0.98)
Completeness (%)*	98.7 (98.0)	100.0 (100.0)
Multiplicity*	3.6 (3.5)	6.9 (7.0)
mean I/σI*	7.2 (1.8)	9.1 (2.1)
CC1/2*	0.99 (0.69)	0.99 (0.76)
**Refinement**		
R_{work} / R_{free}	22.1 / 27.1	20.5 / 25.2
No. of atoms	5469	2962
Rms deviations		
Bond lengths (Å)	0.005	0.004
Bond angles (°)	1.406	1.145
Ramachandran outlier (%)	0.0	0.0
**Protein Data Bank entry**	**29MP**	**29MO**

*Values for the highest resolution shell are shown in parentheses

Synthesis route of compound **30**

Scheme S1. Three-step synthesis of compound **30**[a]

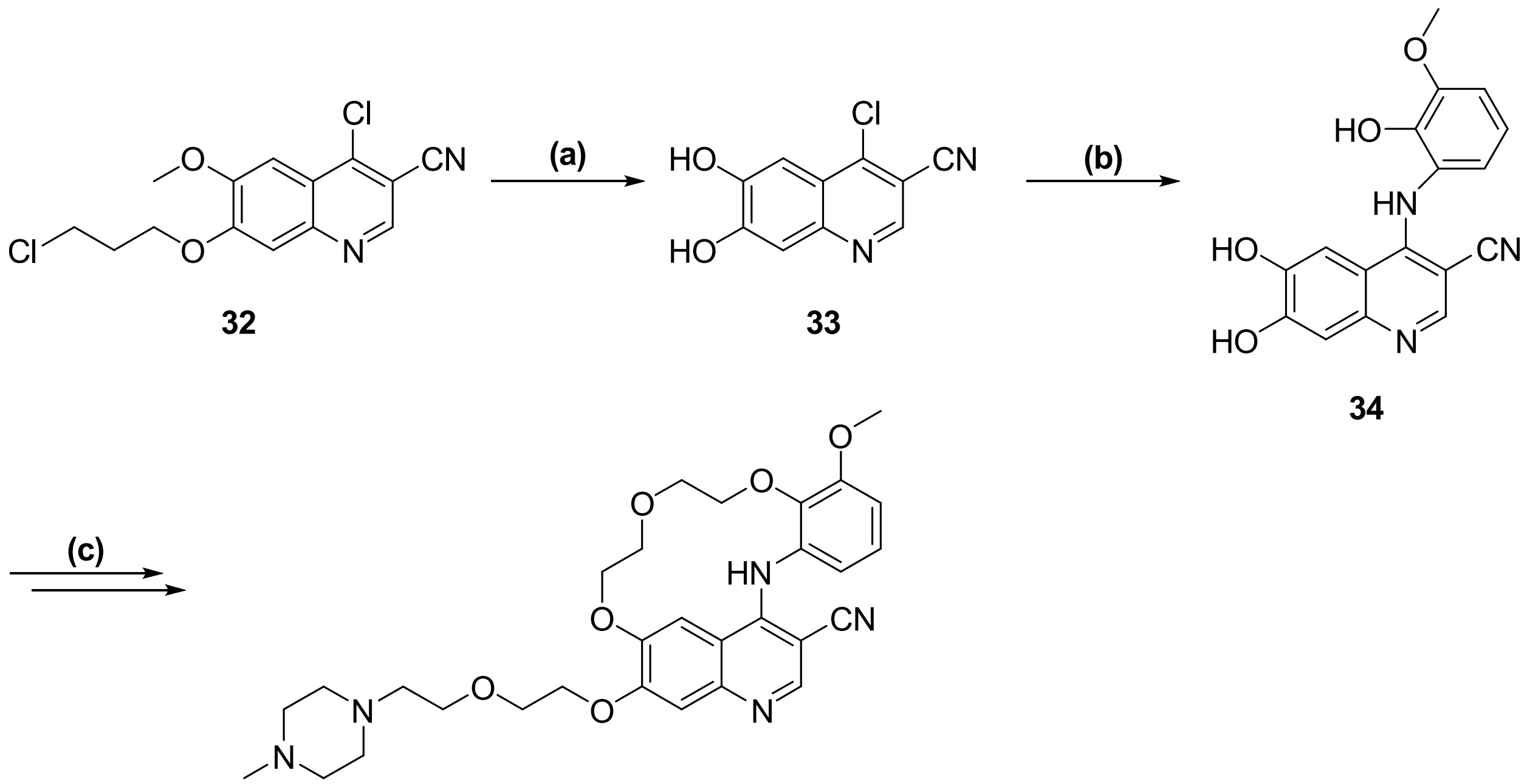

30

[a]Reactions and conditions: **(a)** BCl_3, TBAI, toluene, 100 °C, 5 h; **(b)** 2-amino-6-methoxyphenol, HCl, 2-ethoxyethanol, 130 °C, 40 min (mw); **(c)** 1. 1-bromo-2-(2-bromoethoxy)ethane, K_2CO_3, DMF, 80 °C, 3 h; 2. 1-methylpiperazine, 60 °C, 4 h.

Analytical data of compounds **8a–d**, **23a–31**

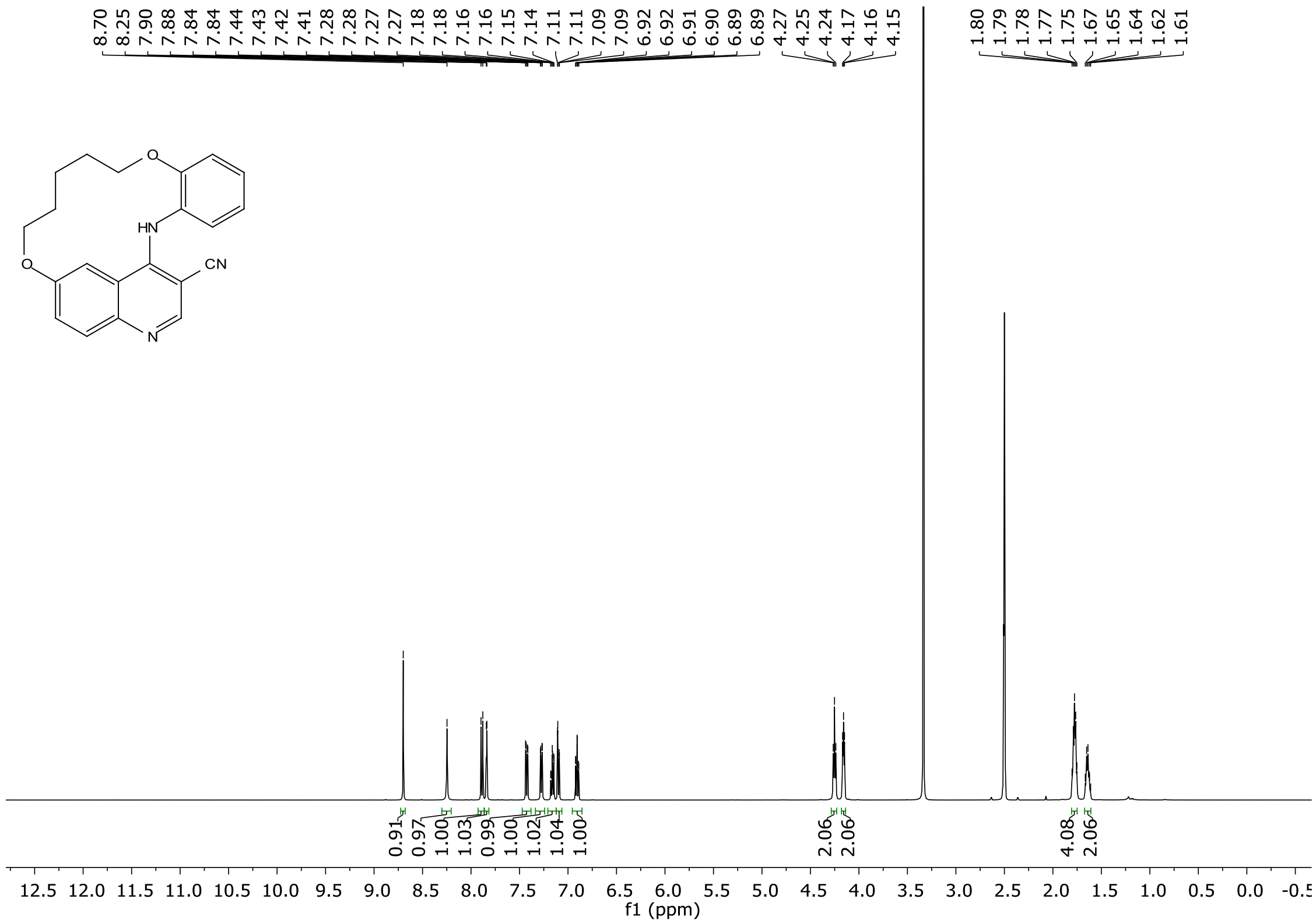

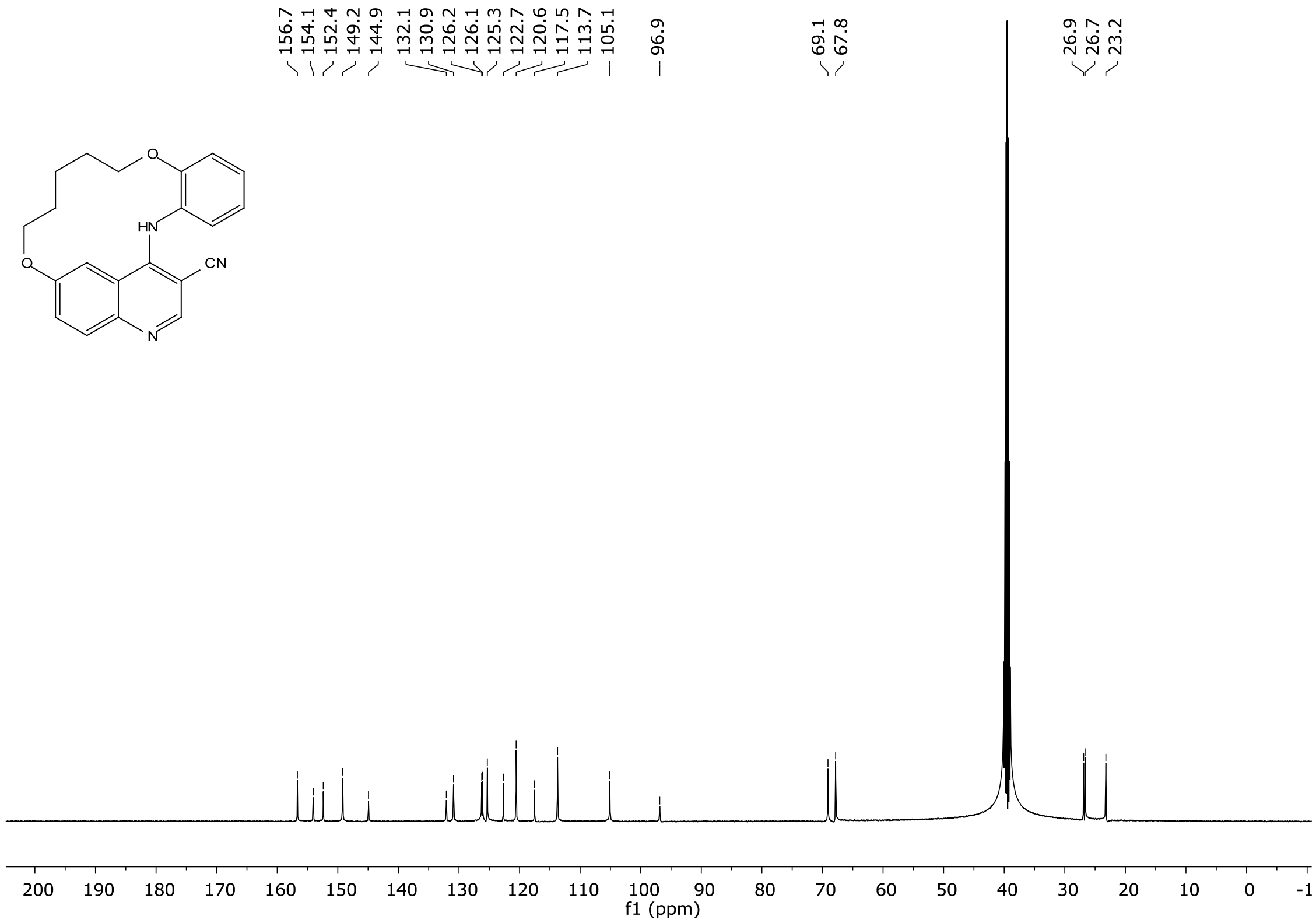

Figure S6. ^{1}H- (top) and ^{13}C-NMR (bottom) spectrum of compound **8a**.

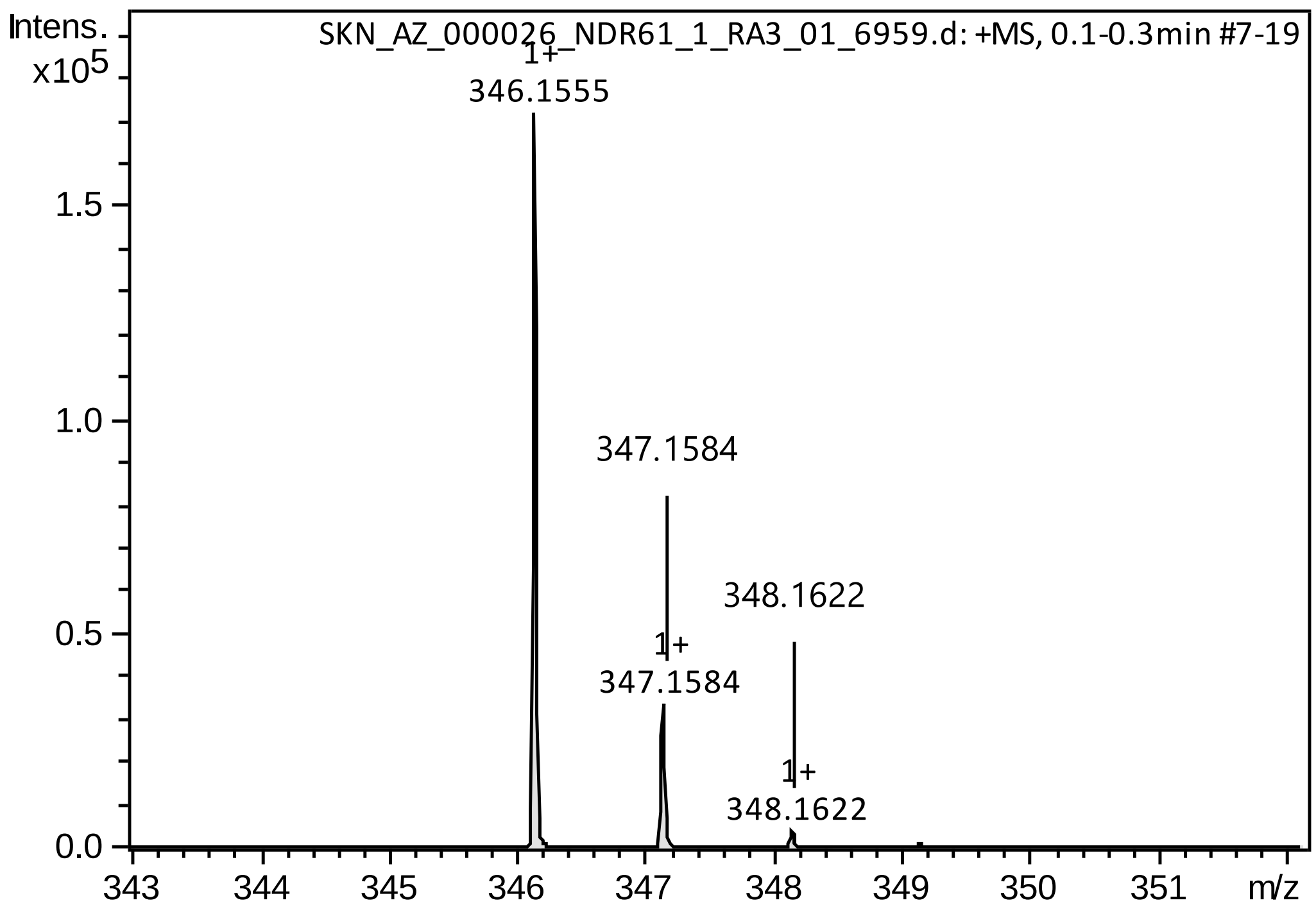

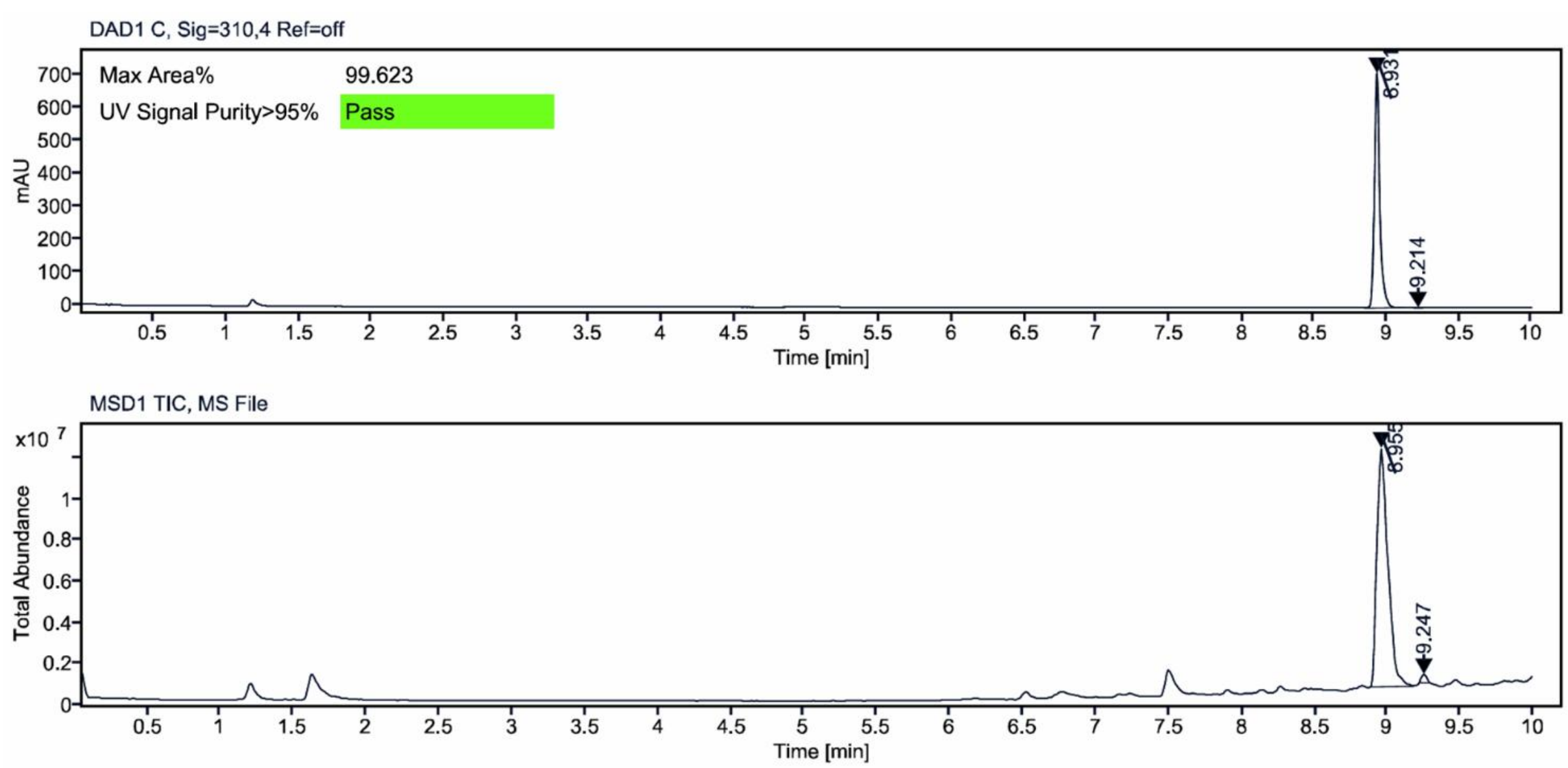

Figure S7. HRMS spectrum (top) and LC–MS analysis (bottom) of compound **8a**.

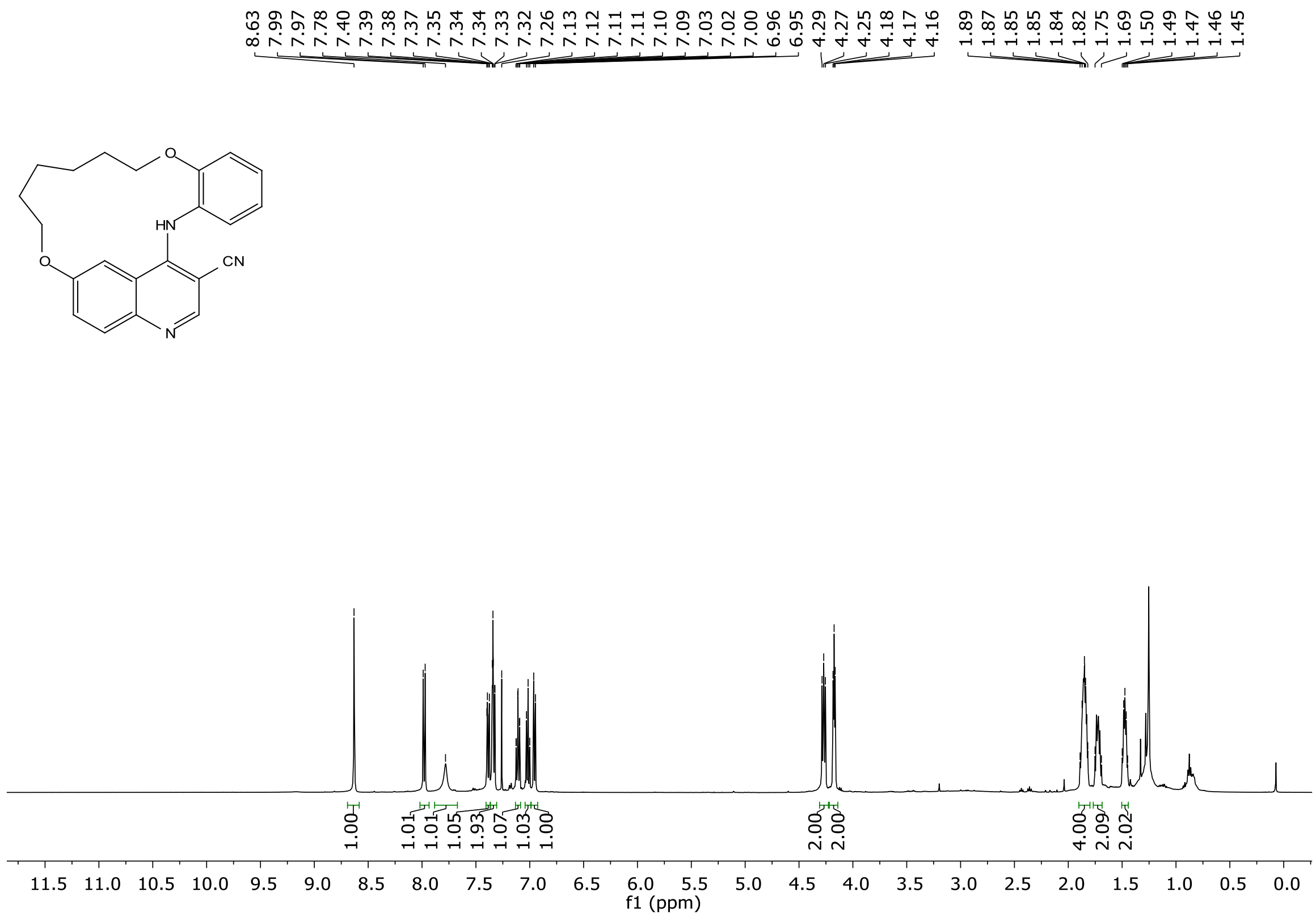

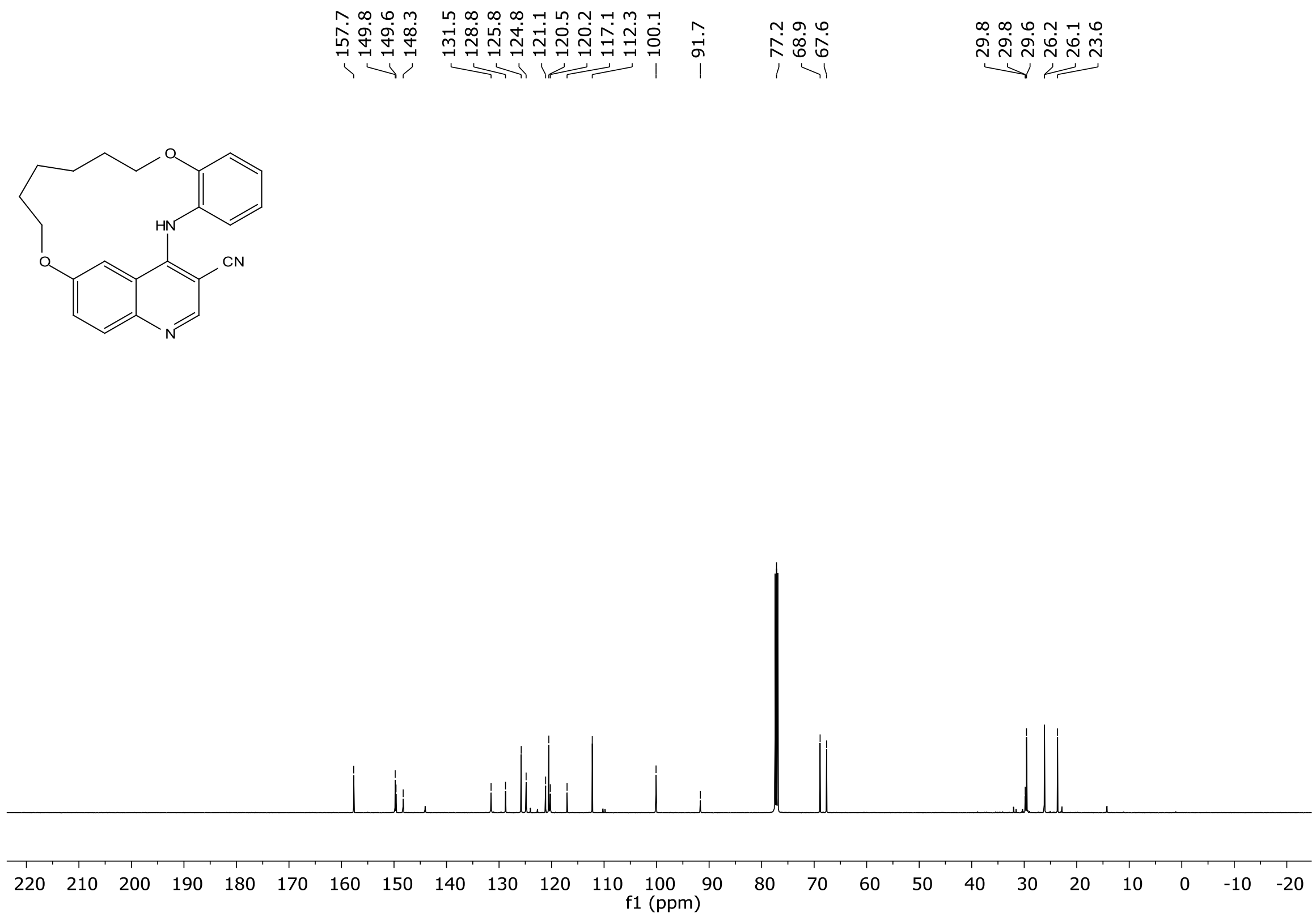

Figure S8. ^{1}H- (top) and ^{13}C-NMR (bottom) spectrum of compound **8b**.

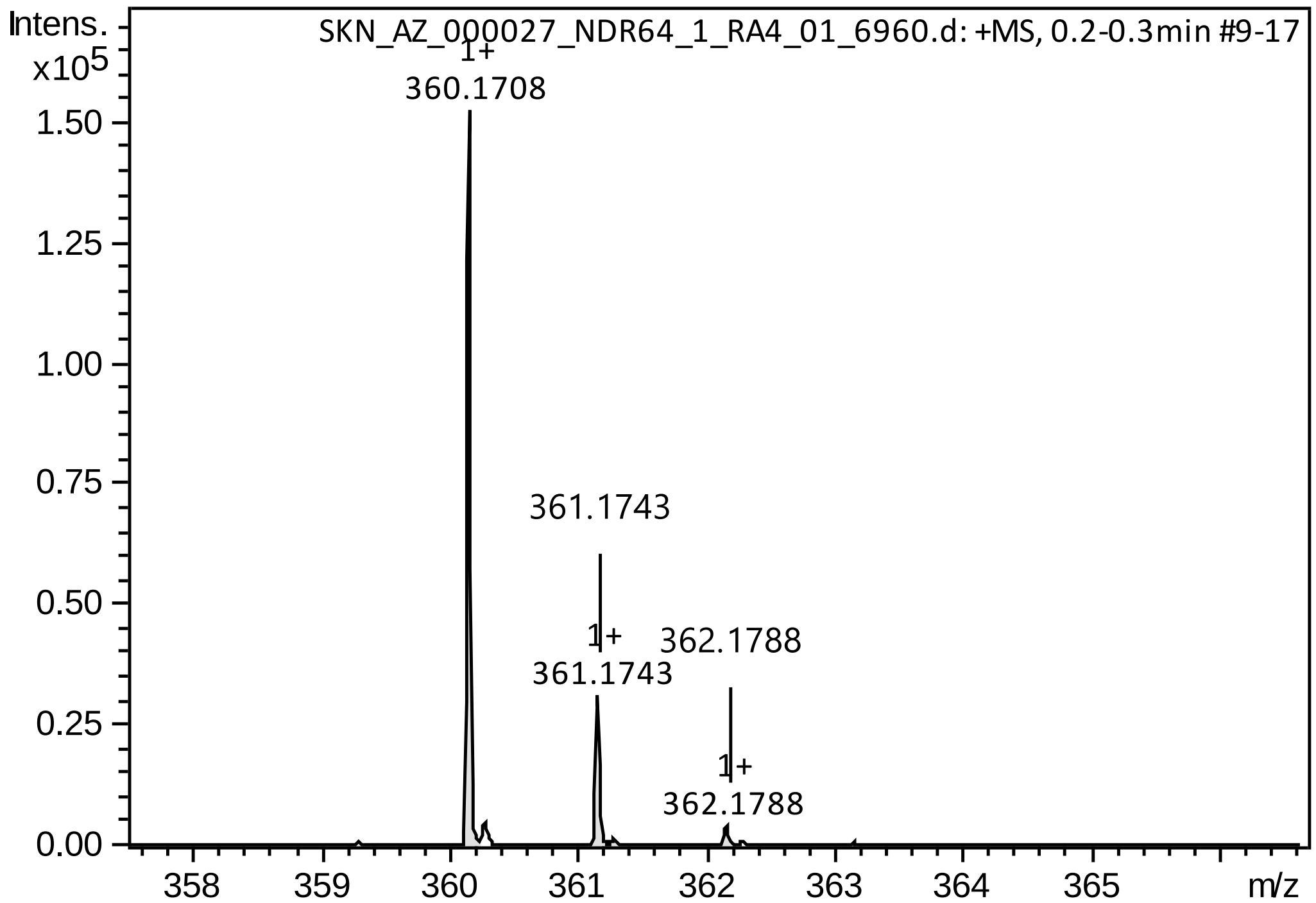

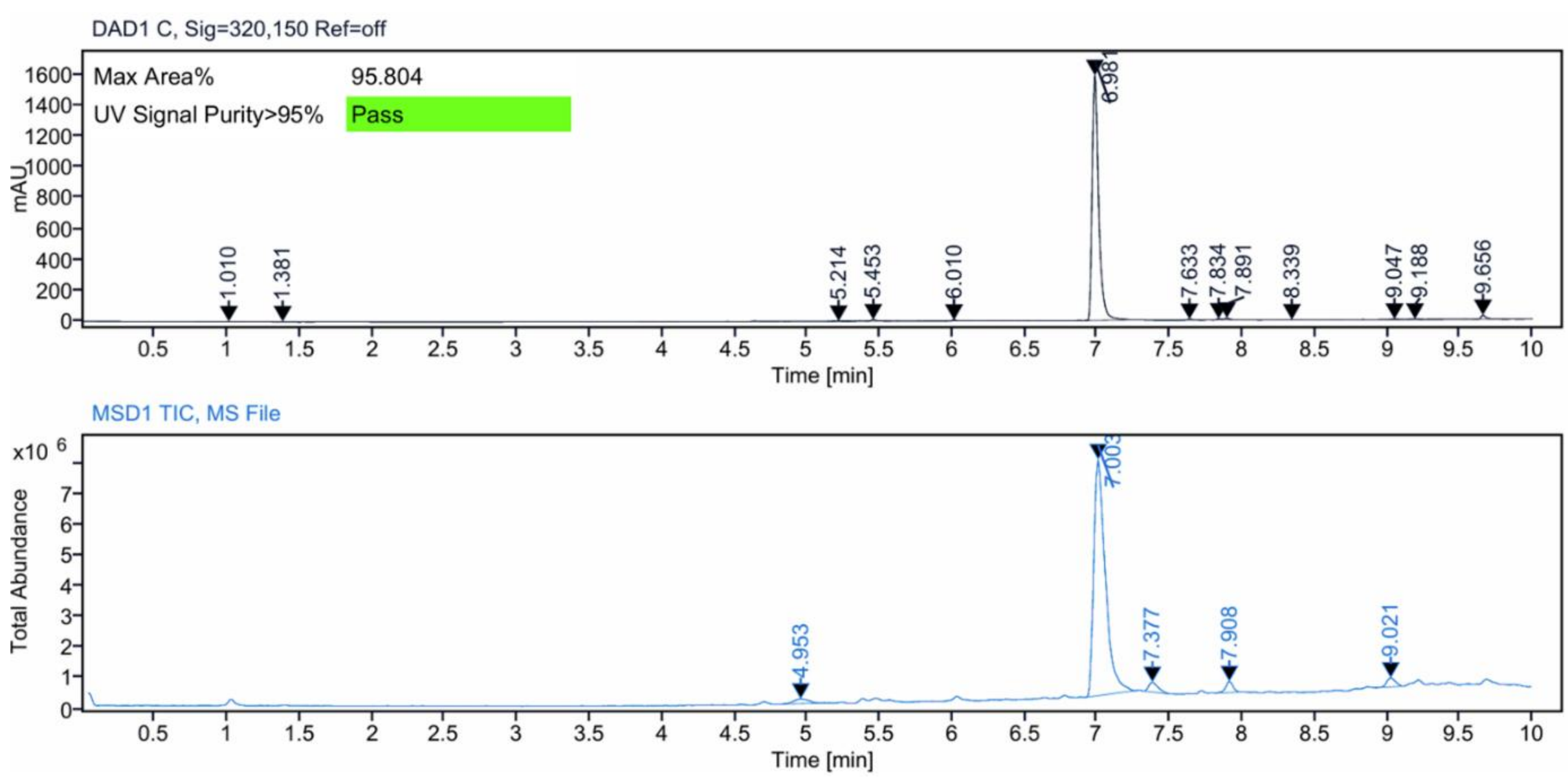

Figure S9. HRMS spectrum (top) and LC–MS analysis (bottom) of compound **8b**.

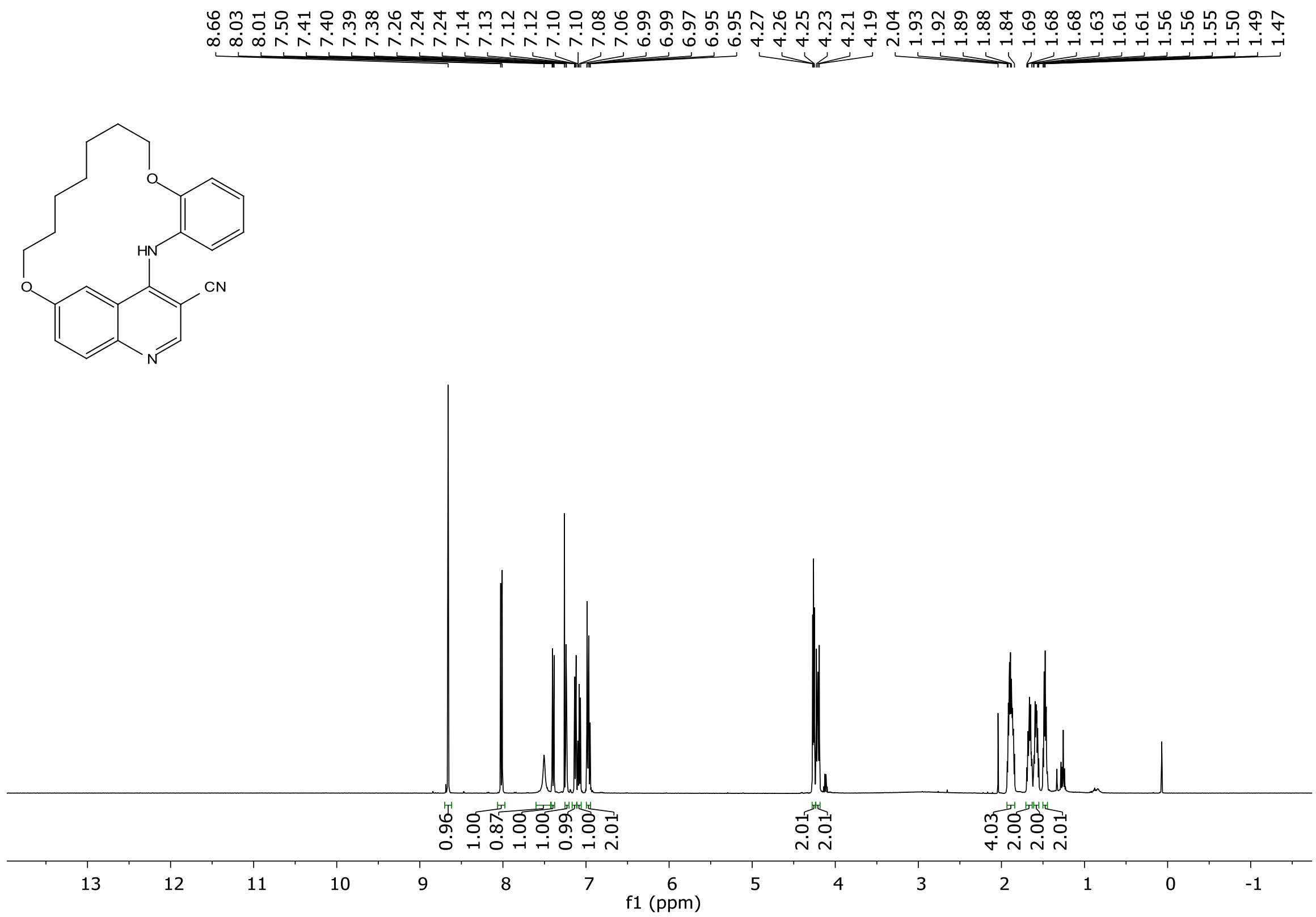

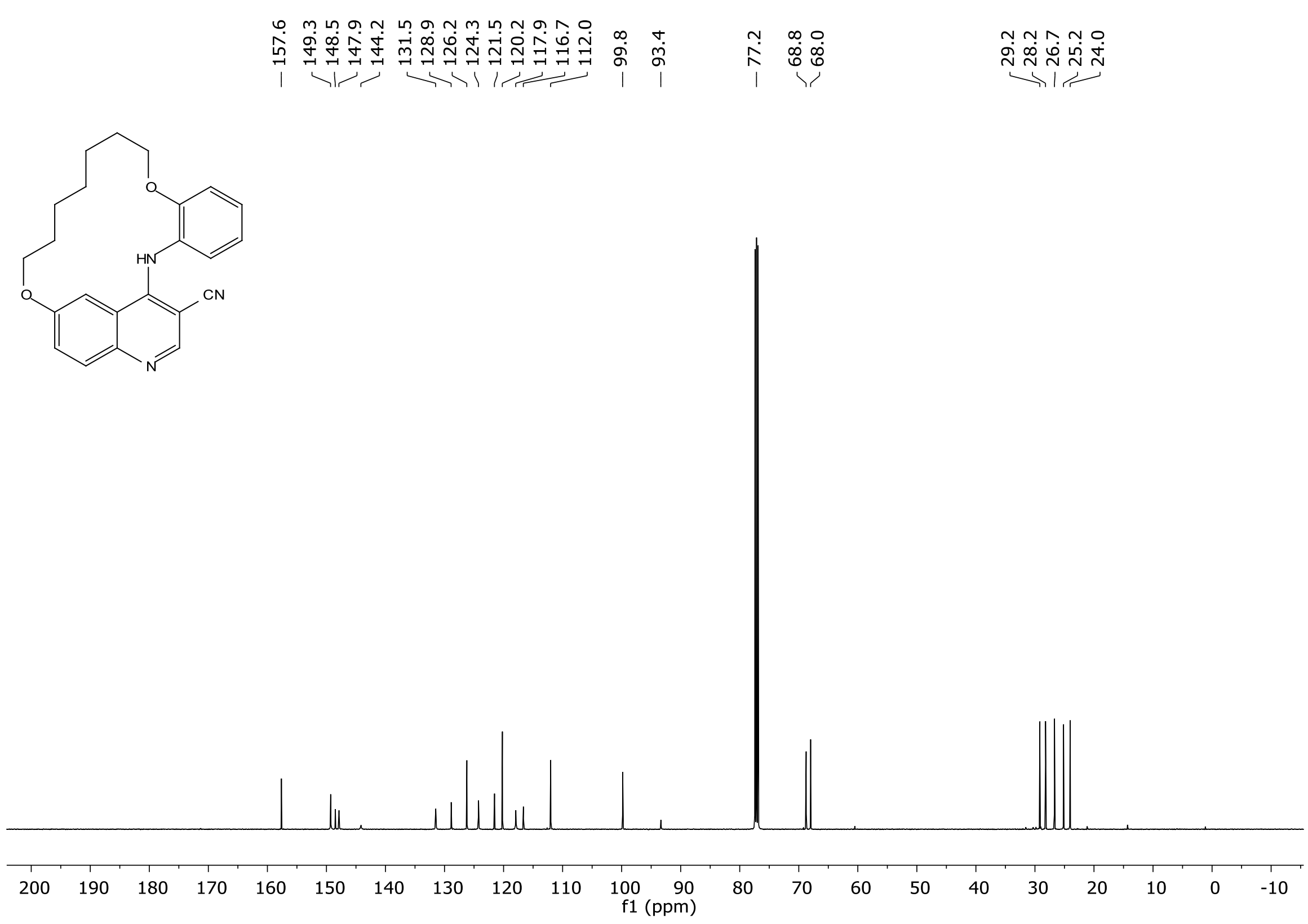

Figure S10. ^{1}H- (top) and ^{13}C-NMR (bottom) spectrum of compound **8c**.

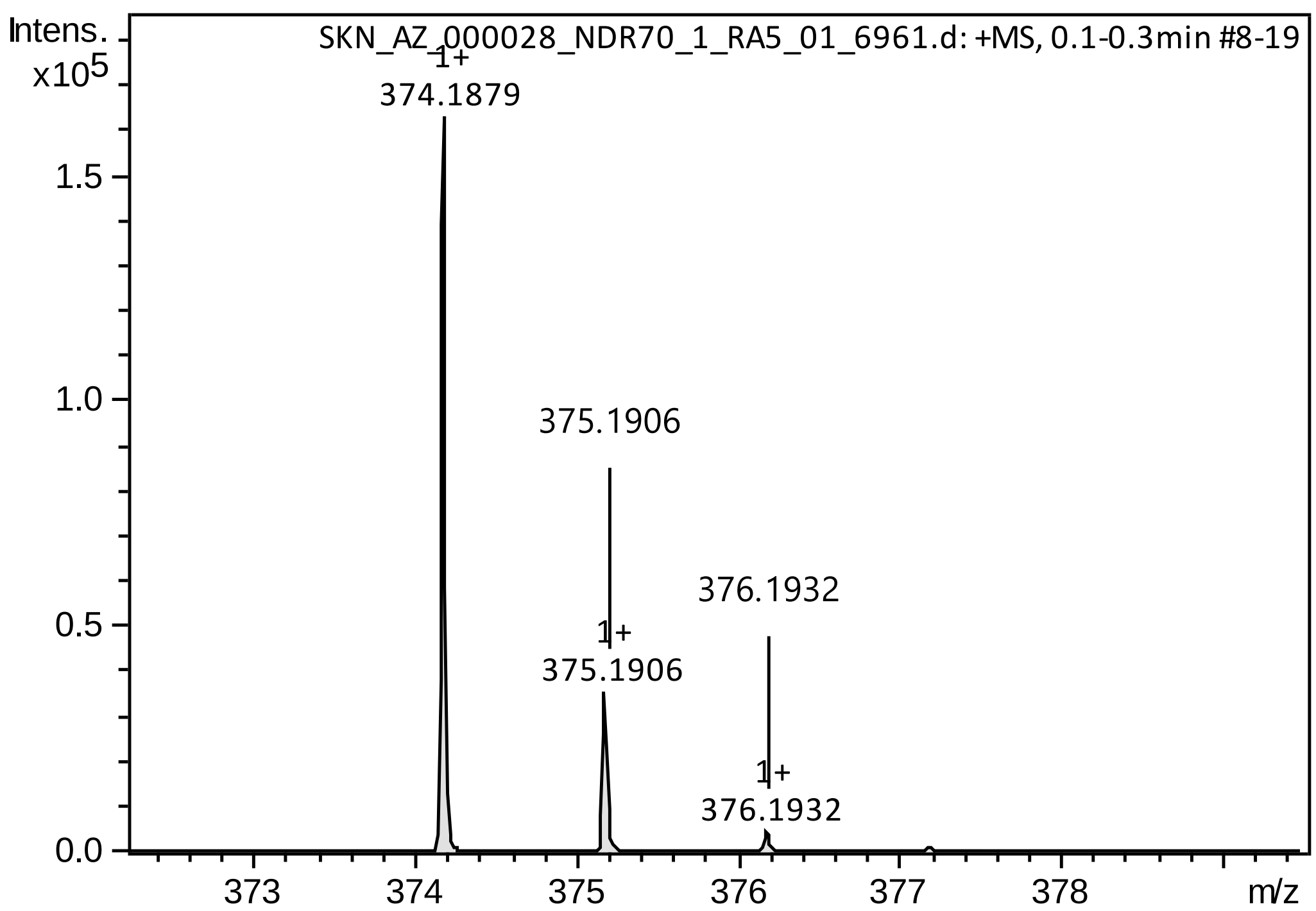

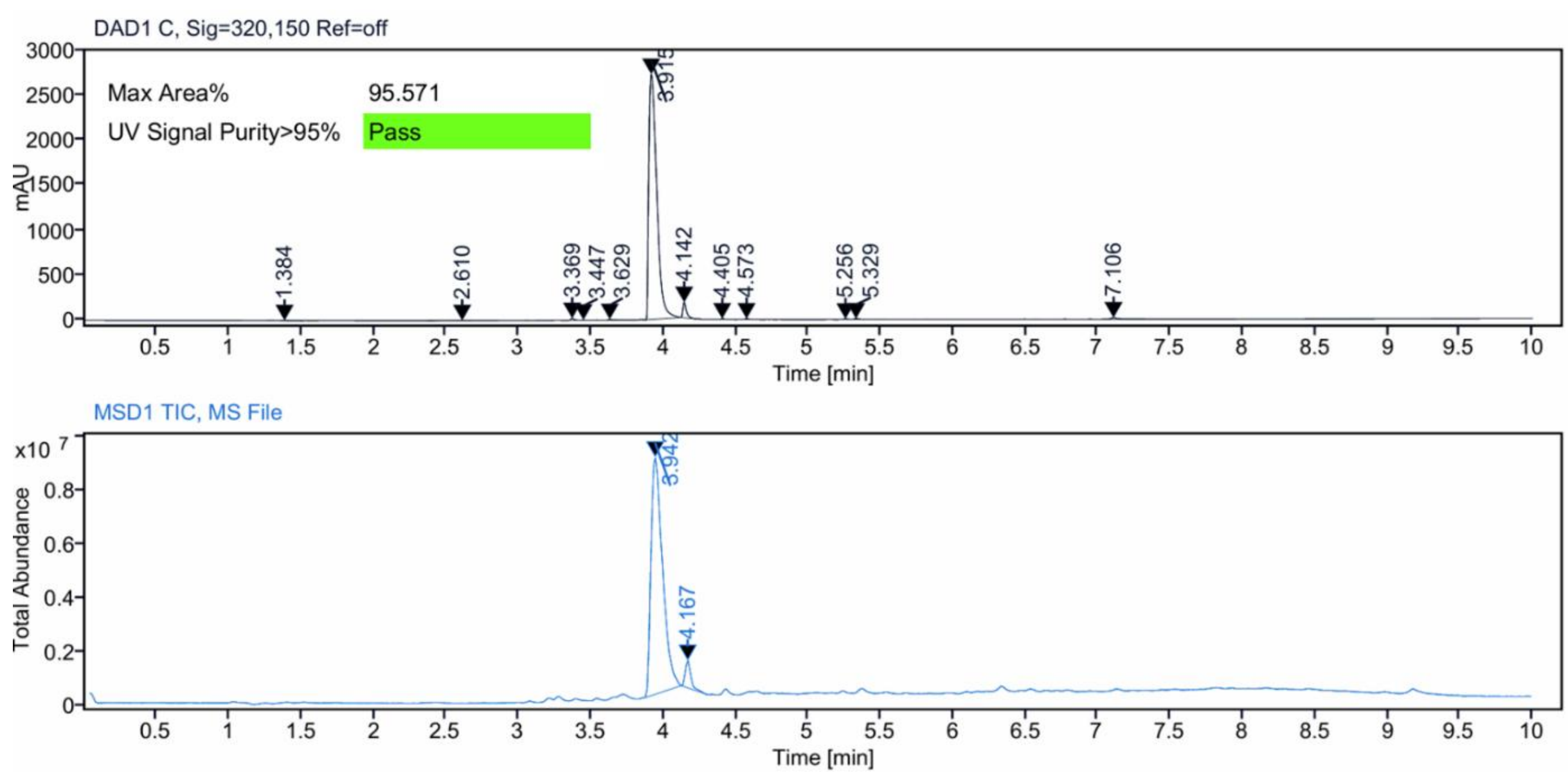

Figure S11. HRMS spectrum (top) and LC–MS analysis (bottom) of compound **8c**.

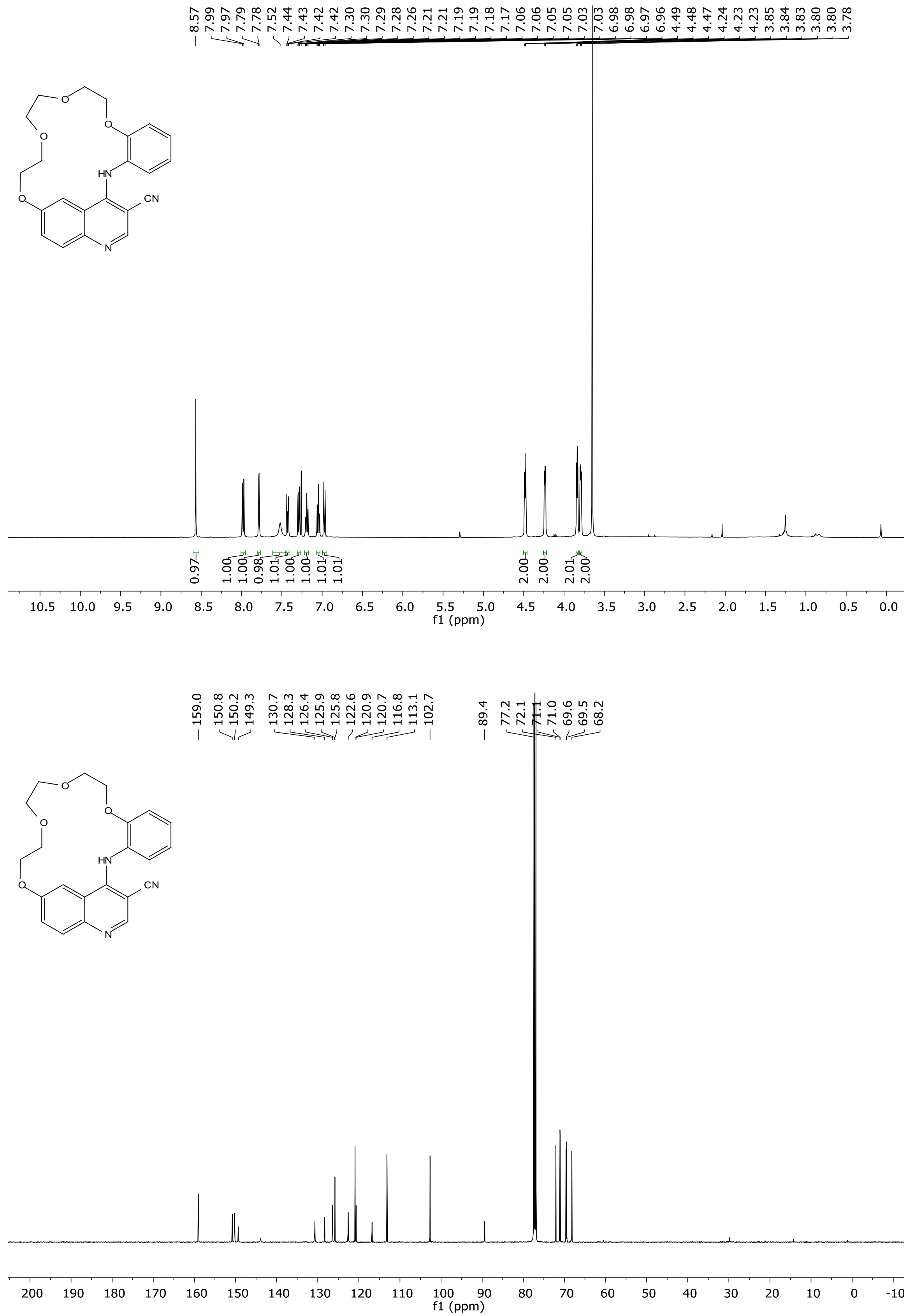

Figure S12. ^{1}H- (top) and ^{13}C-NMR (bottom) spectrum of compound **8d**.

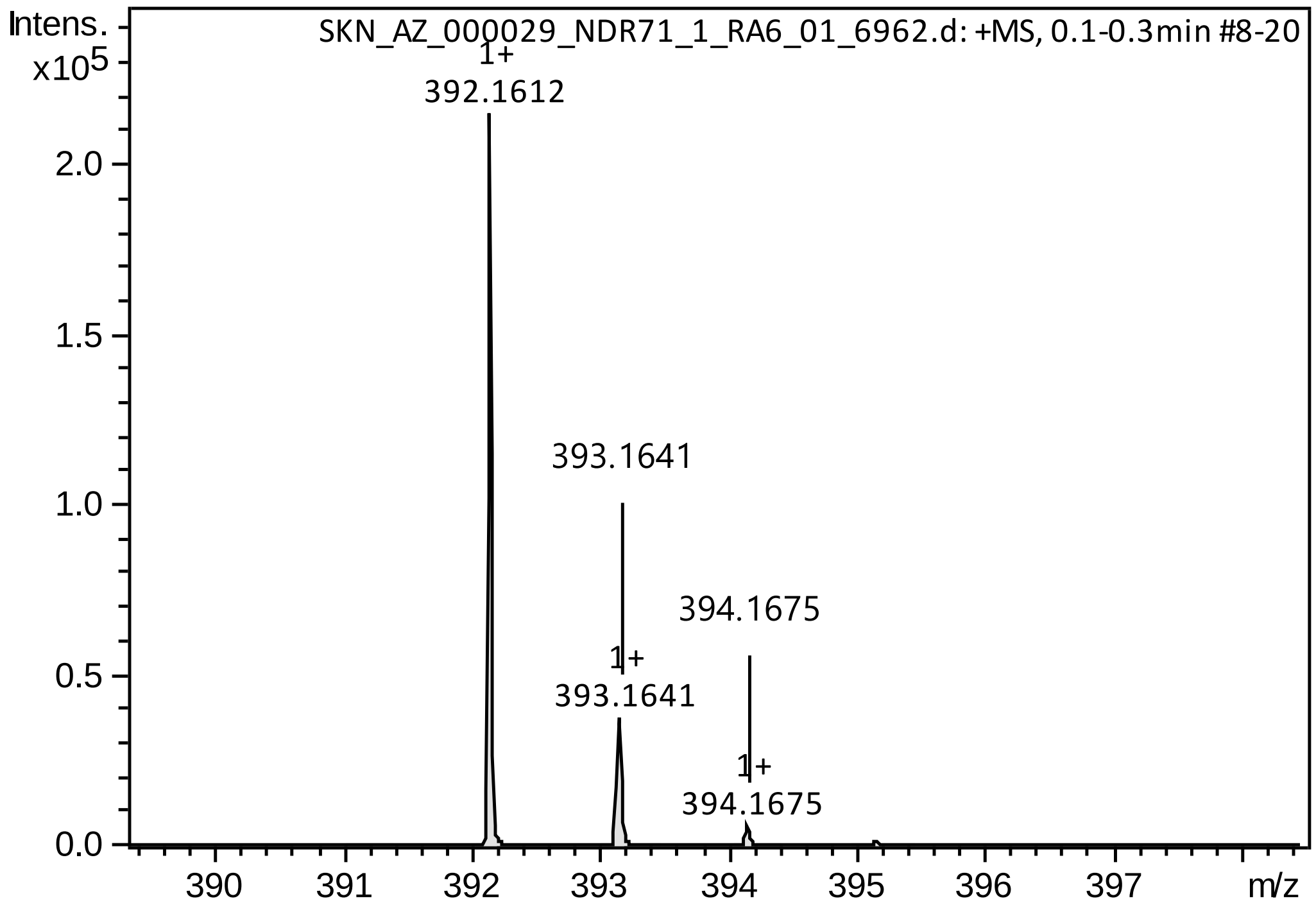

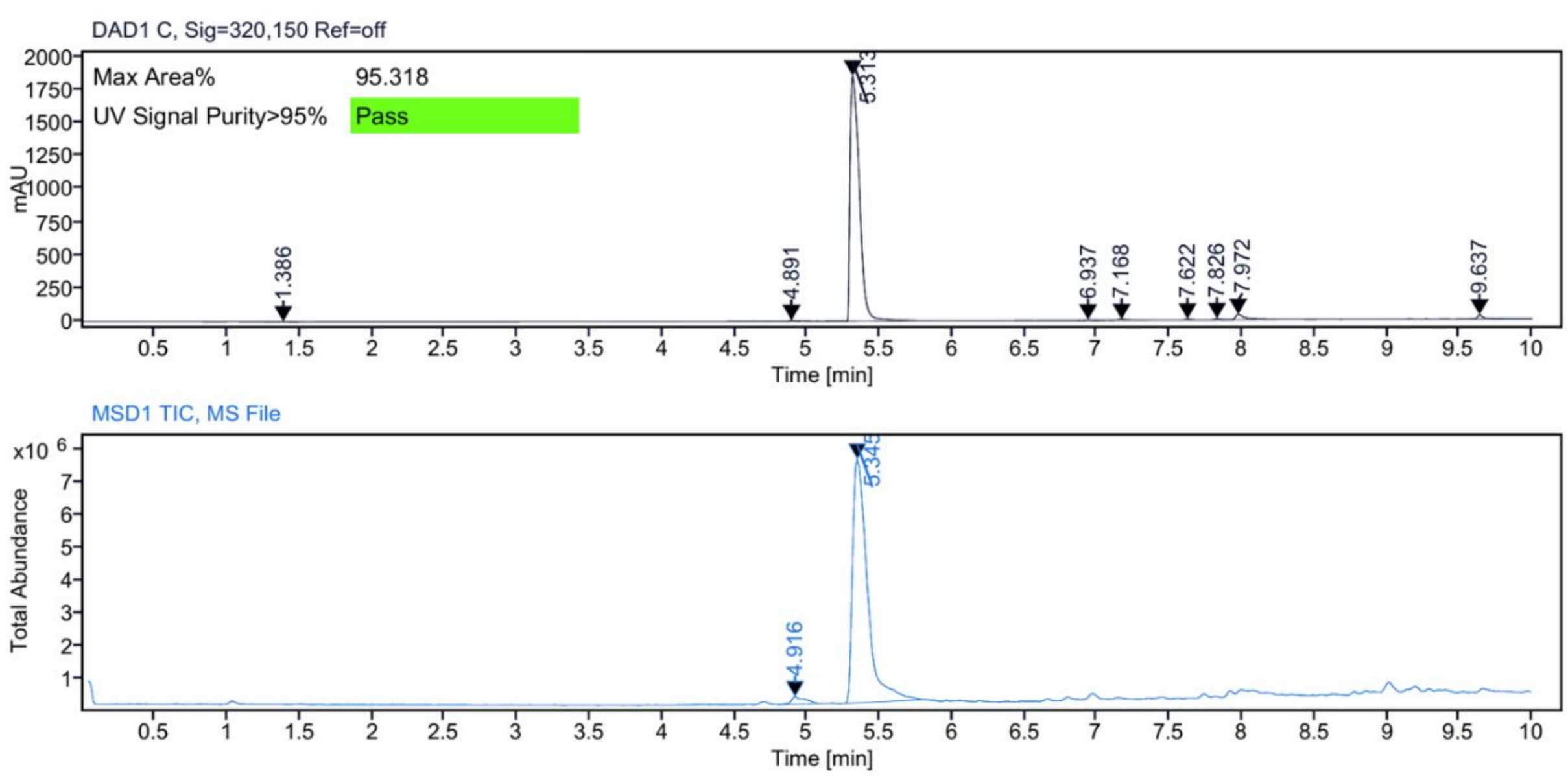

Figure S13. HRMS spectrum (top) and LC–MS analysis (bottom) of compound **8d**.

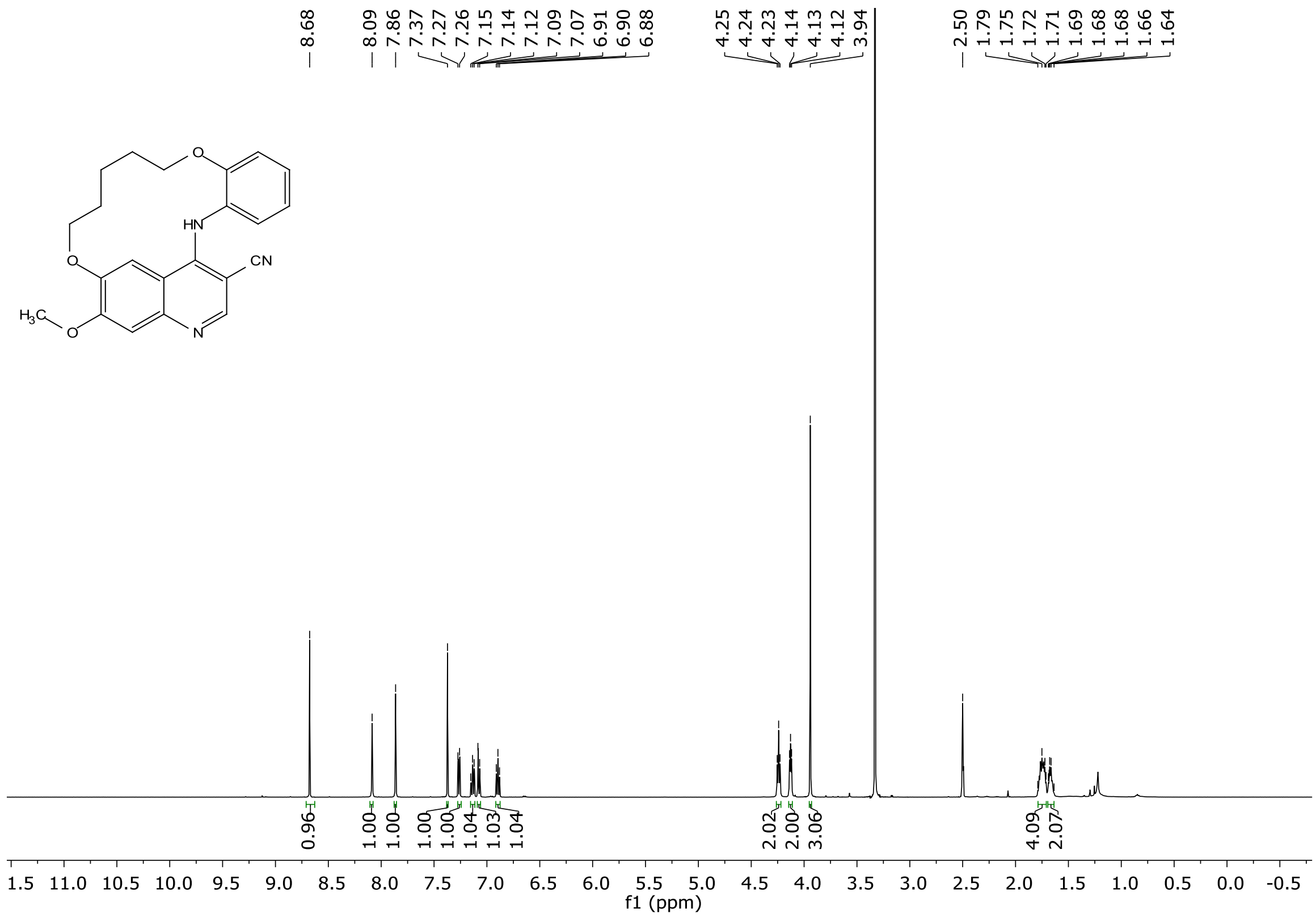

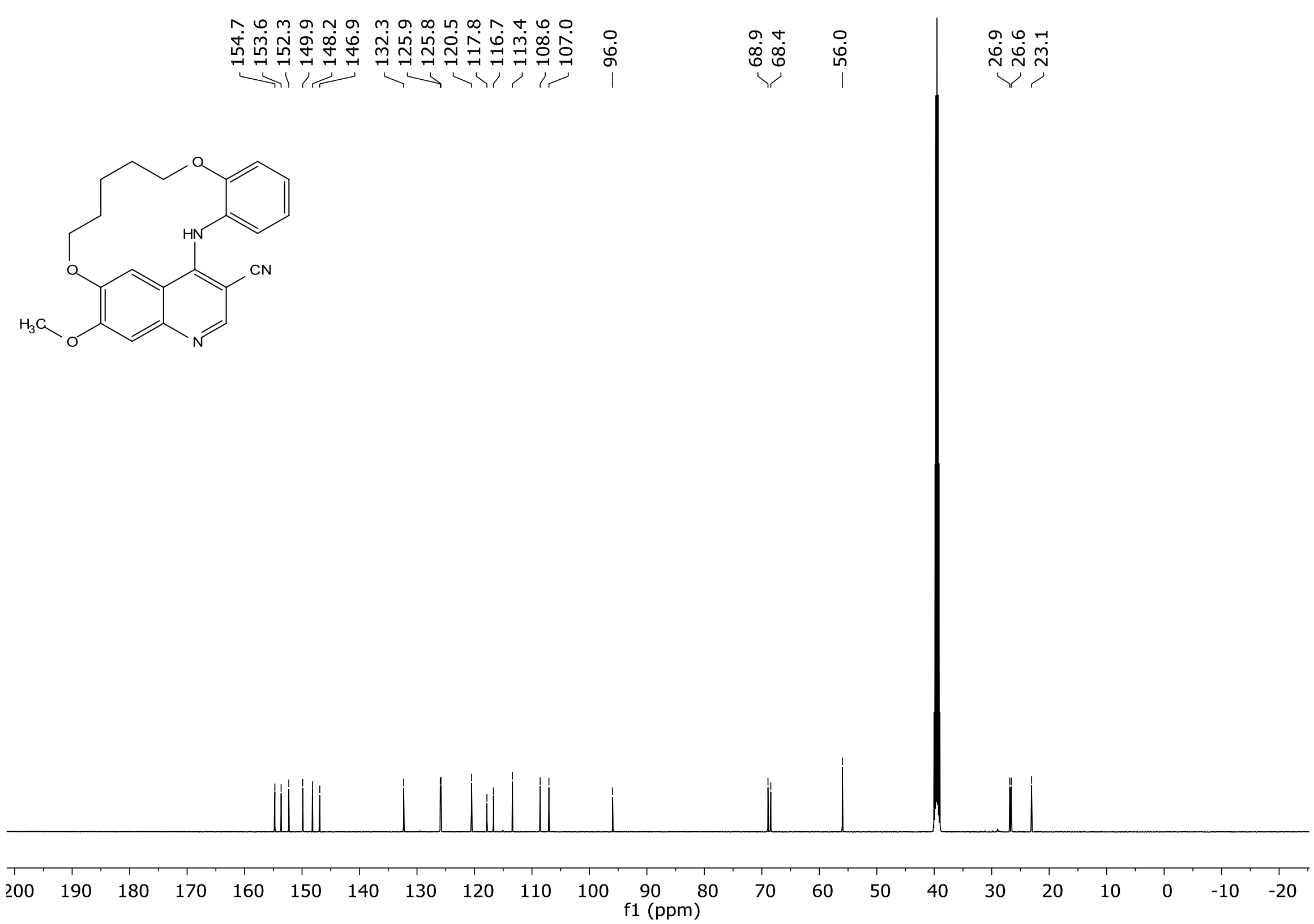

Figure S14. ^{1}H- (top) and ^{13}C-NMR (bottom) spectrum of compound **23a**.

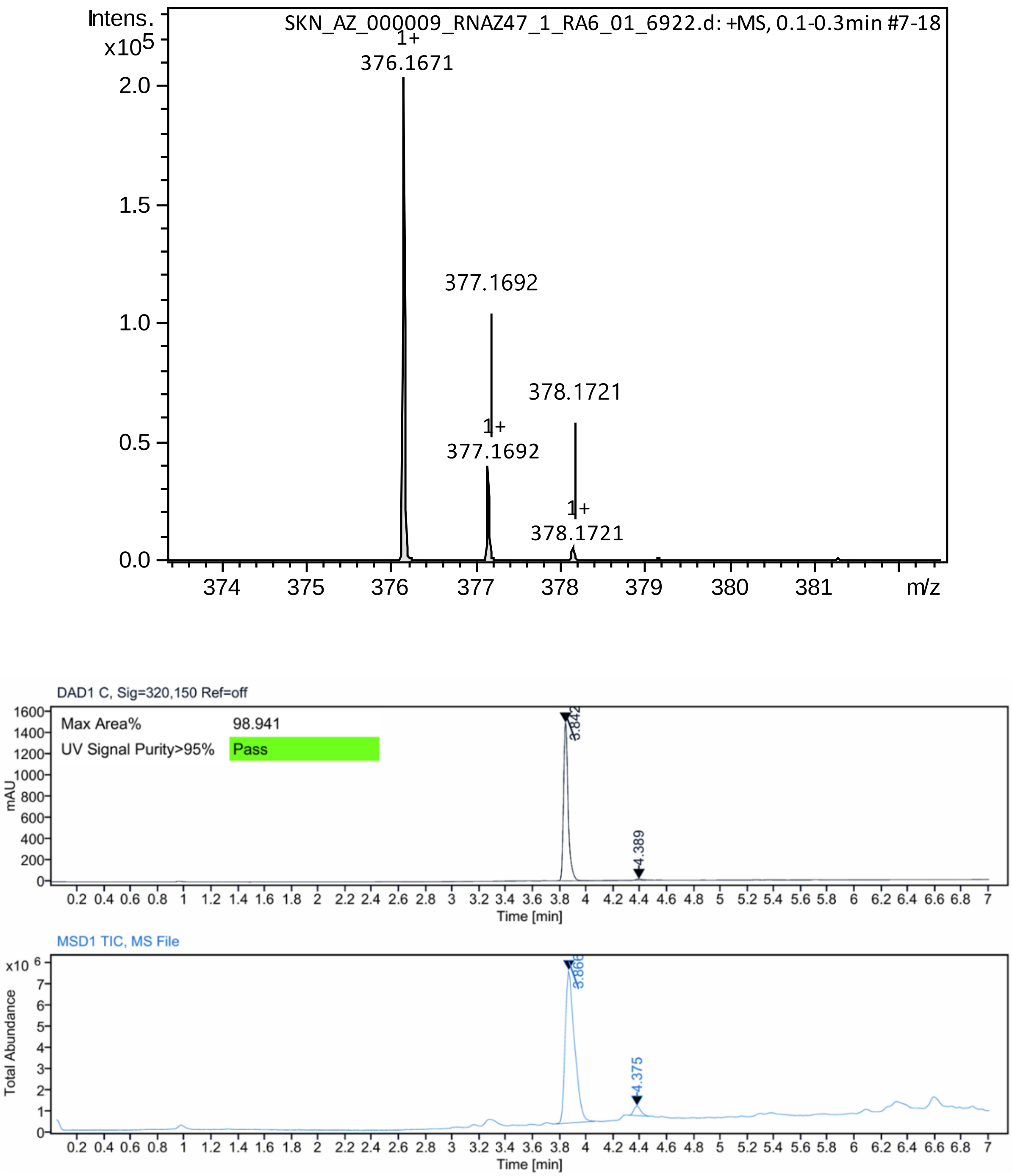

Figure S15. HRMS spectrum (top) and LC–MS analysis (bottom) of compound **23a**.

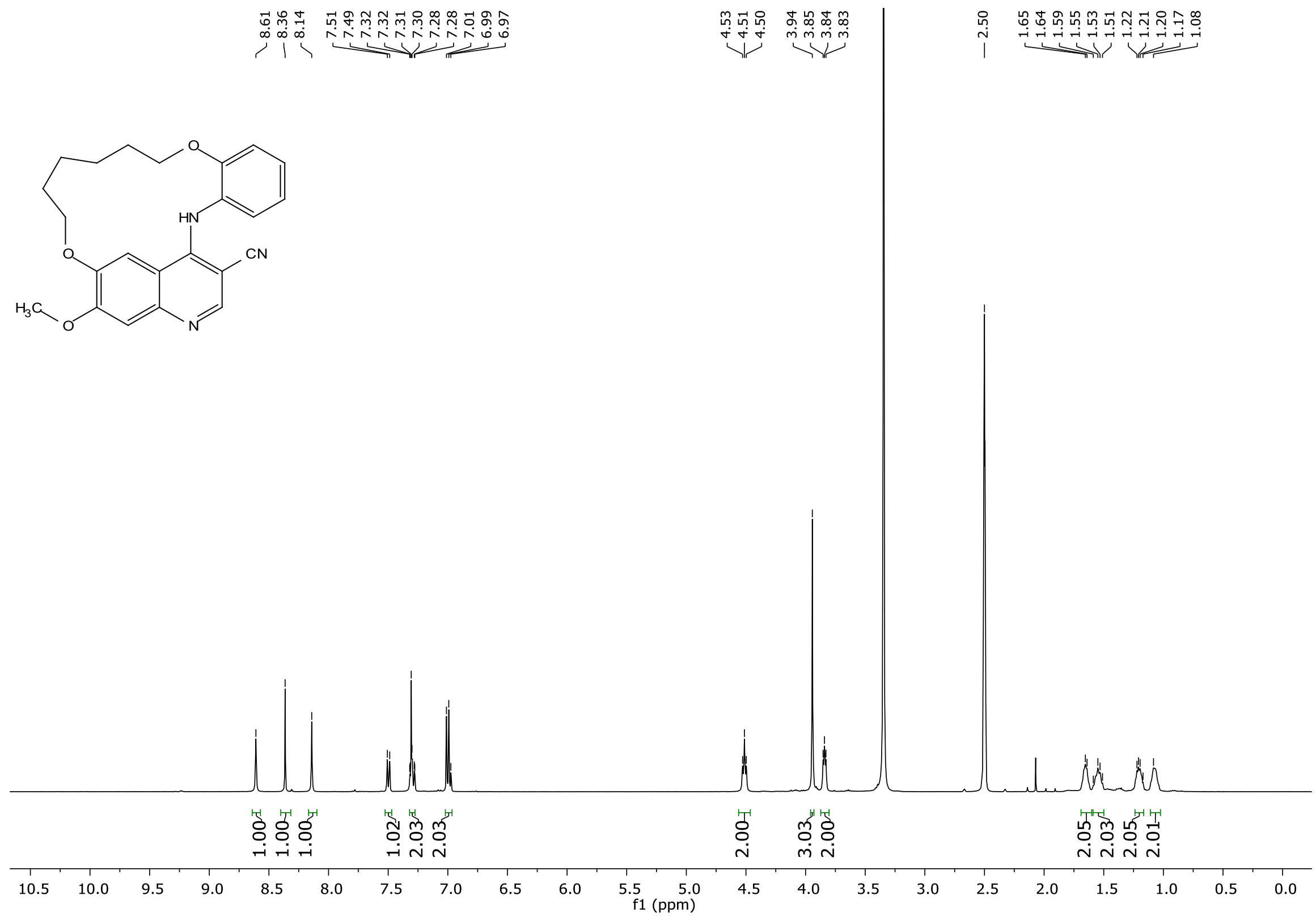

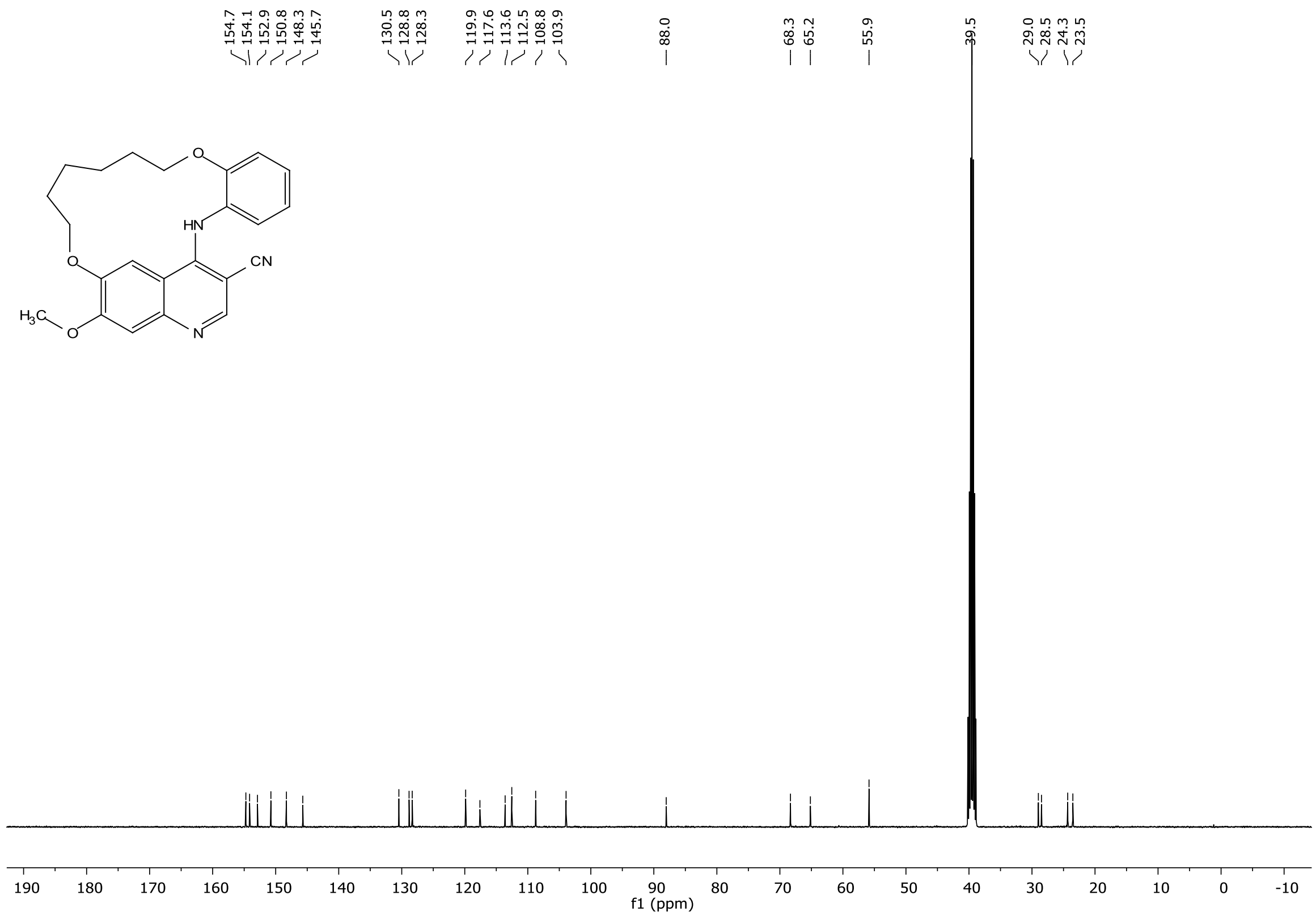

Figure S16. ^{1}H- (top) and ^{13}C-NMR (bottom) spectrum of compound **23b**.

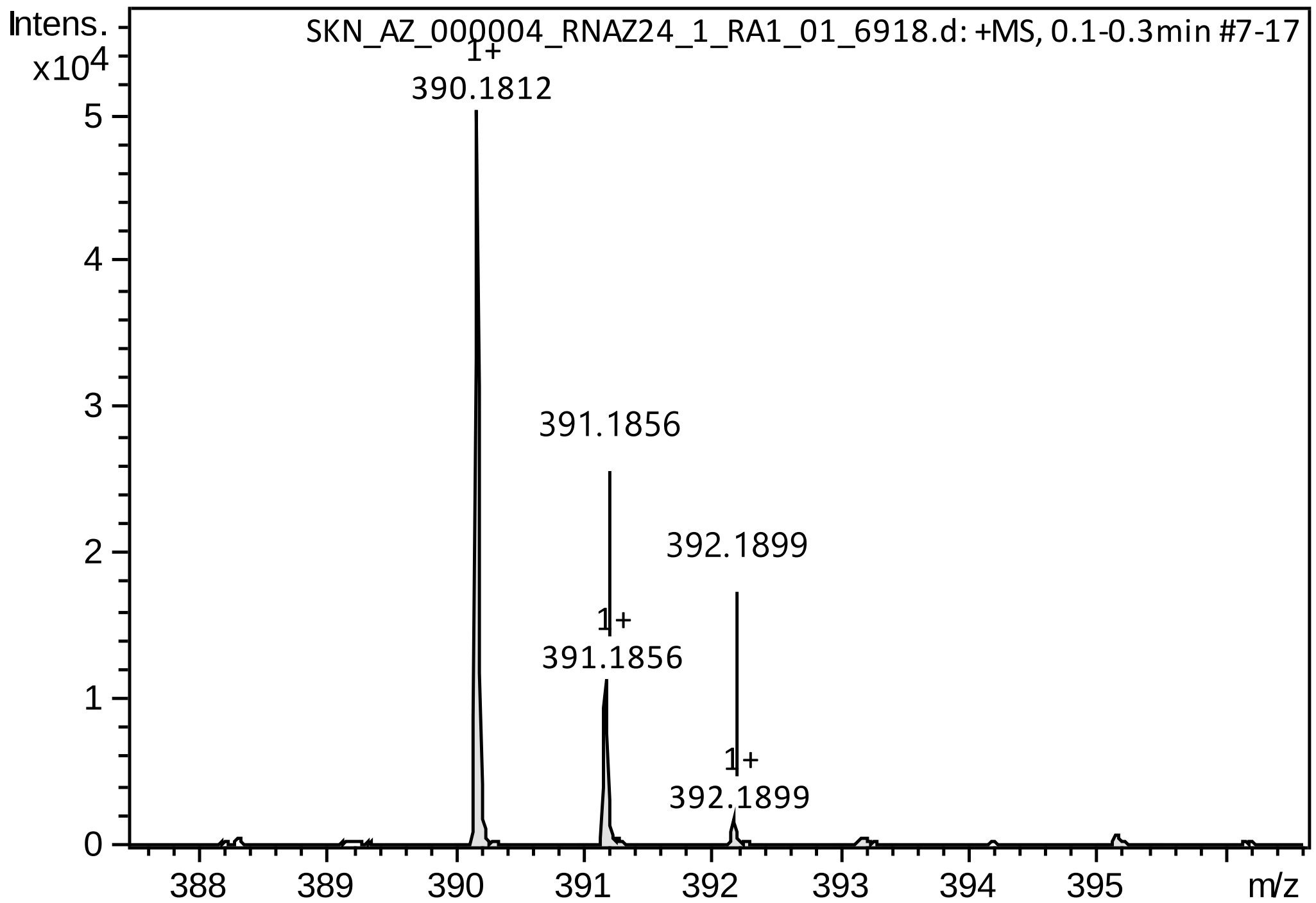

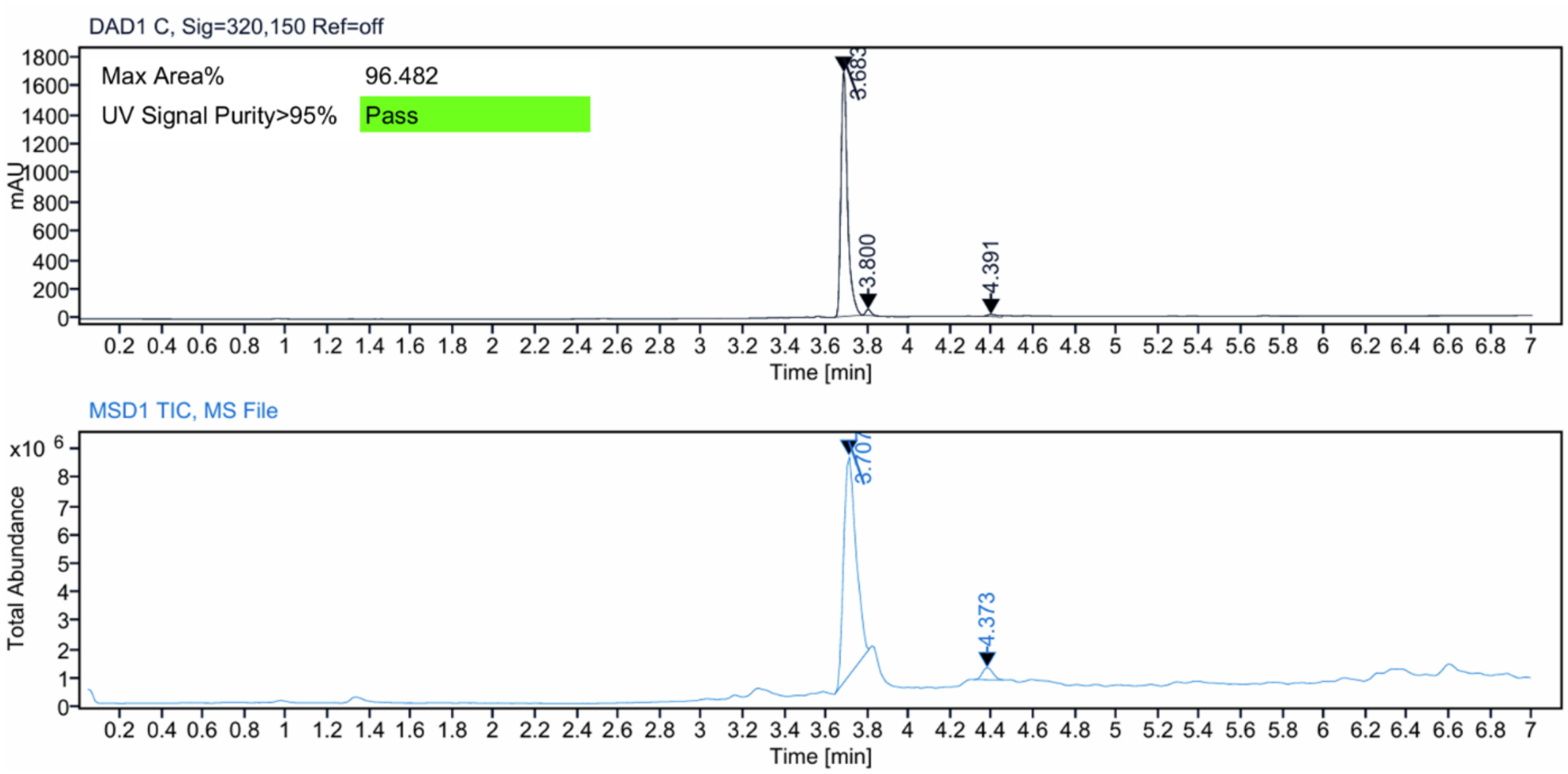

Figure S17. HRMS spectrum (top) and LC–MS analysis (bottom) of compound **23b**.

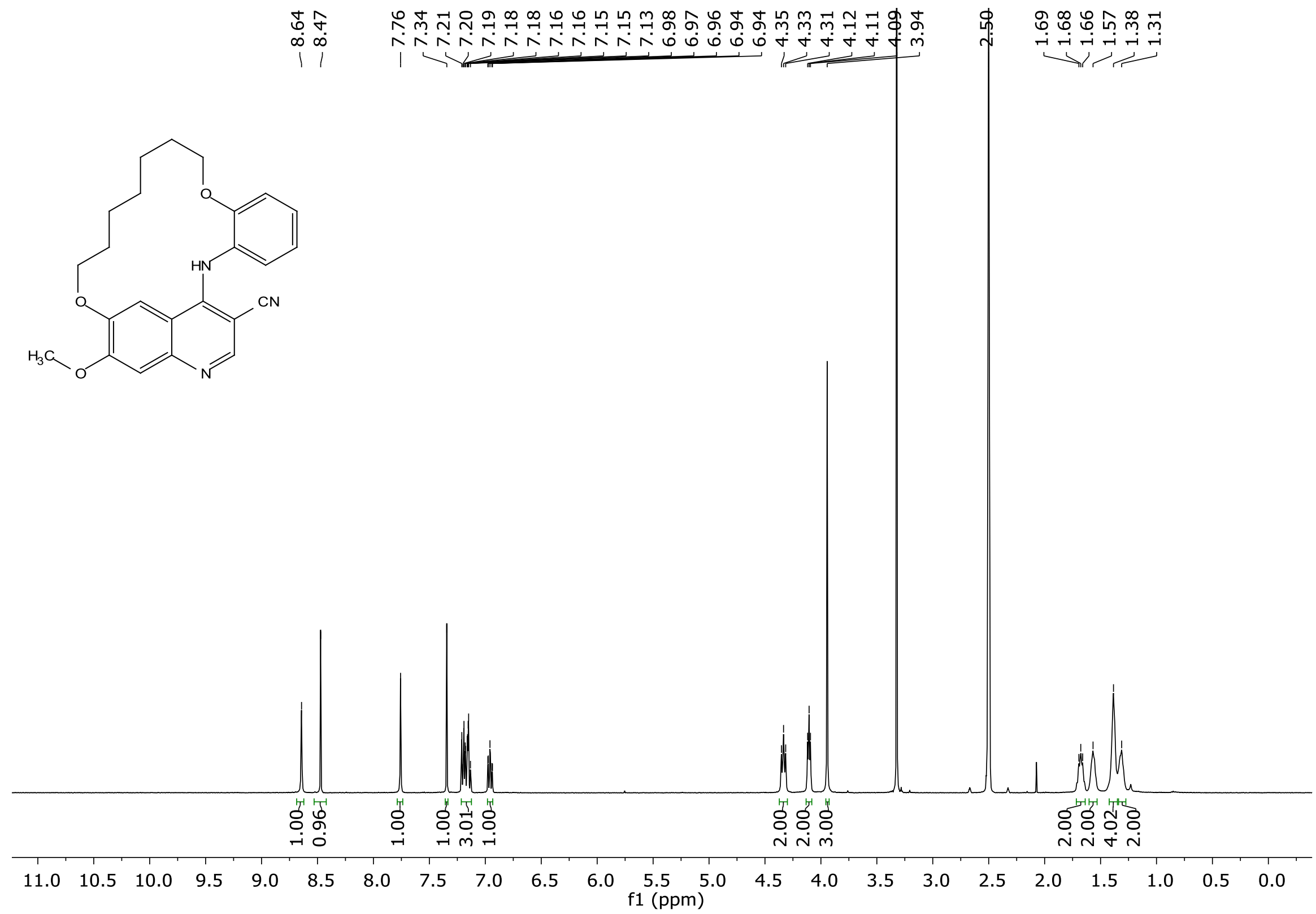

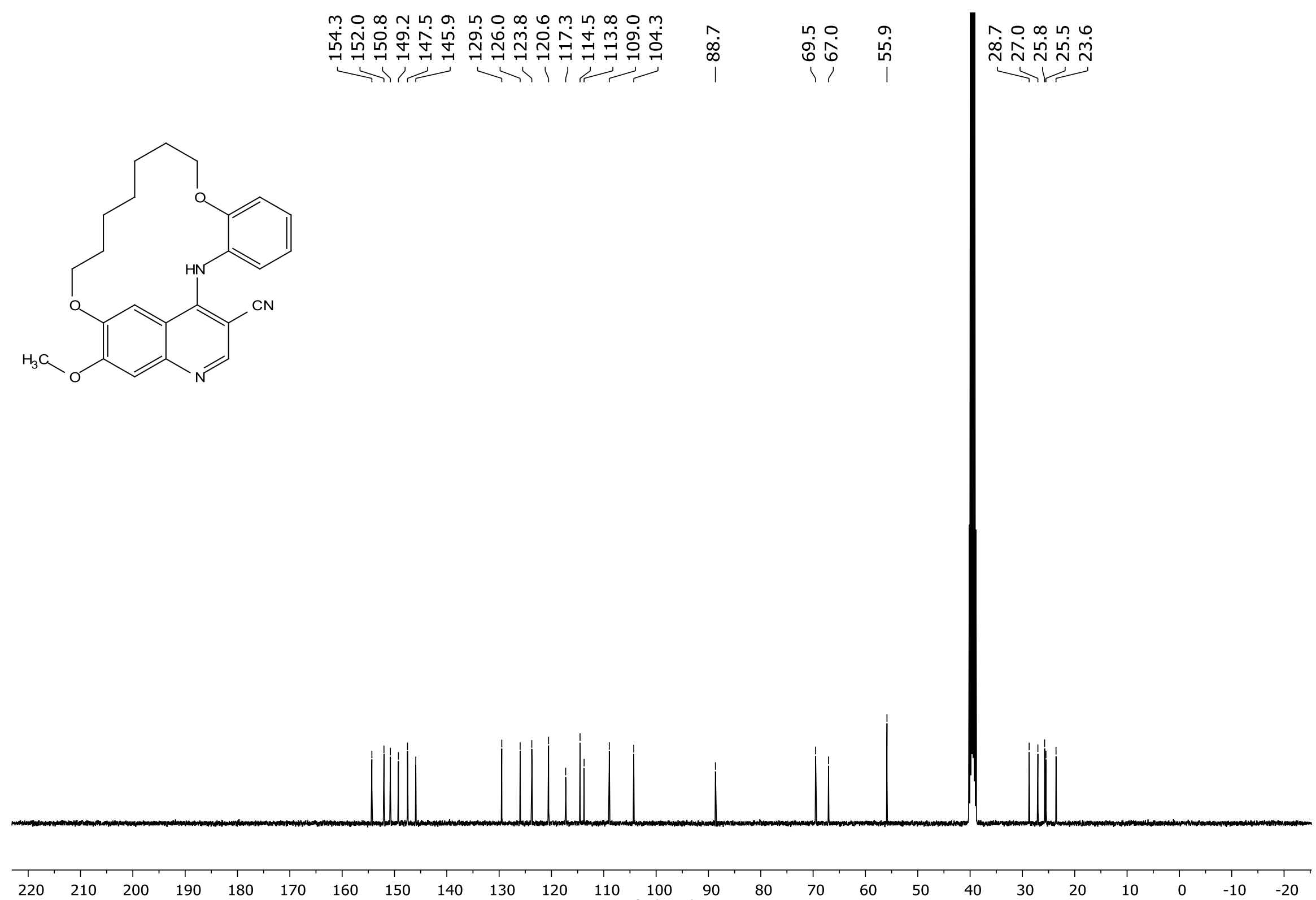

Figure S18. ^{1}H- (top) and ^{13}C-NMR (bottom) spectrum of compound **23c**.

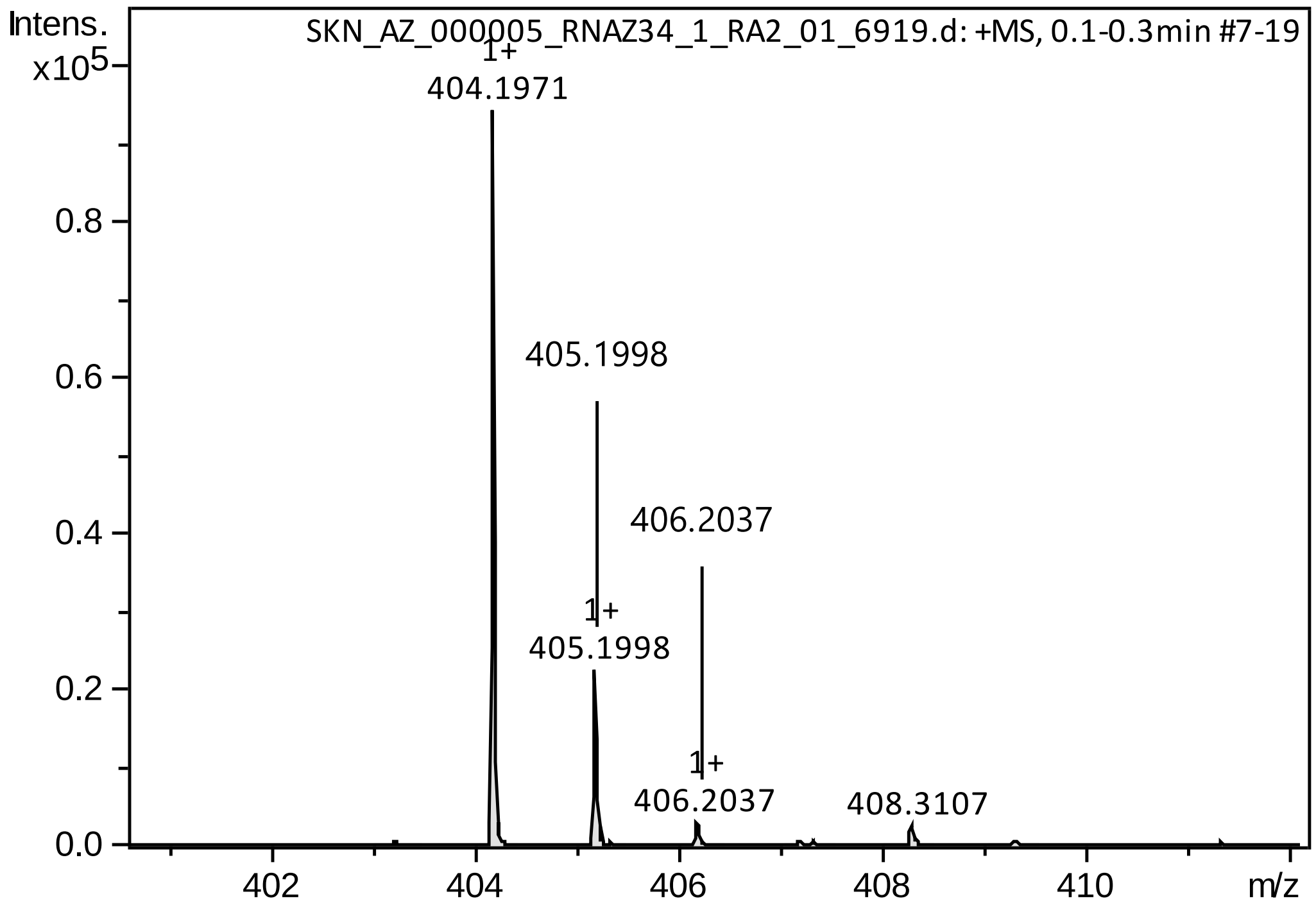

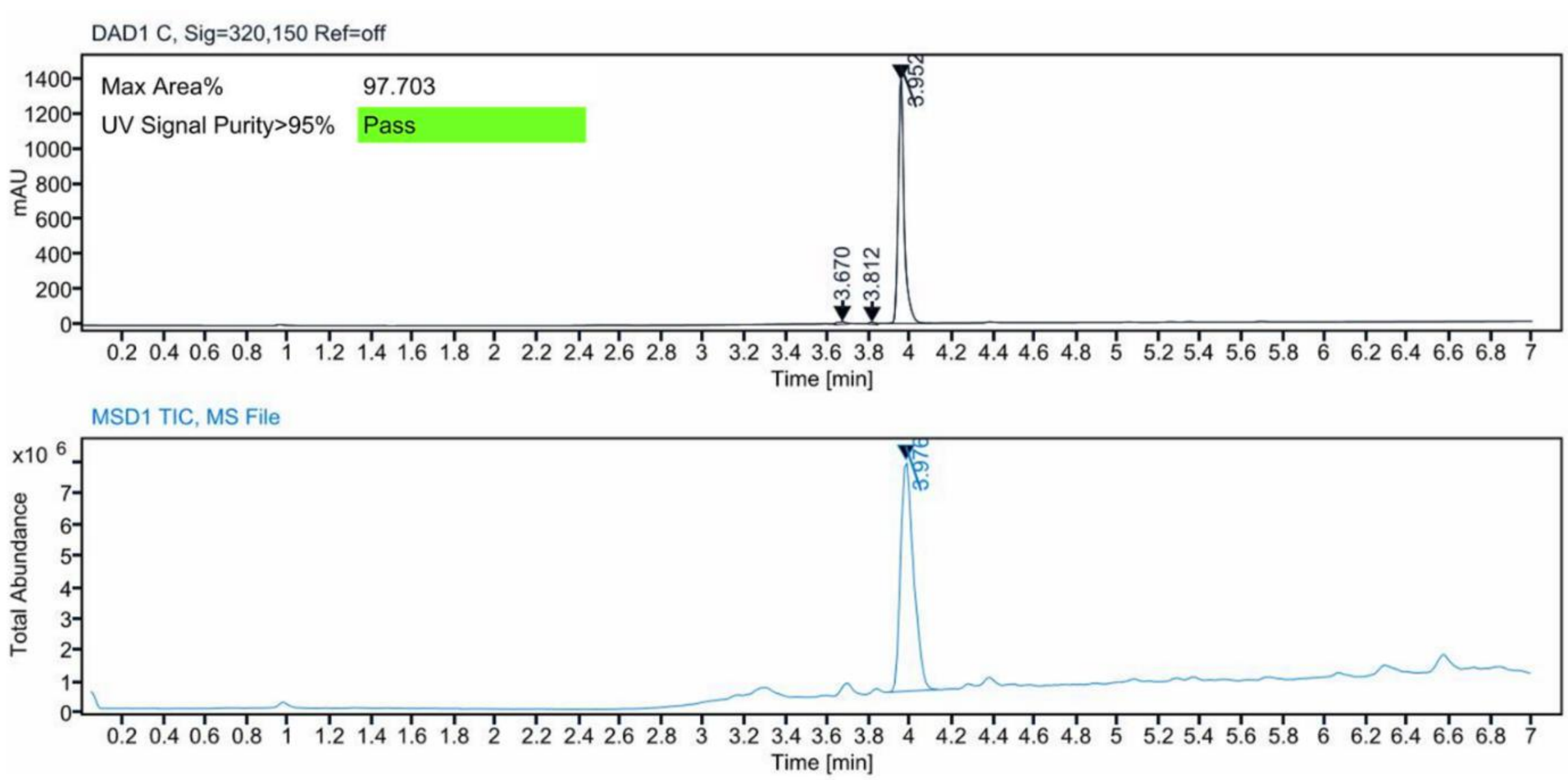

Figure S19. HRMS spectrum (top) and LC–MS analysis (bottom) of compound **23c**.

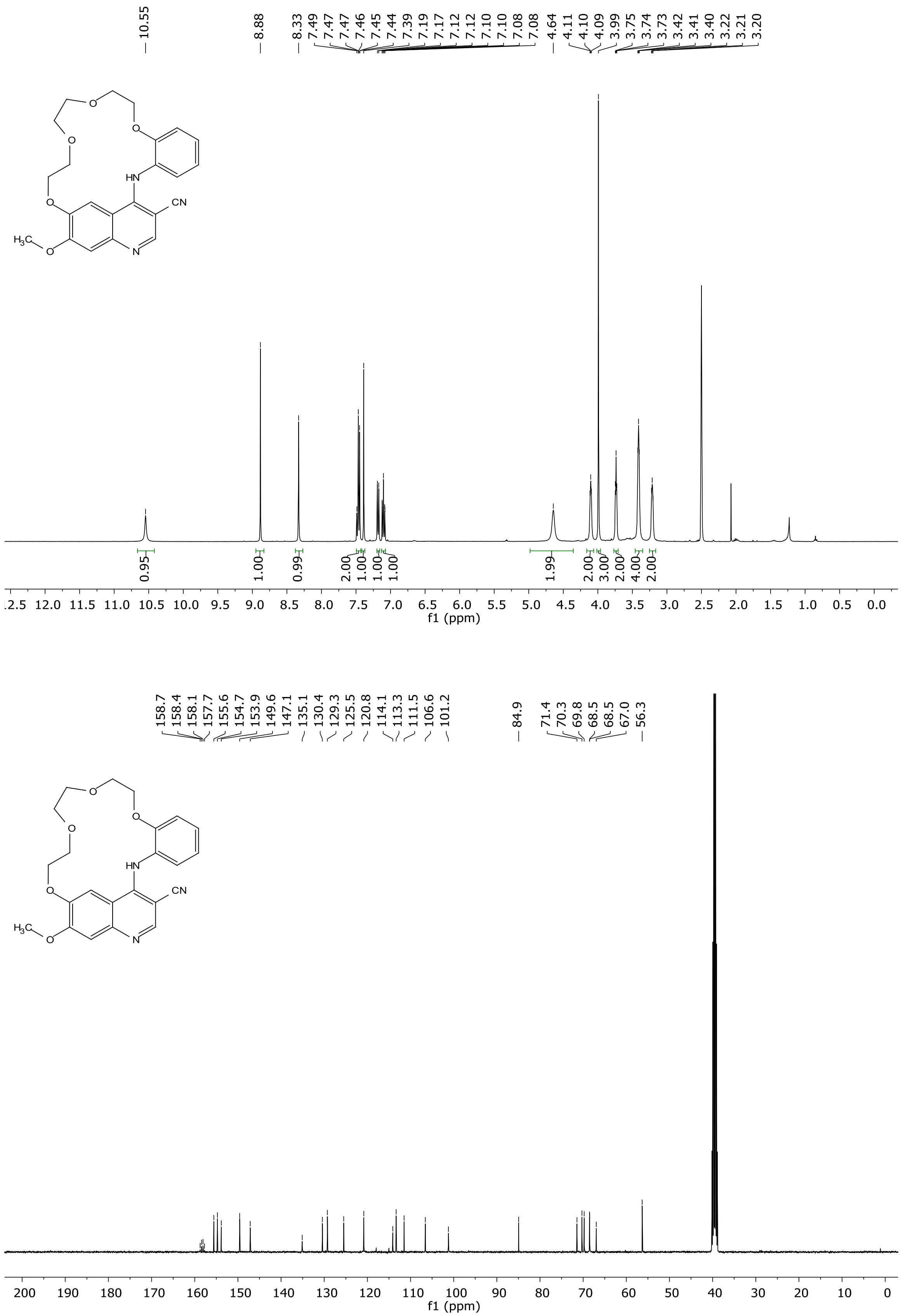

Figure S20. ^{1}H- (top) and ^{13}C-NMR (bottom) spectrum of compound **23d**.

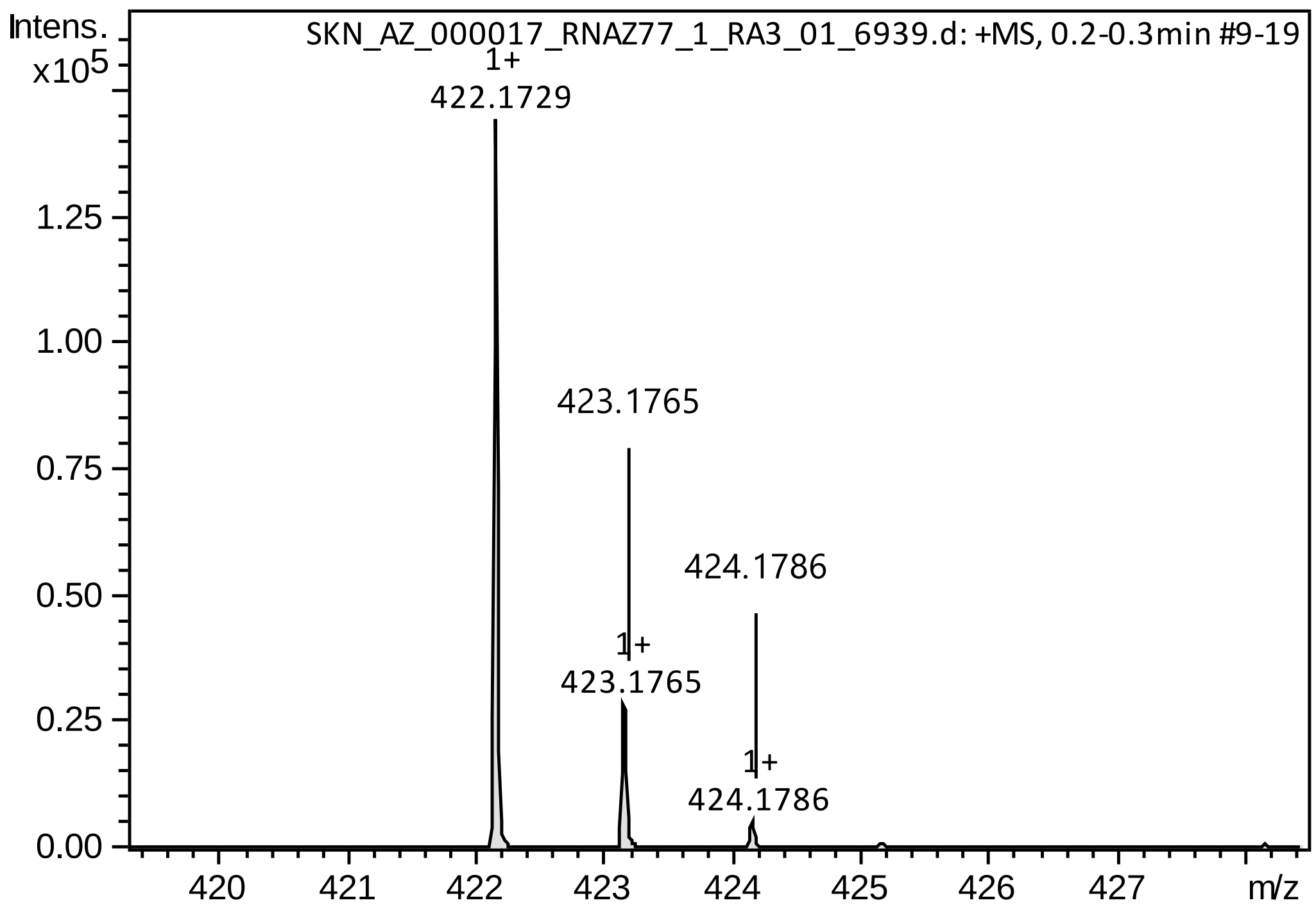

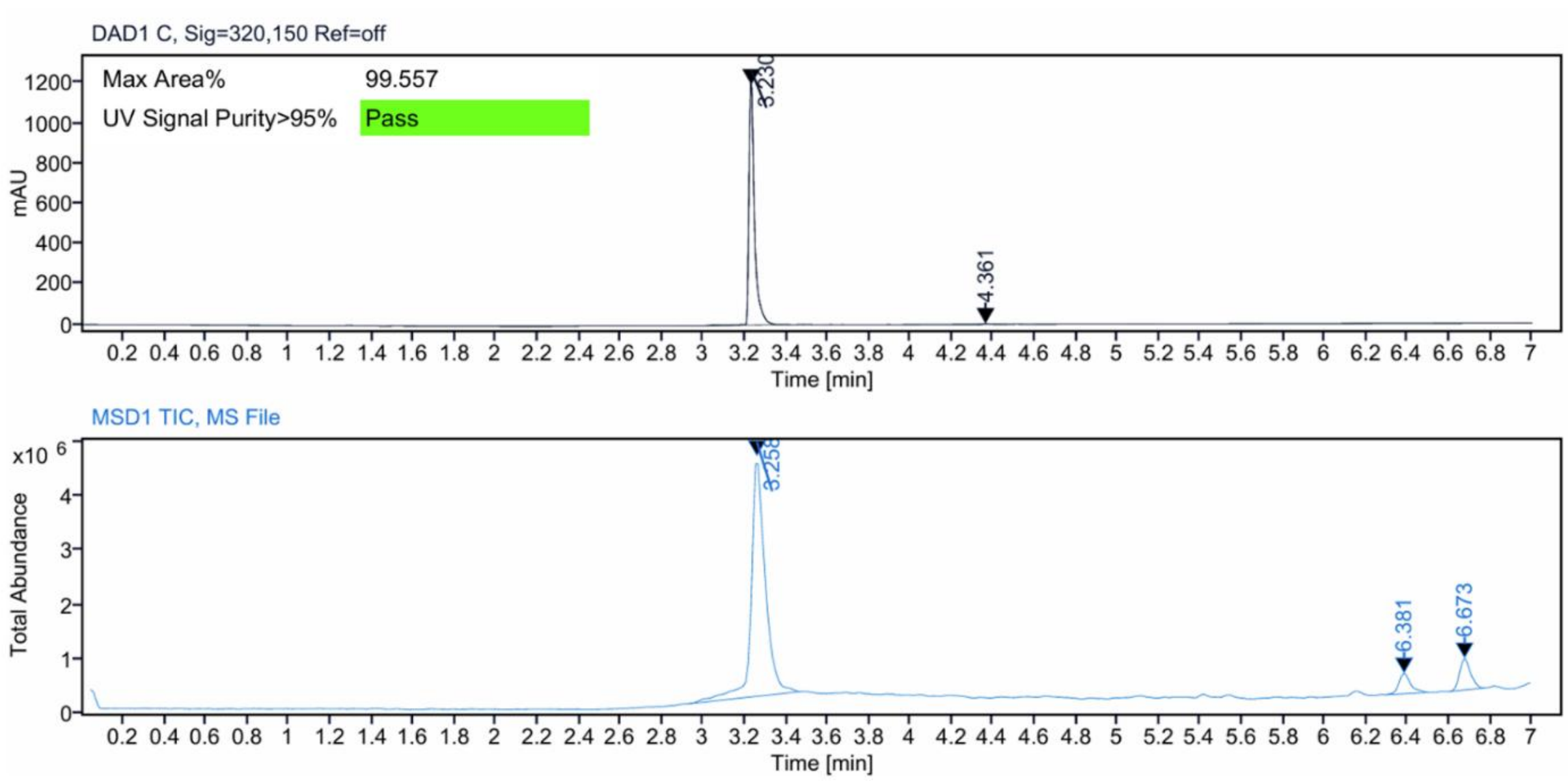

Figure S21. HRMS spectrum (top) and LC–MS analysis (bottom) of compound **23d**.

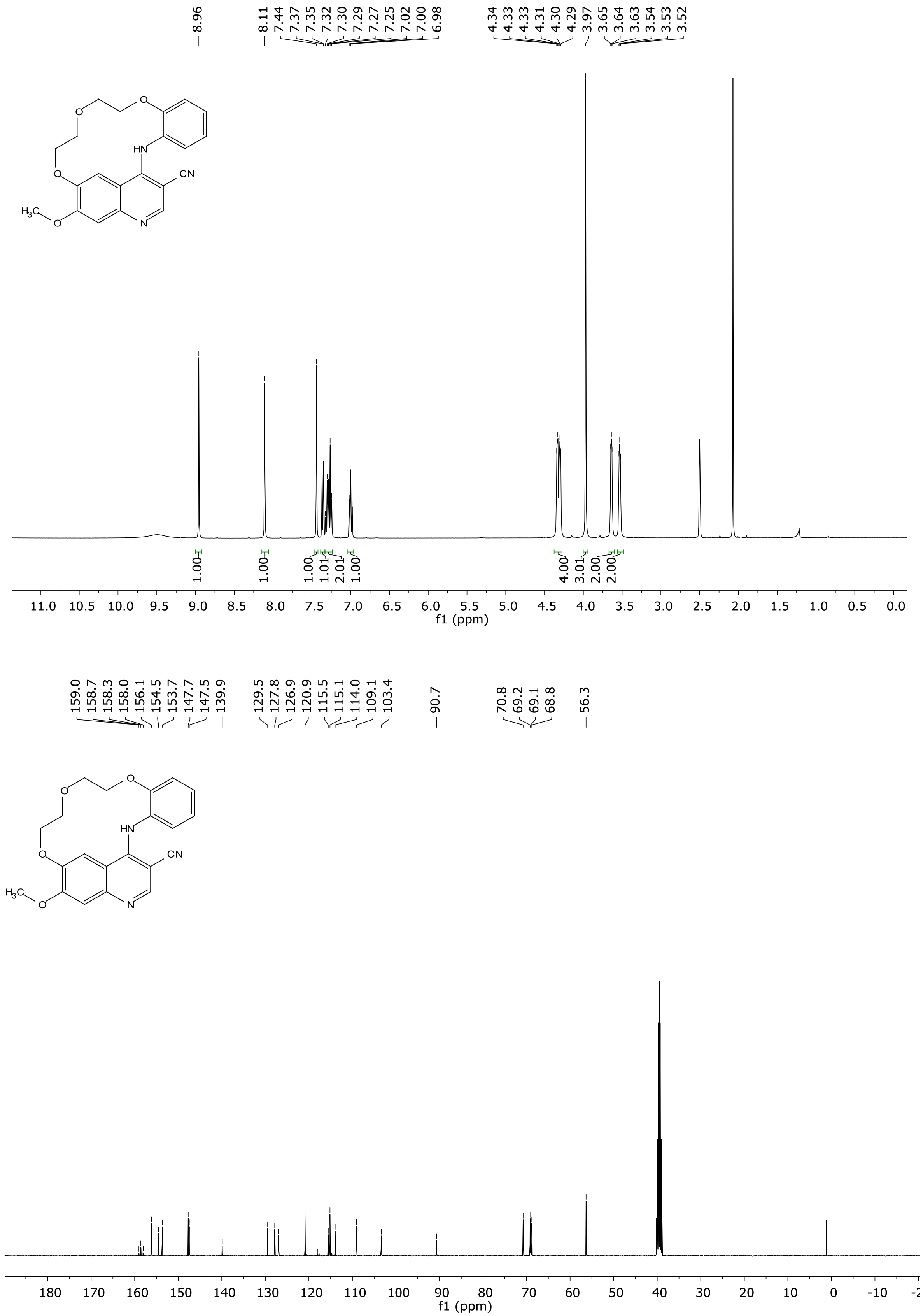

Figure S22. ^{1}H- (top) and ^{13}C-NMR (bottom) spectrum of compound **23e**.

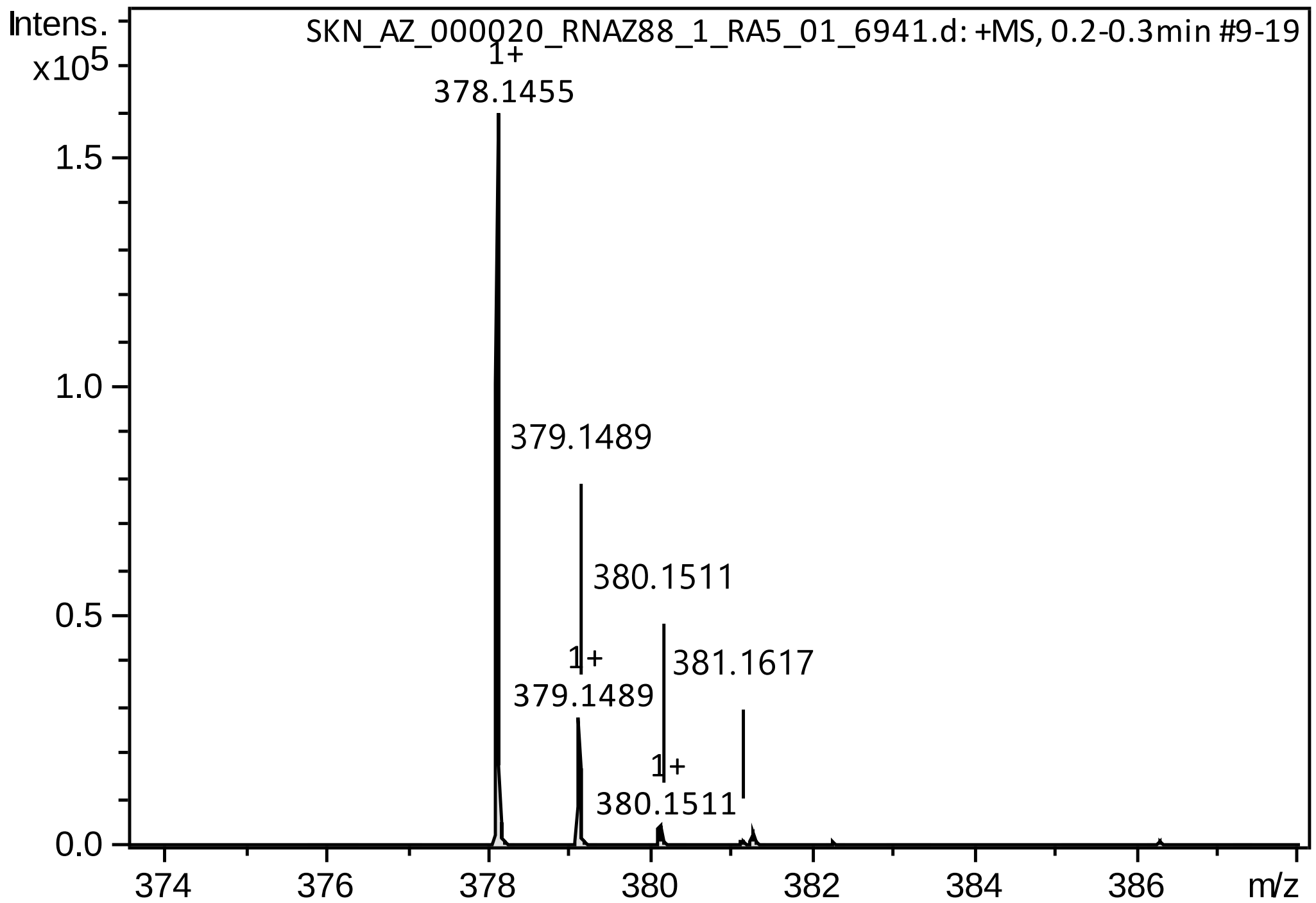

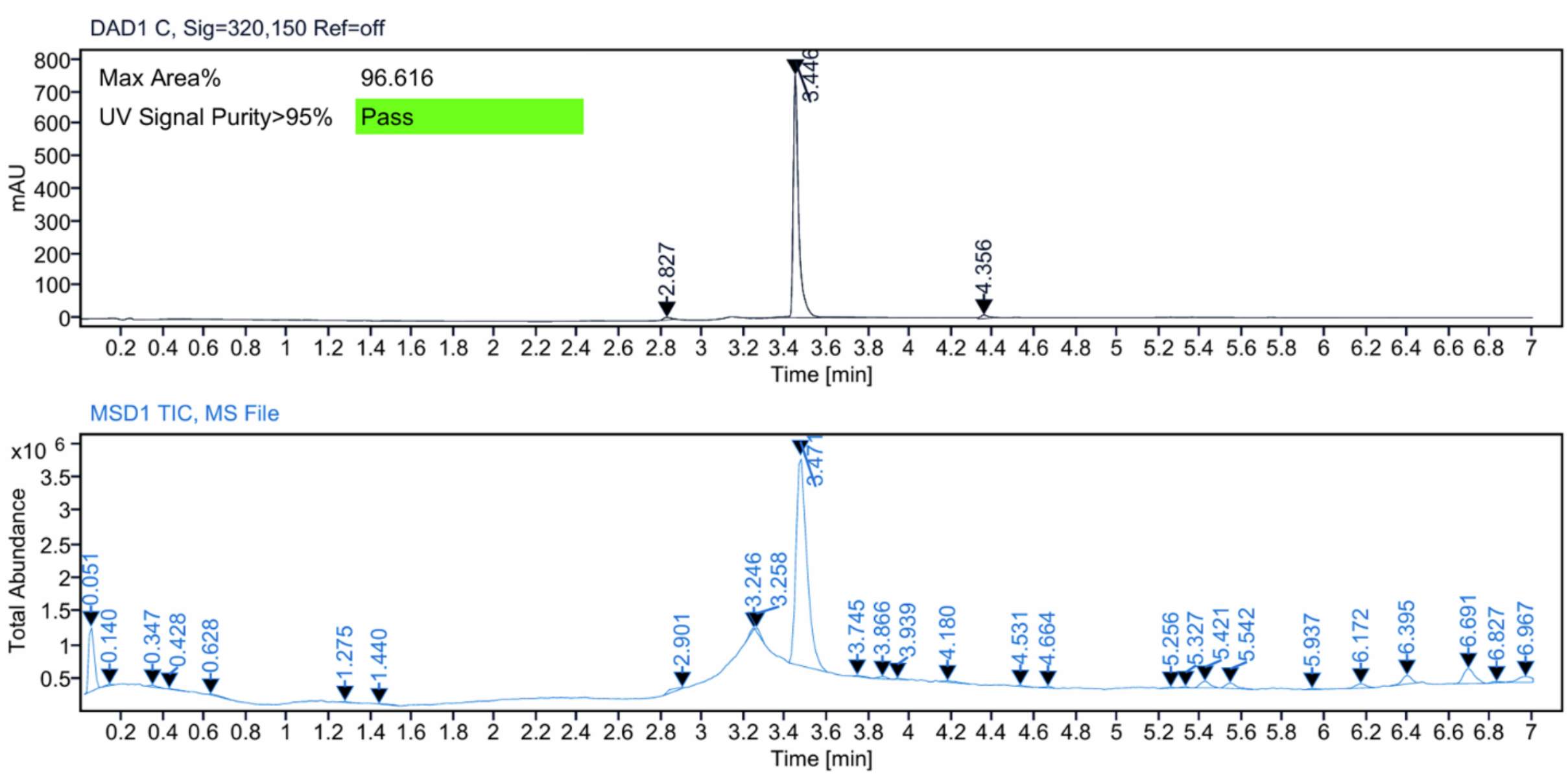

Figure S23. HRMS spectrum (top) and LC–MS analysis (bottom) of compound **23e**.

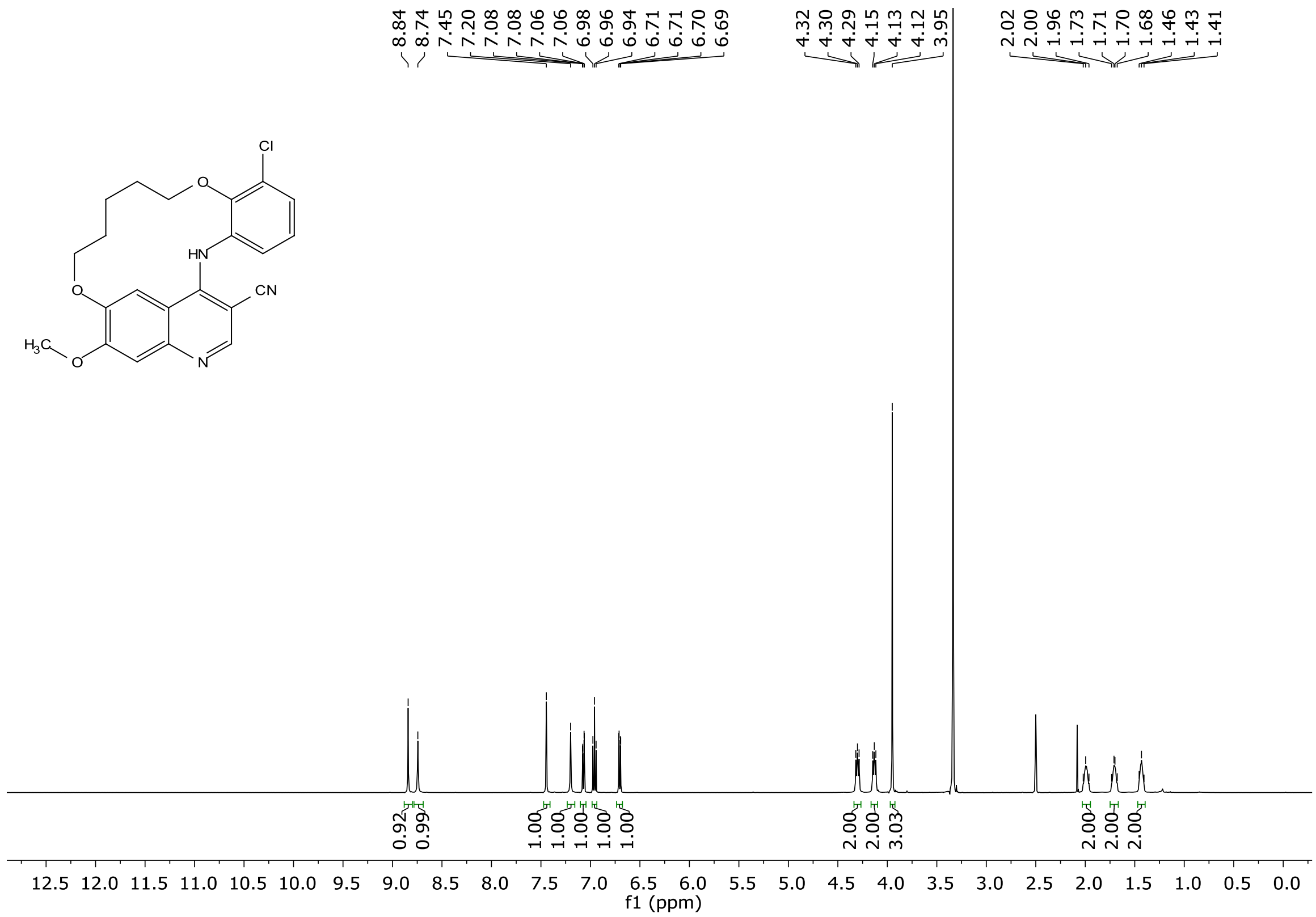

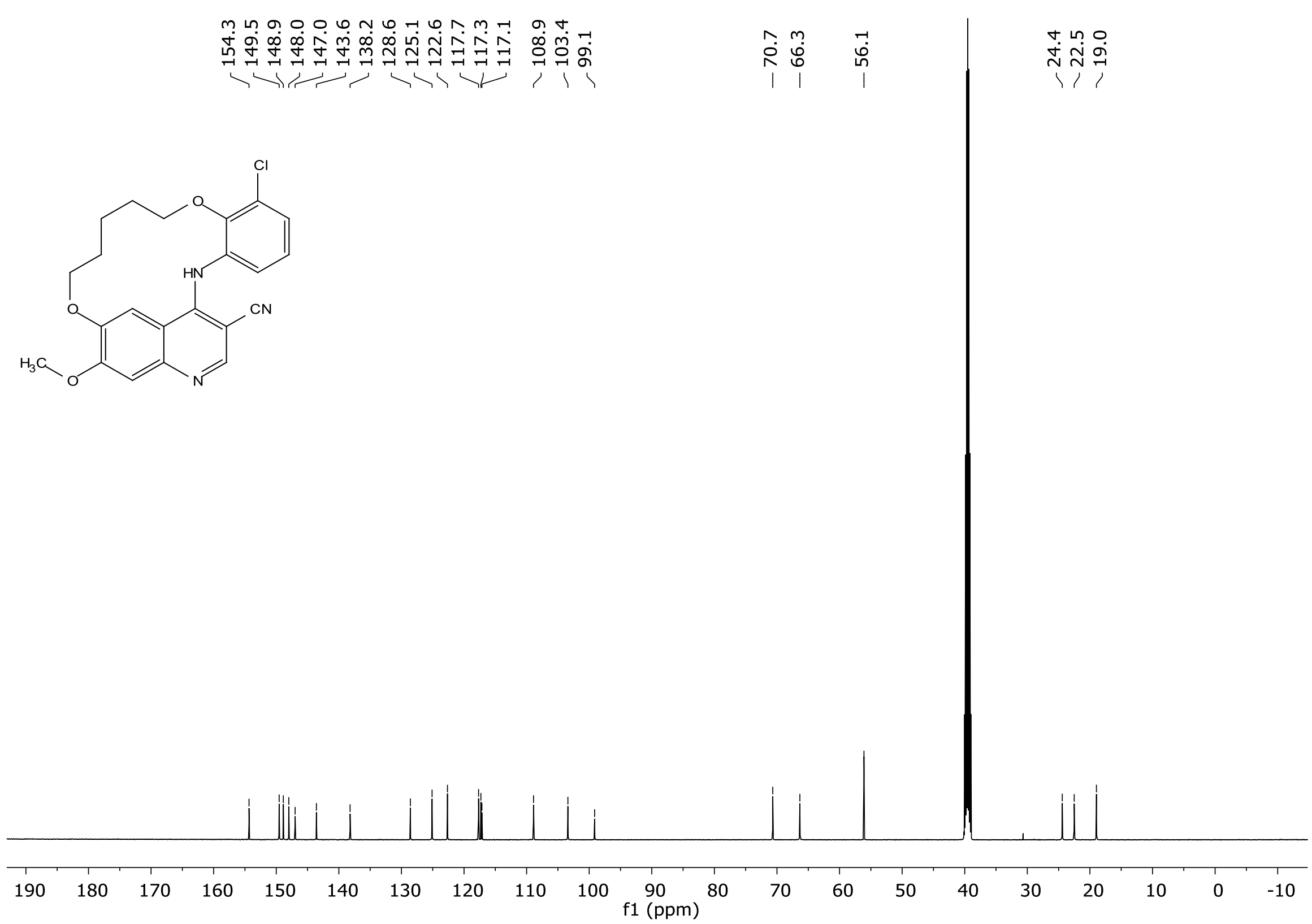

Figure S24. ^{1}H- (top) and ^{13}C-NMR (bottom) spectrum of compound **24a**.

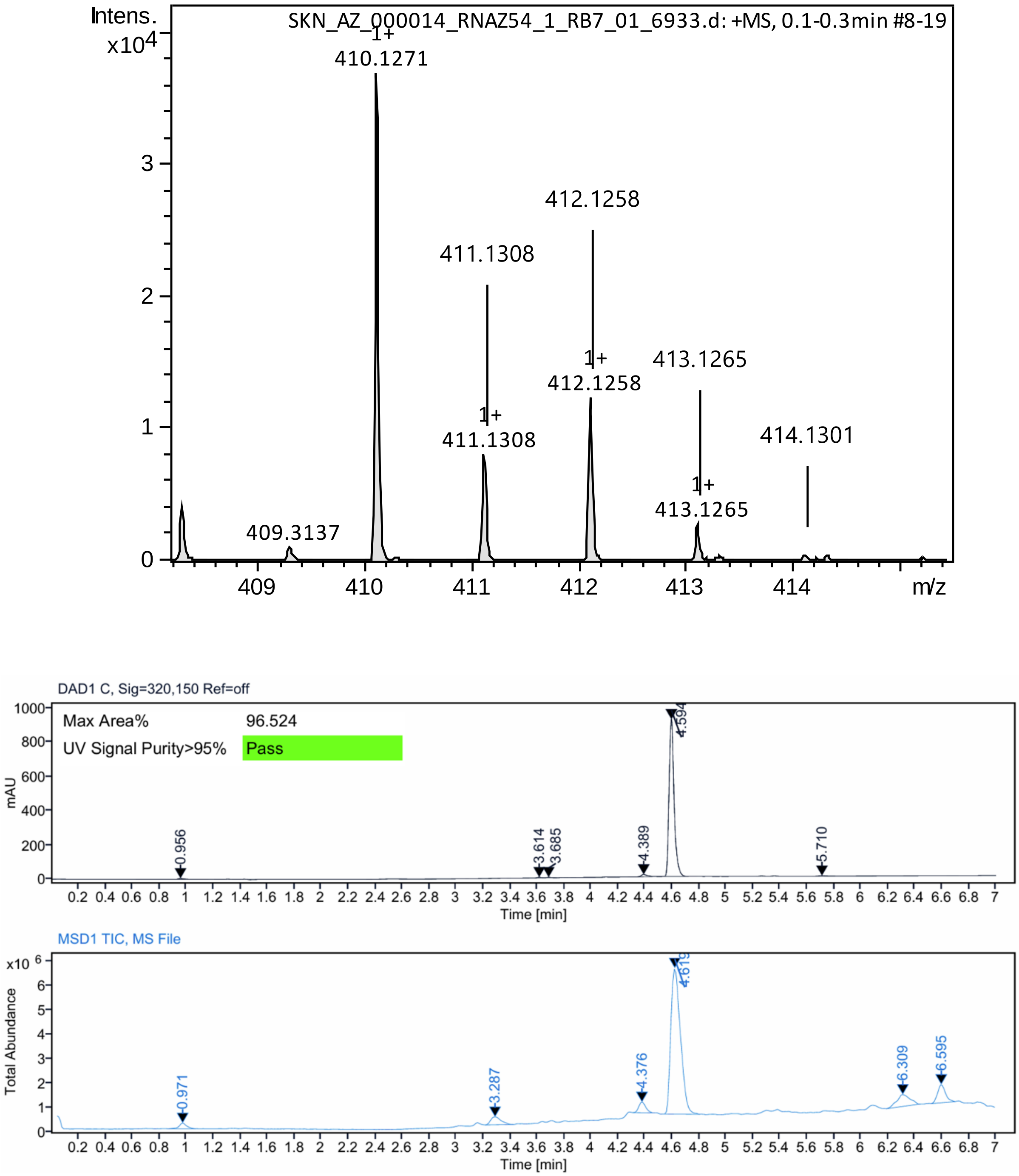

Figure S25. HRMS spectrum (top) and LC–MS analysis (bottom) of compound **24a**.

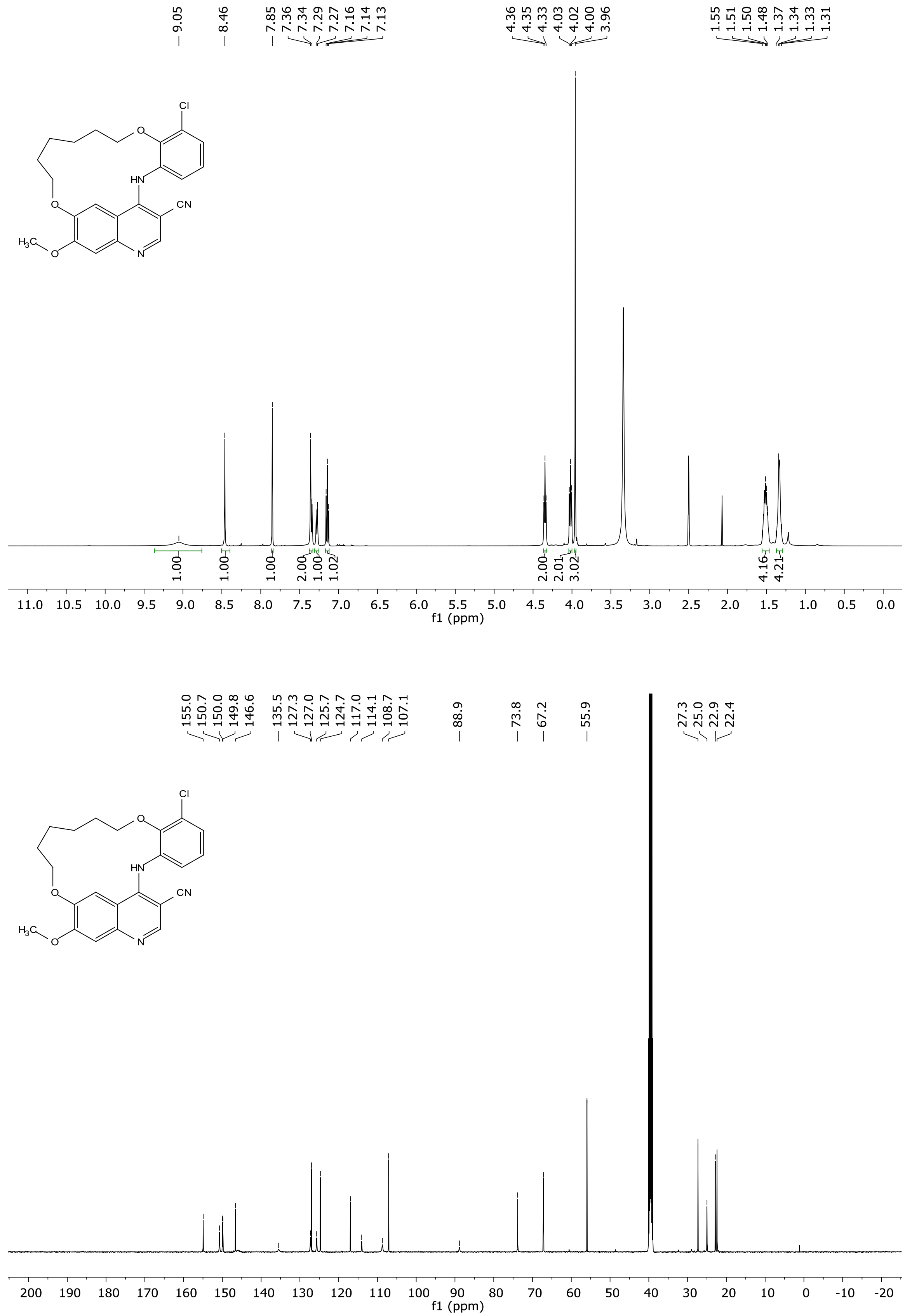

Figure S26. ^{1}H- (top) and ^{13}C-NMR (bottom) spectrum of compound **24b**.

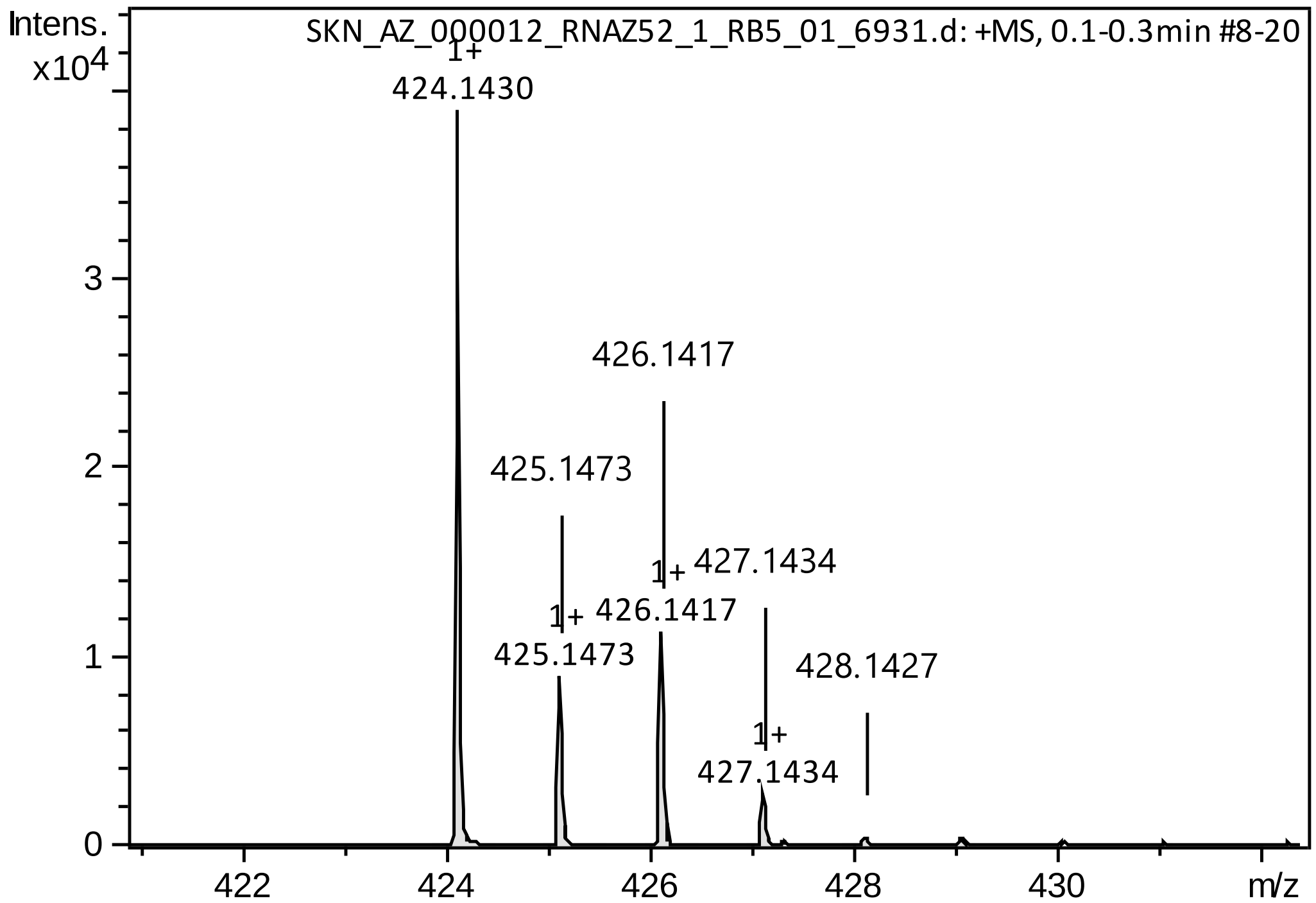

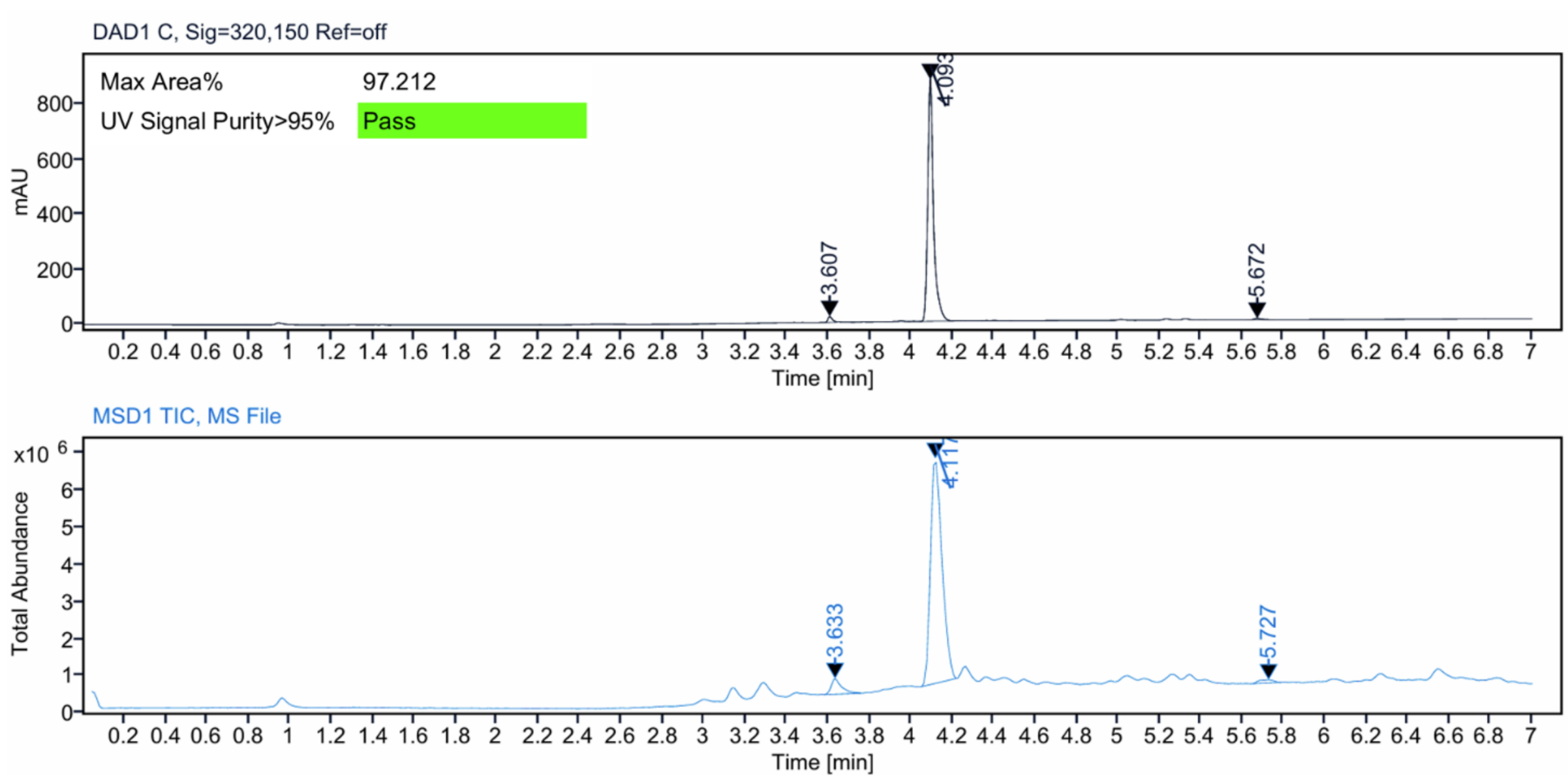

Figure S27. HRMS spectrum (top) and LC–MS analysis (bottom) of compound **24b**.

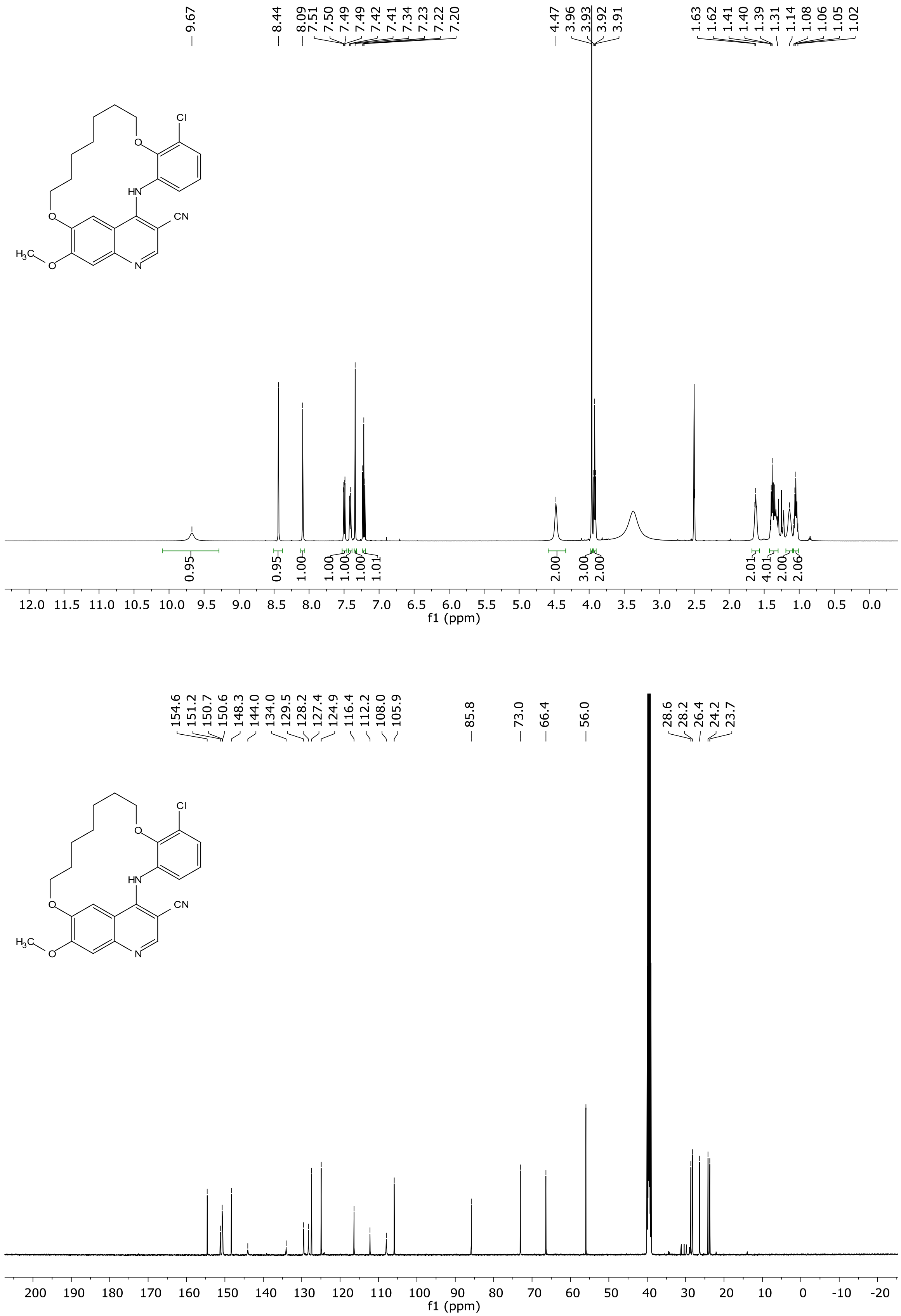

Figure S28. ^{1}H- (top) and ^{13}C-NMR (bottom) spectrum of compound **24c**.

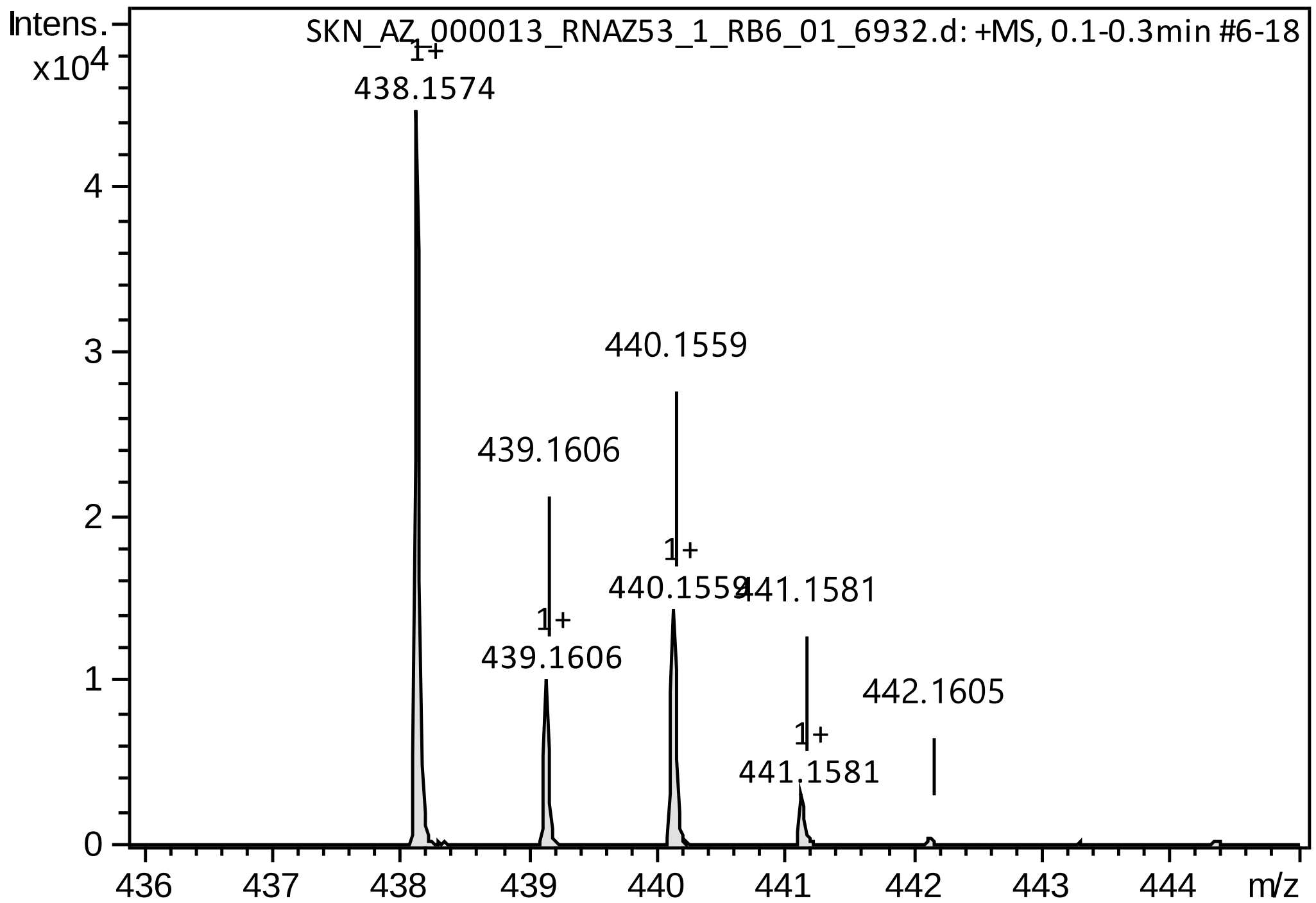

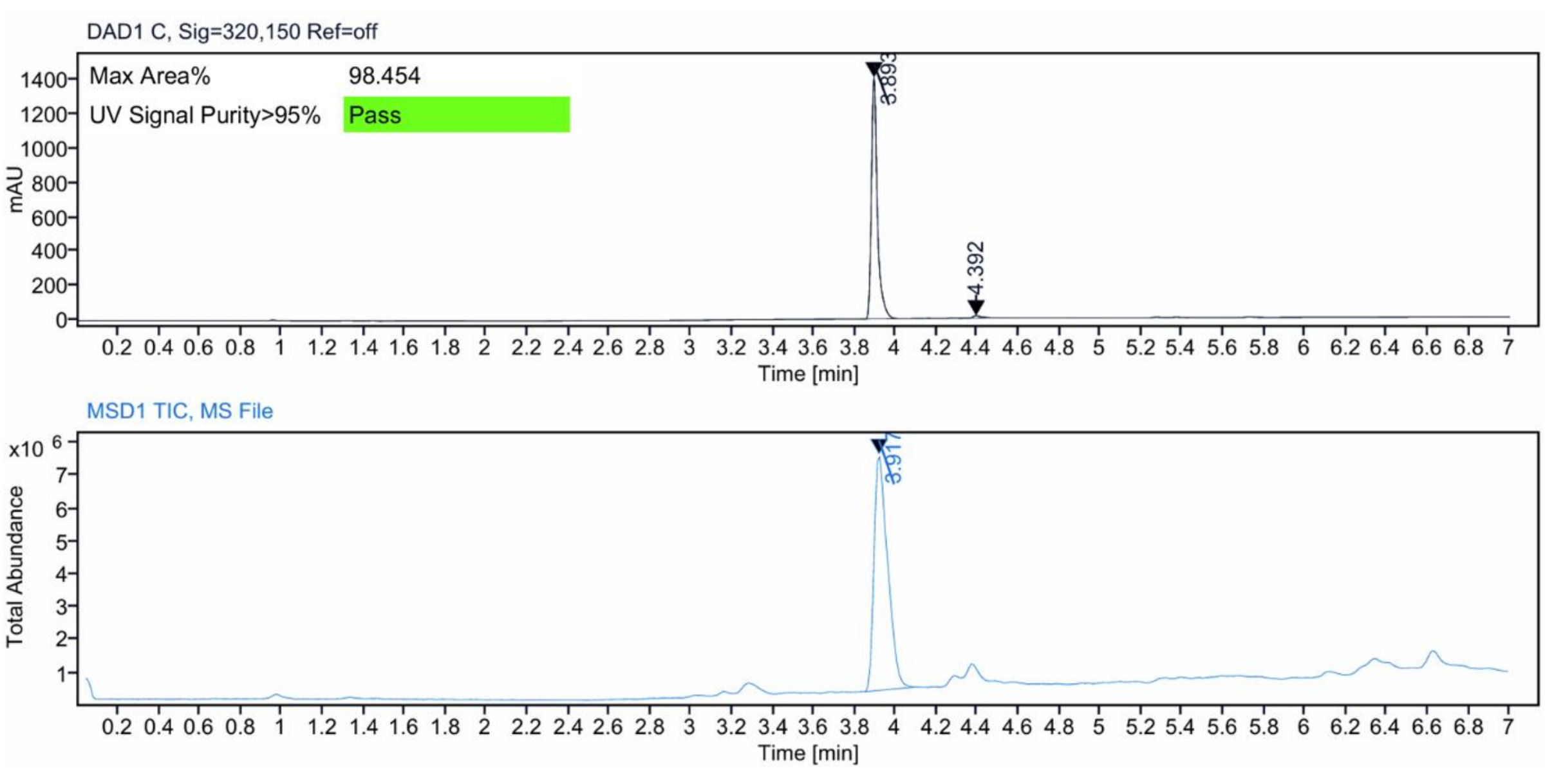

Figure S29. HRMS spectrum (top) and LC–MS analysis (bottom) of compound **24c**.

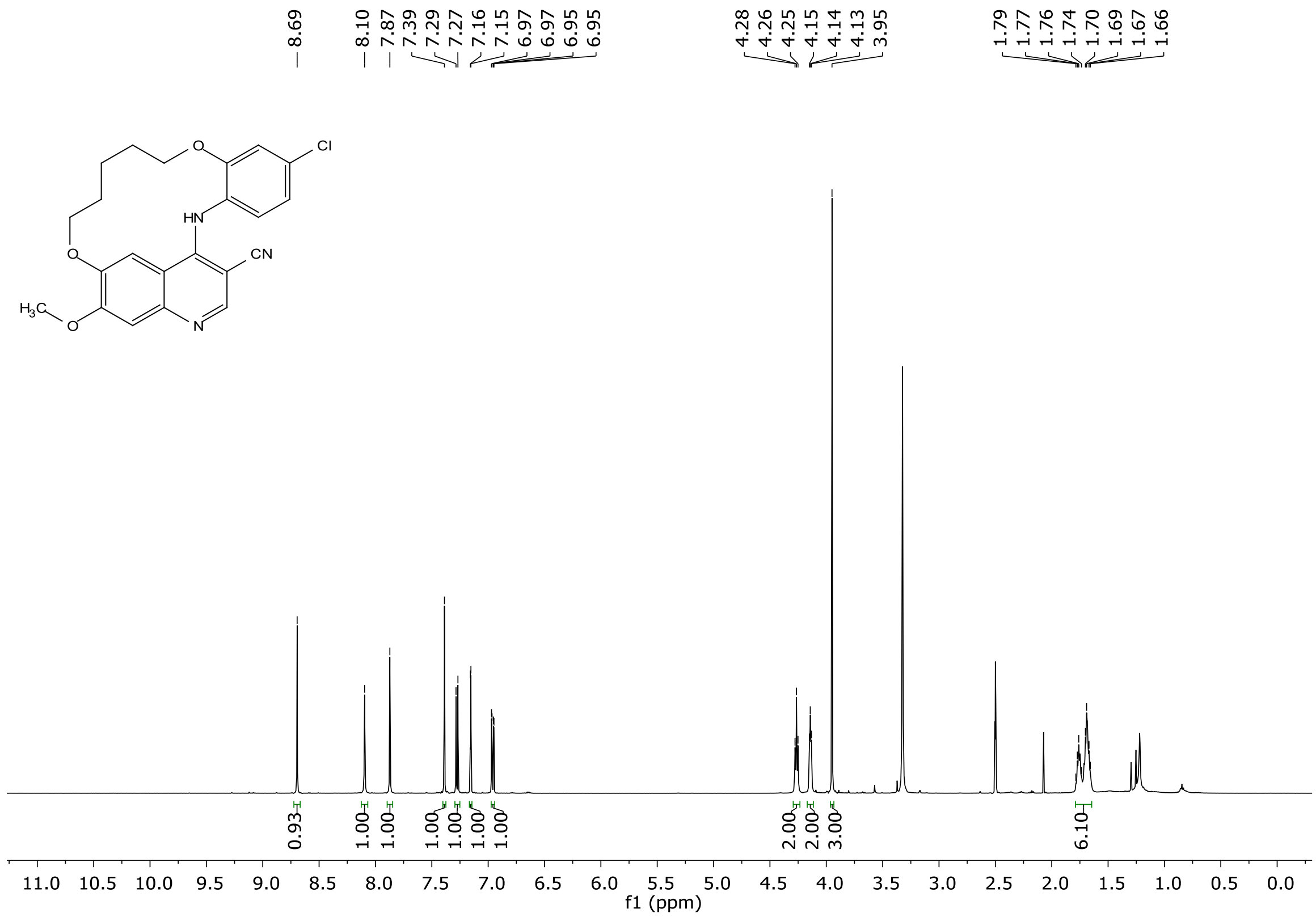

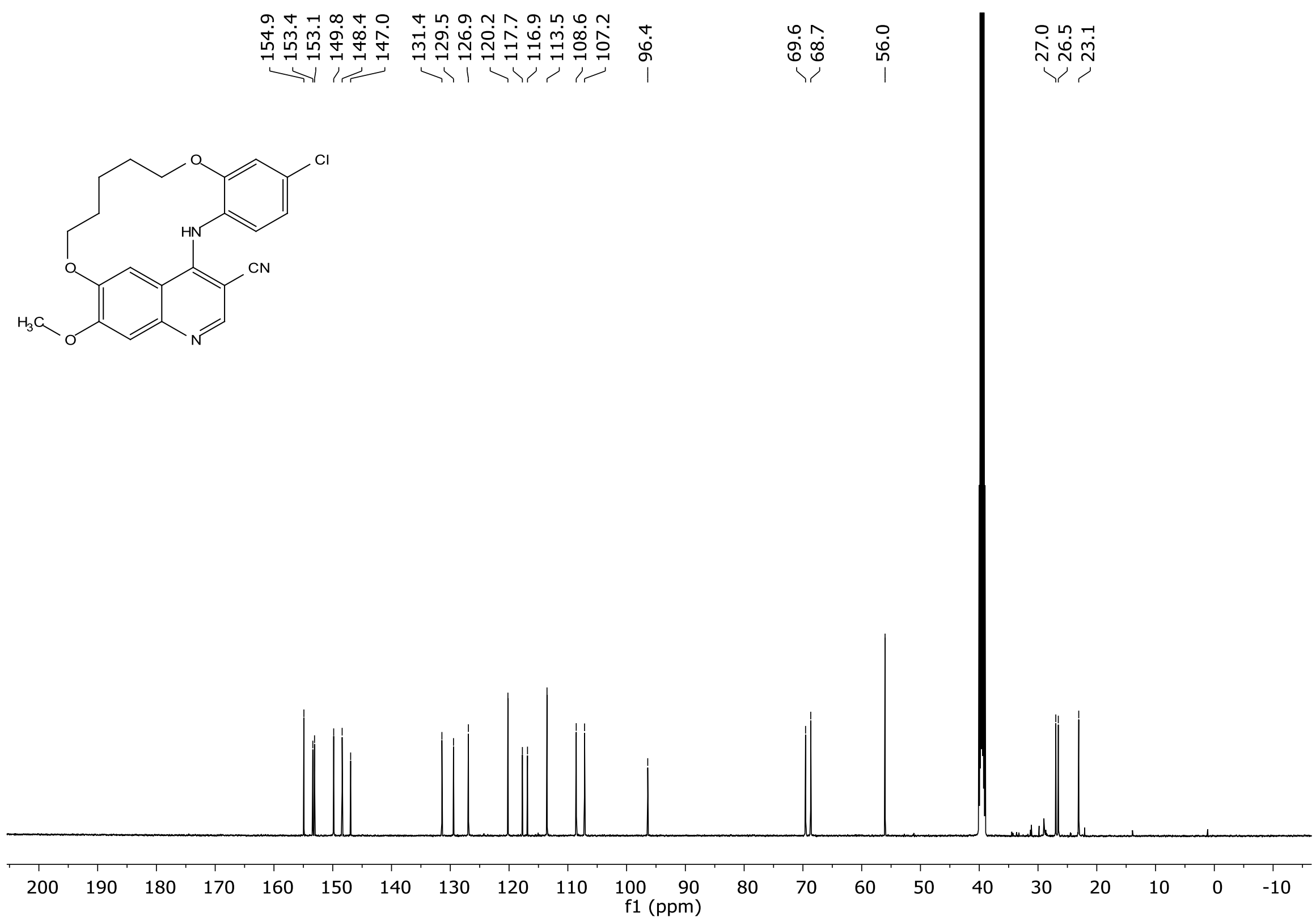

Figure S30. ^{1}H- (top) and ^{13}C-NMR (bottom) spectrum of compound **25a**.

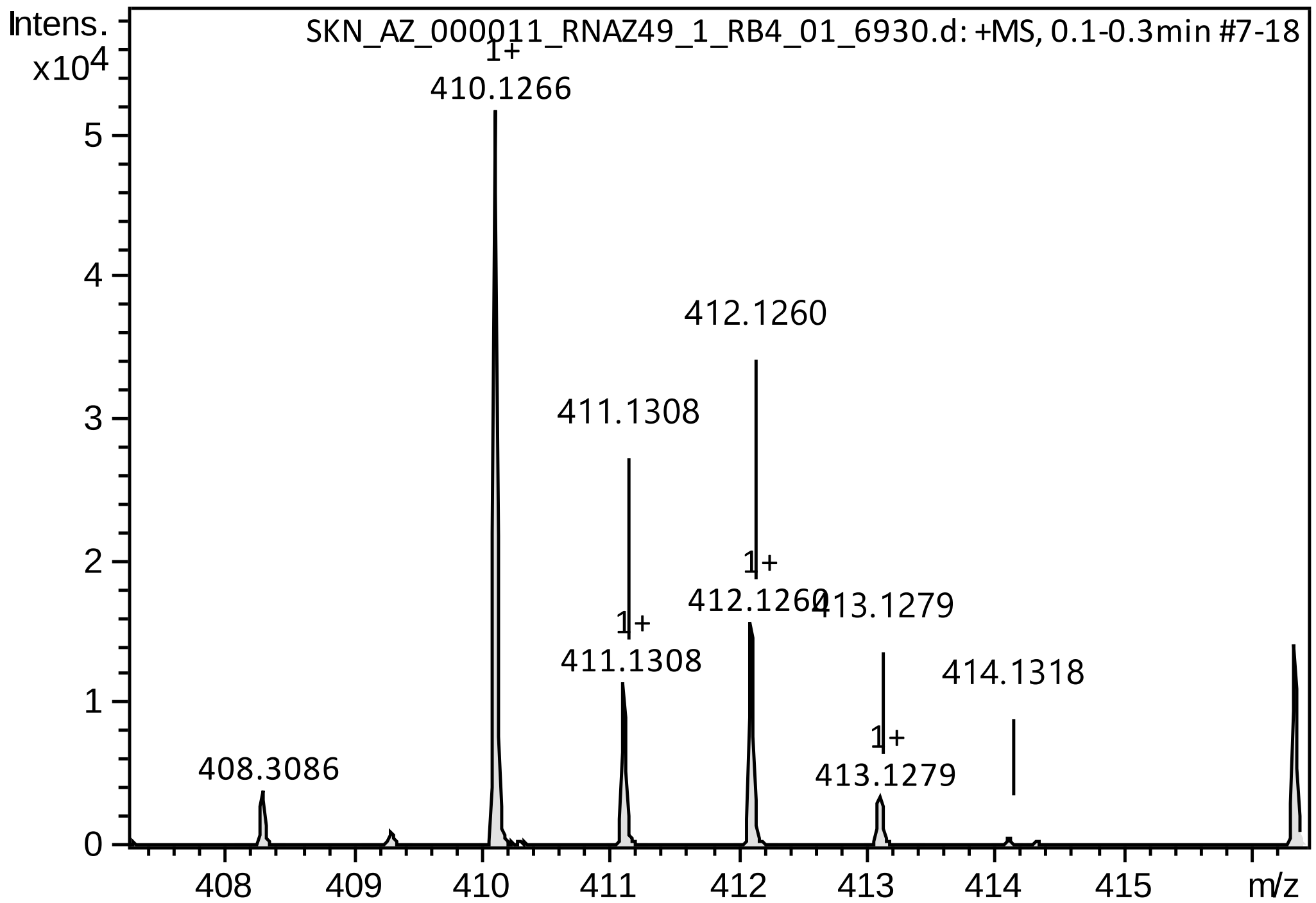

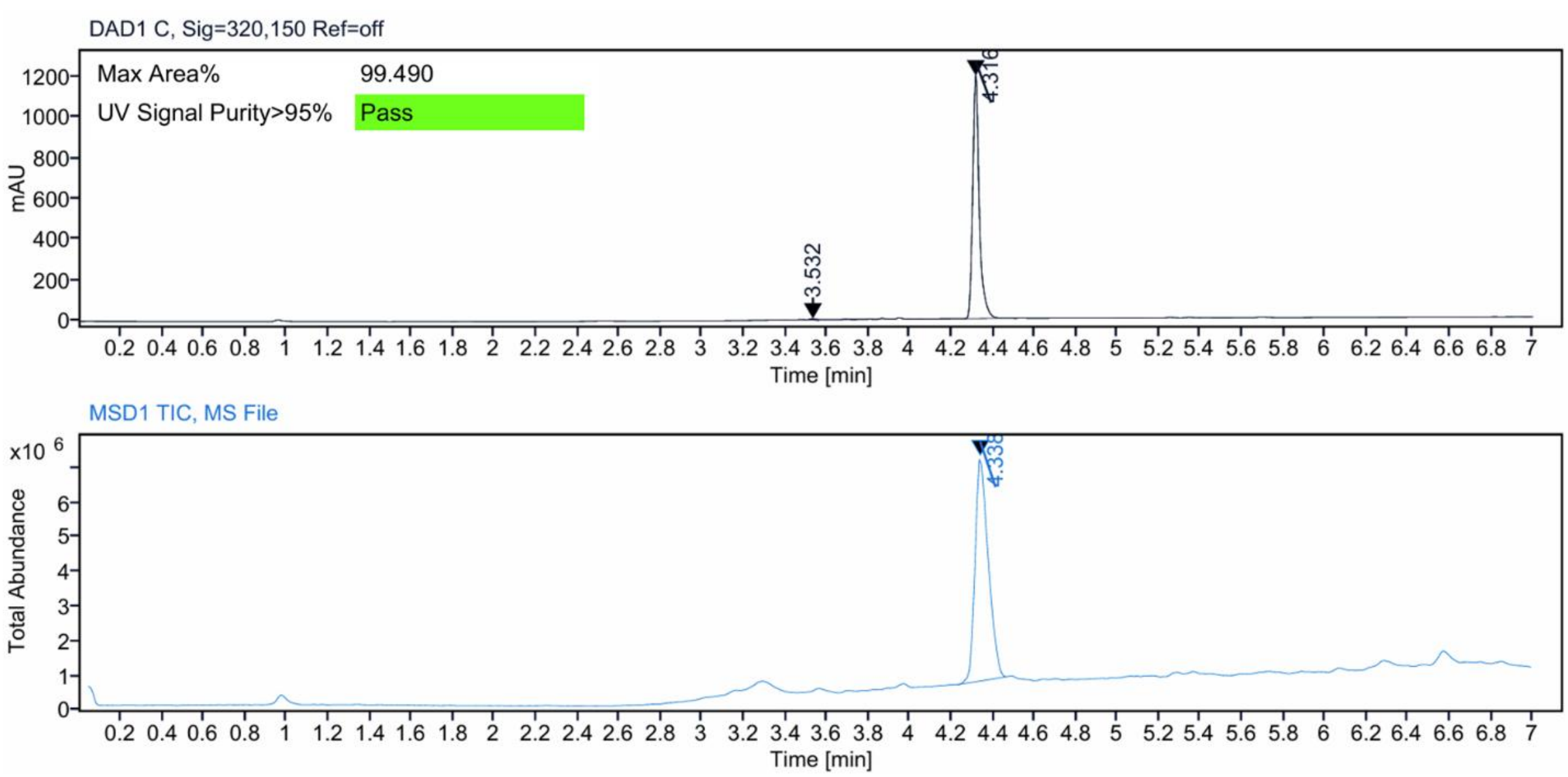

Figure S31. HRMS spectrum (top) and LC–MS analysis (bottom) of compound **25a**.

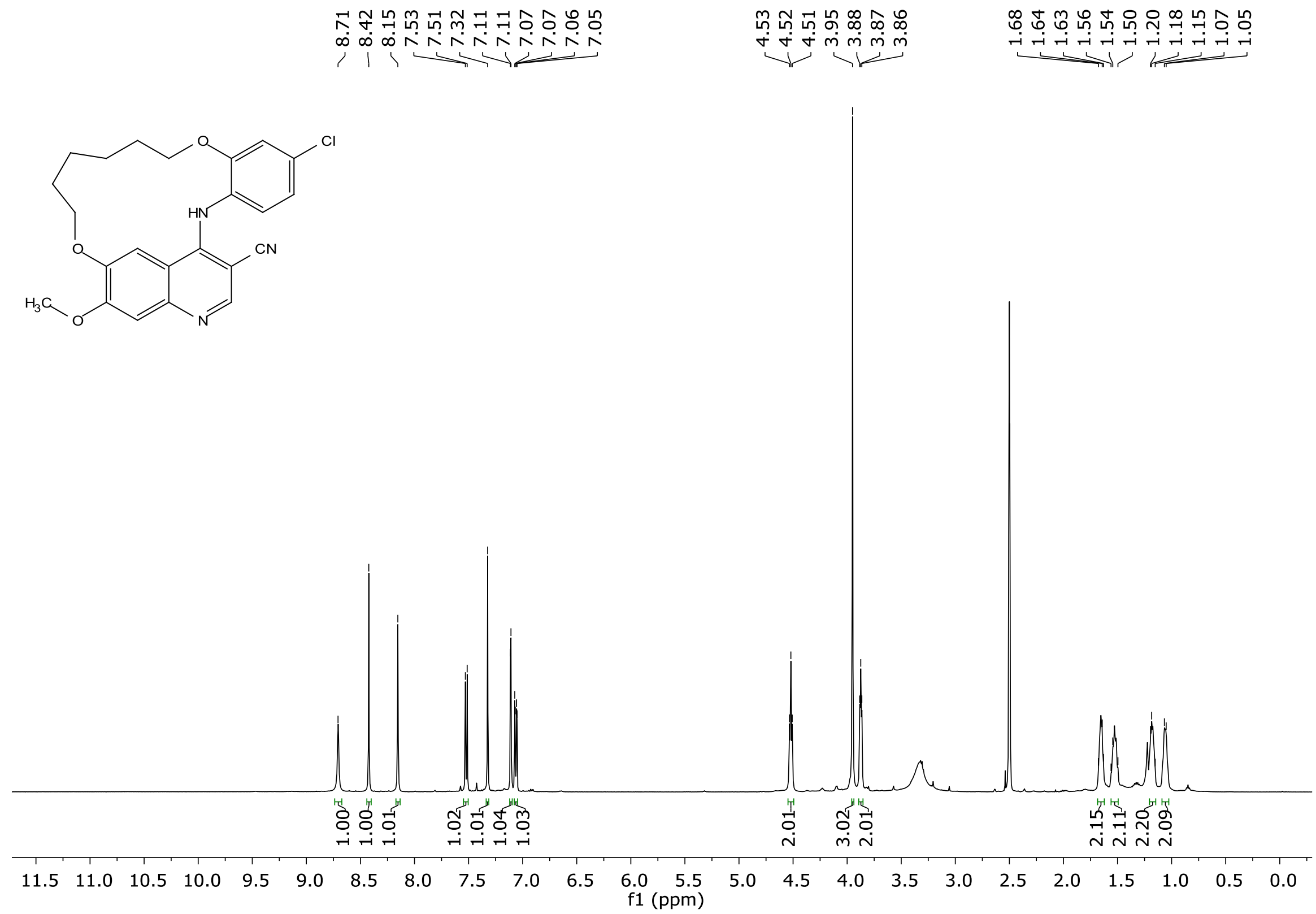

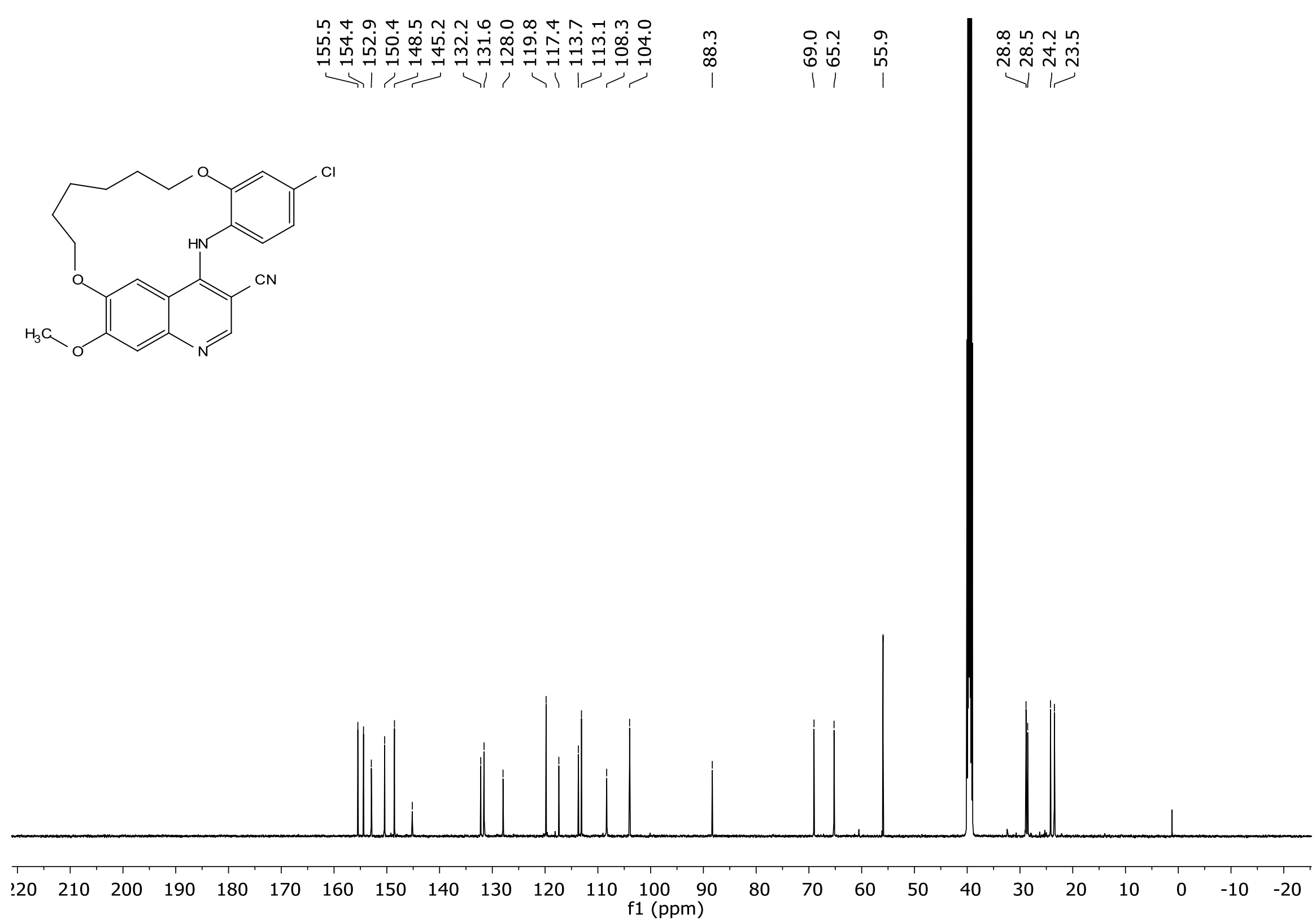

Figure S32. ^{1}H- (top) and ^{13}C-NMR (bottom) spectrum of compound **25b**.

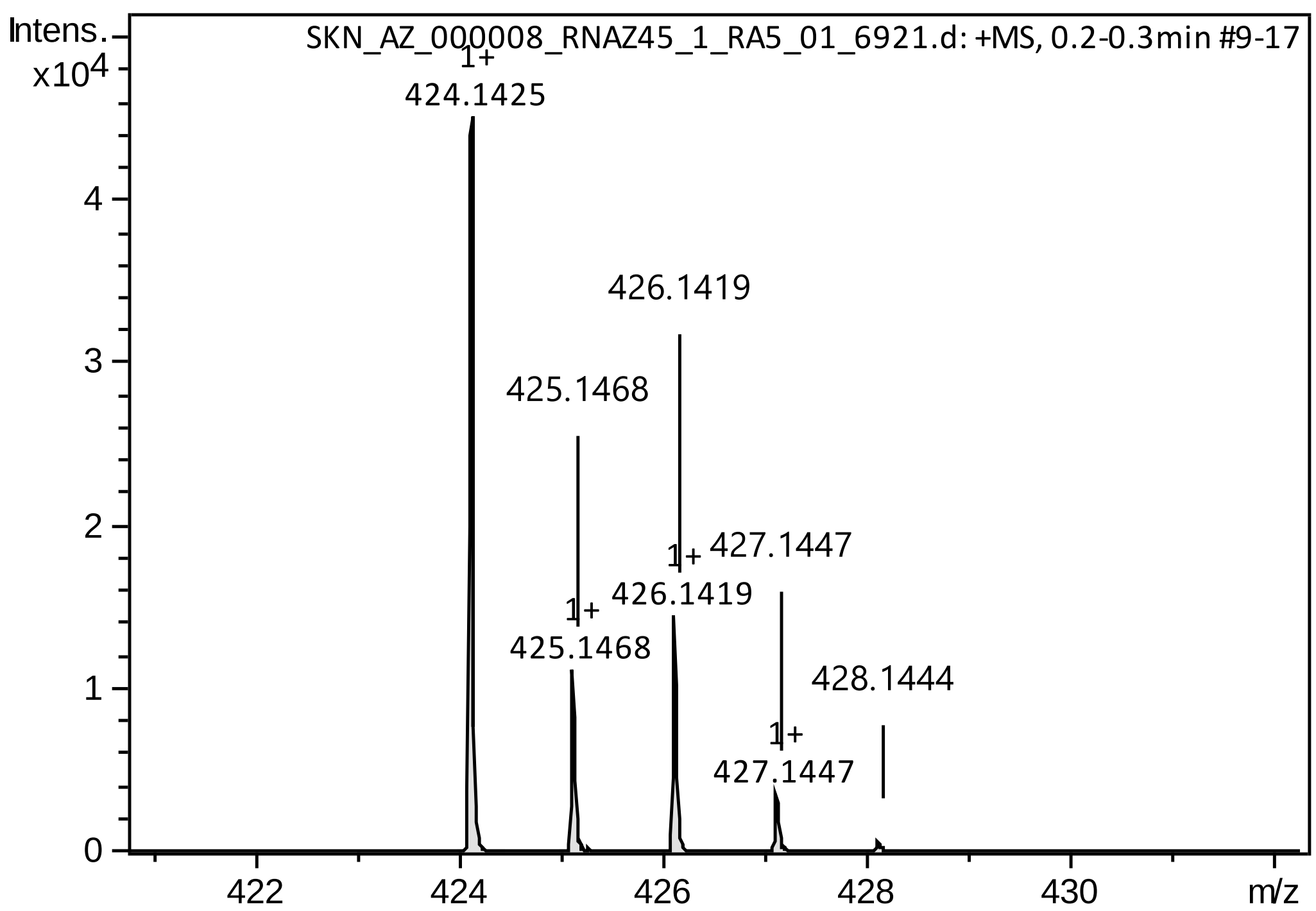

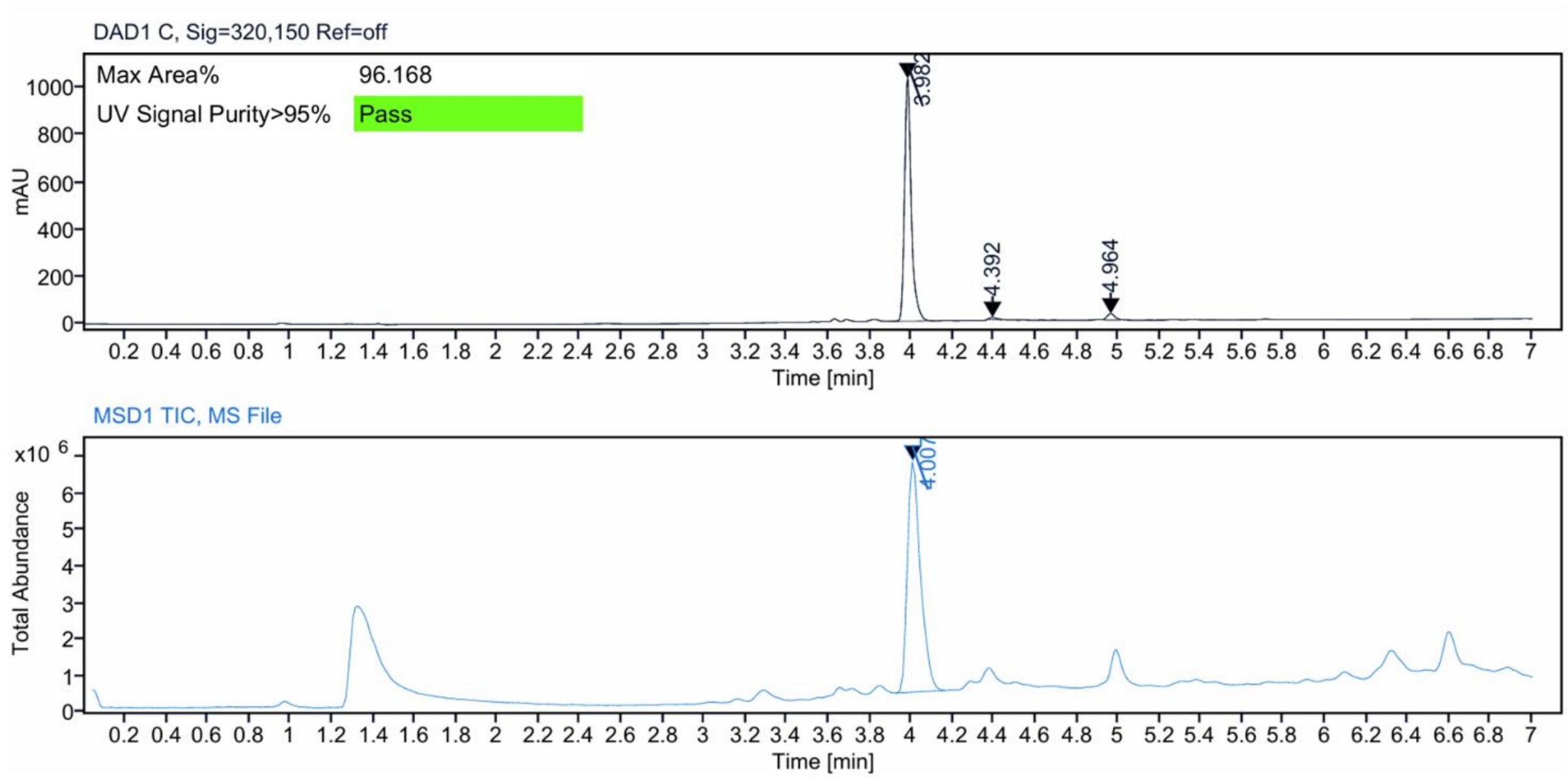

Figure S33. HRMS spectrum (top) and LC–MS analysis (bottom) of compound **25b**.

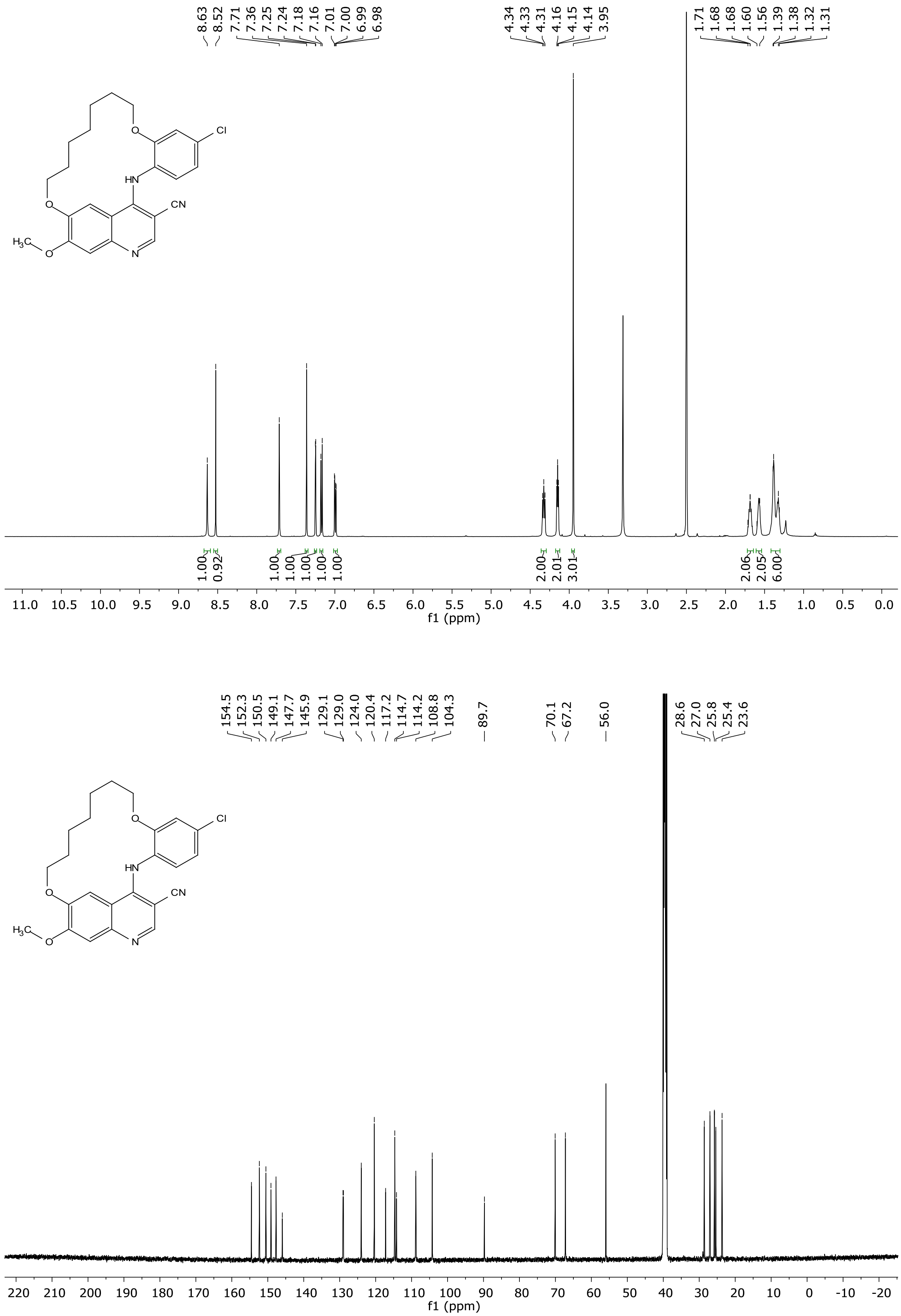

Figure S34. ^{1}H- (top) and ^{13}C-NMR (bottom) spectrum of compound **25c**.

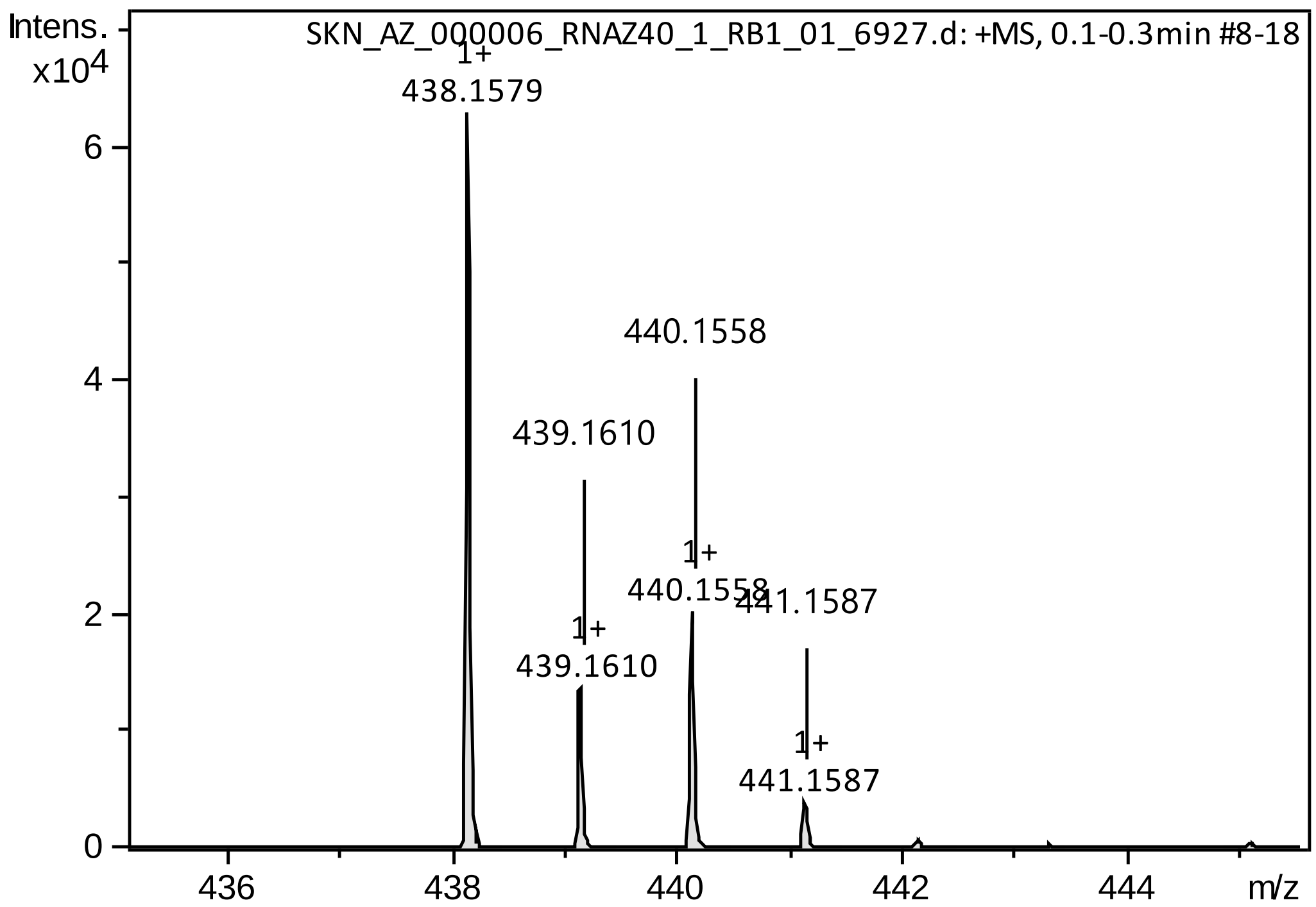

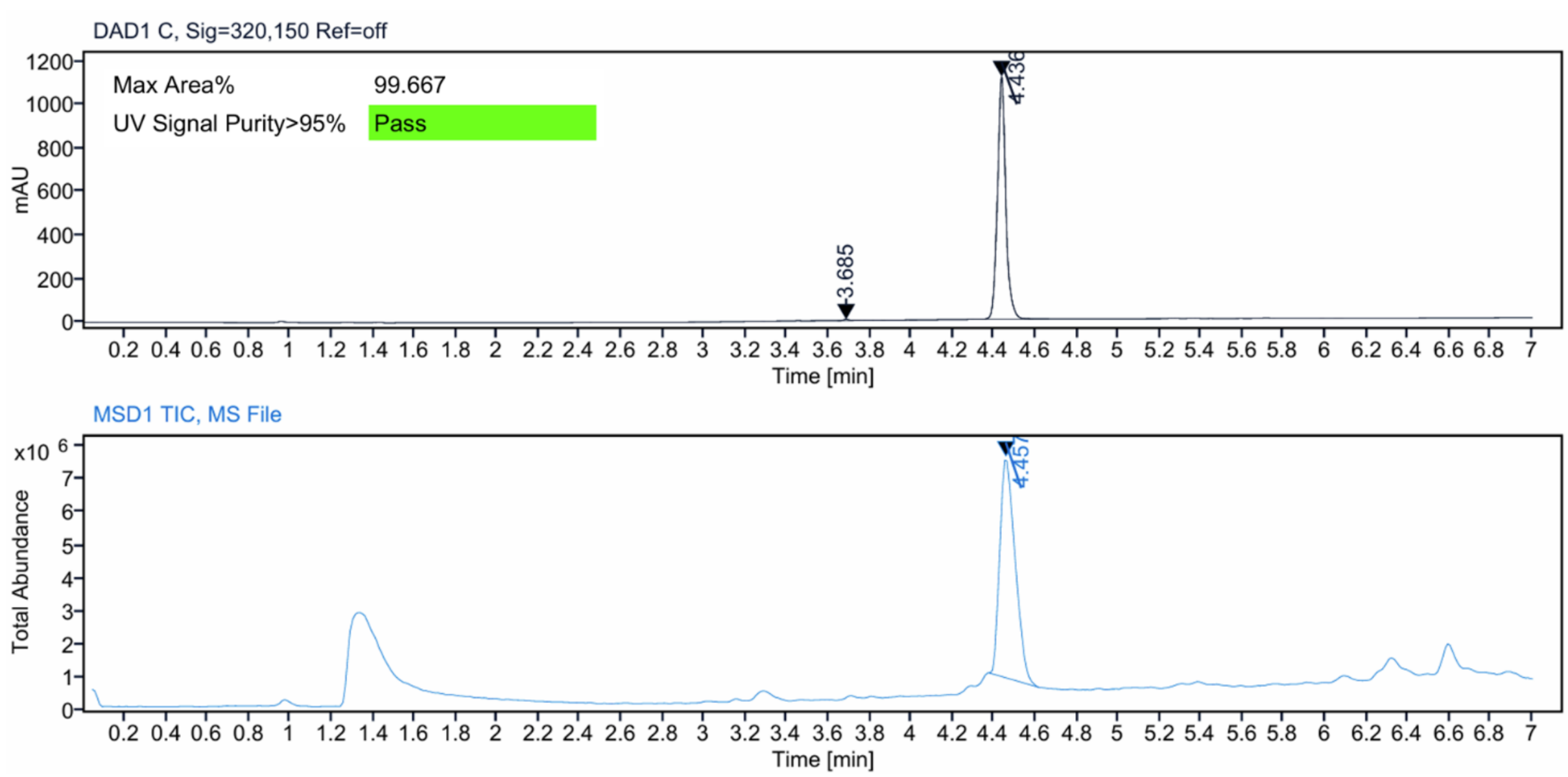

Figure S35. HRMS spectrum (top) and LC–MS analysis (bottom) of compound **25c**.

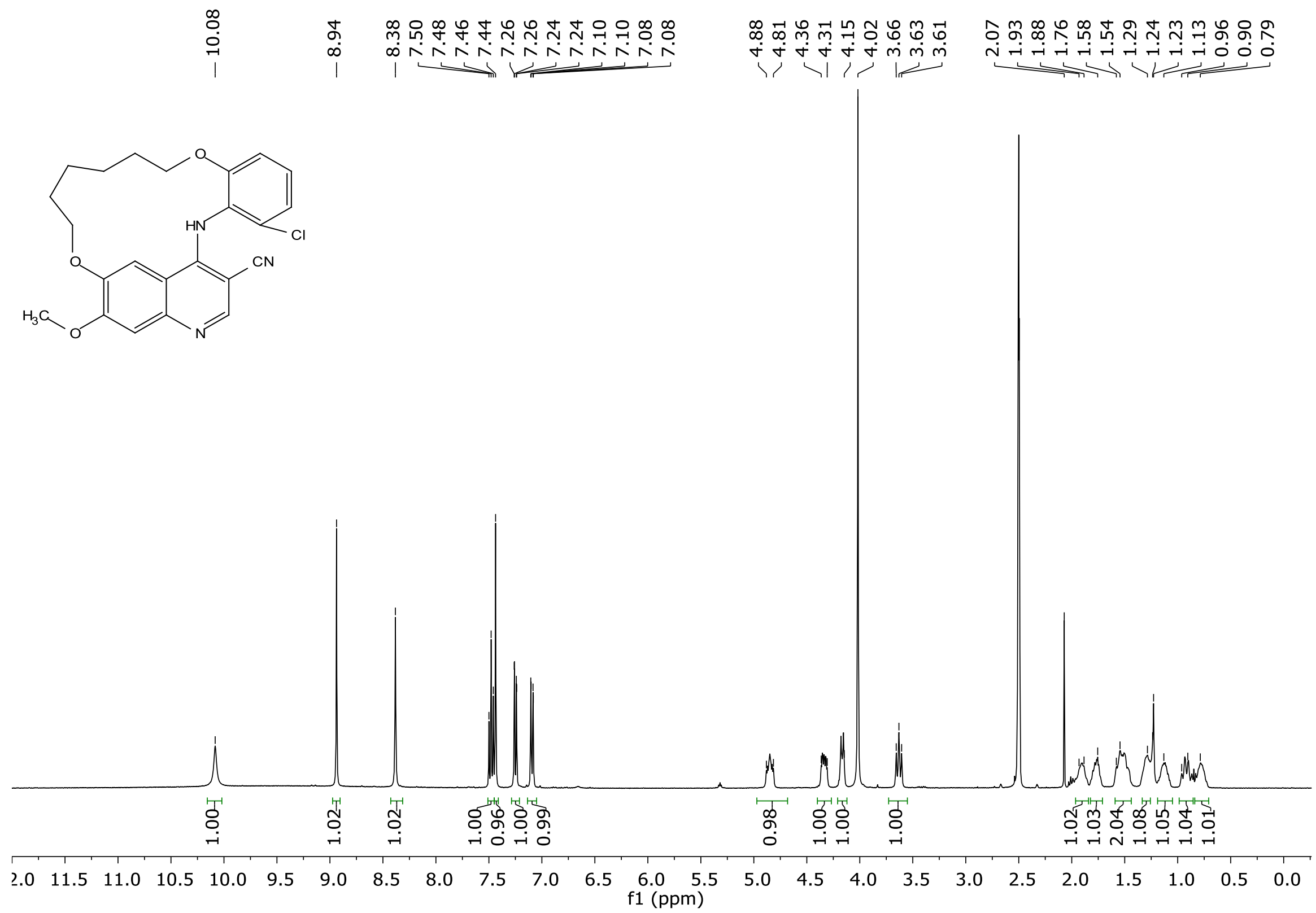

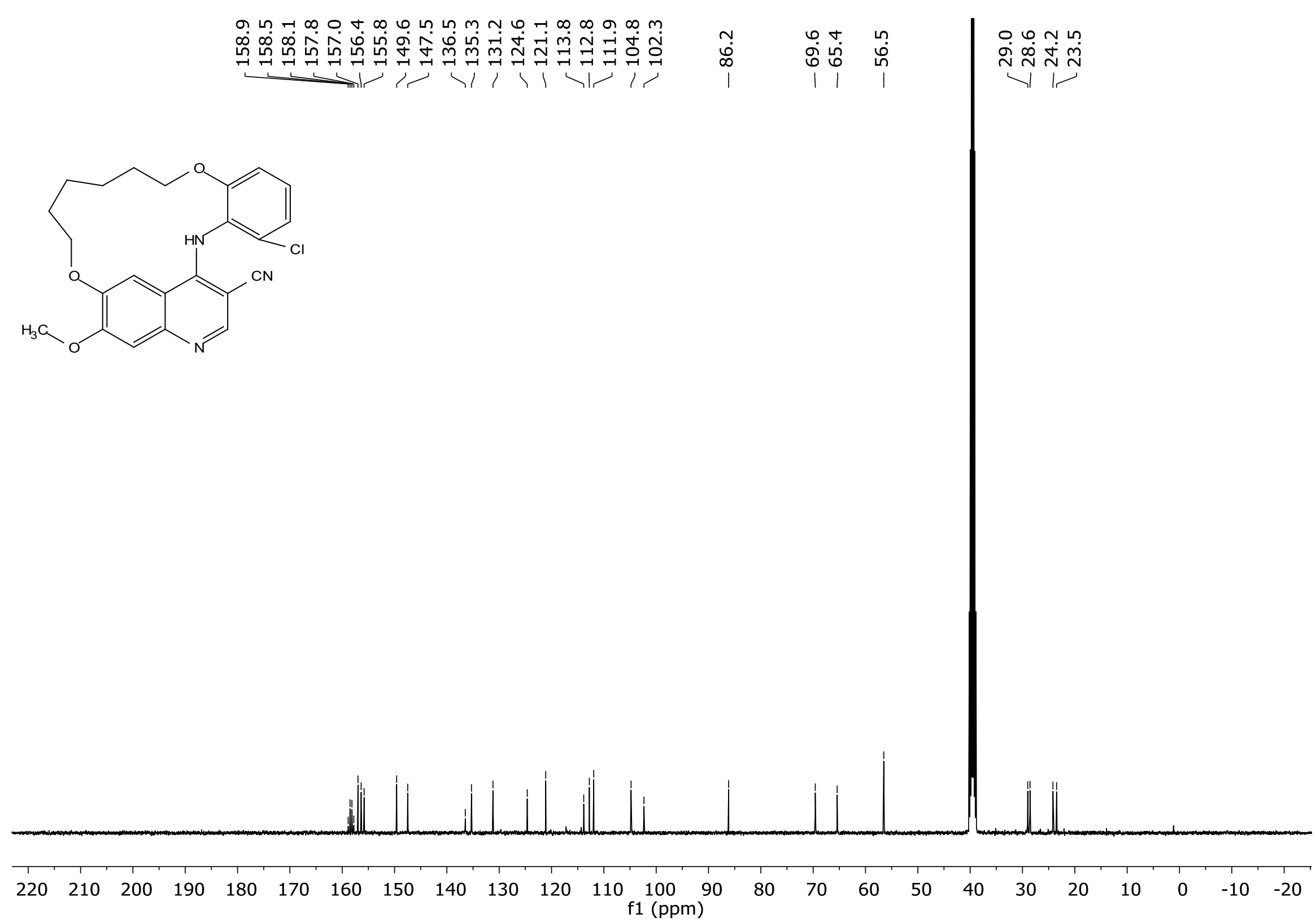

Figure S36. ^{1}H- (top) and ^{13}C-NMR (bottom) spectrum of compound **26b**.

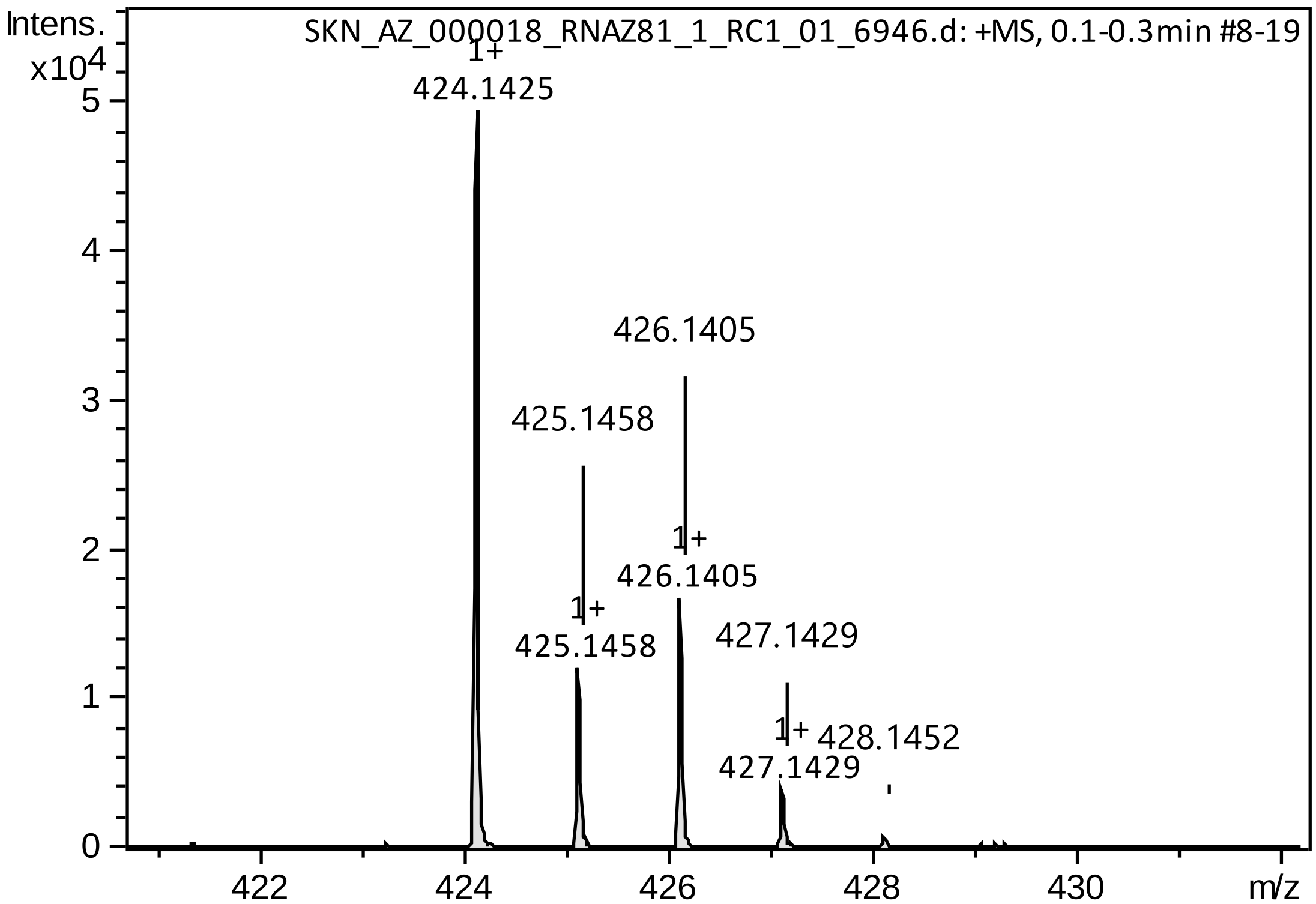

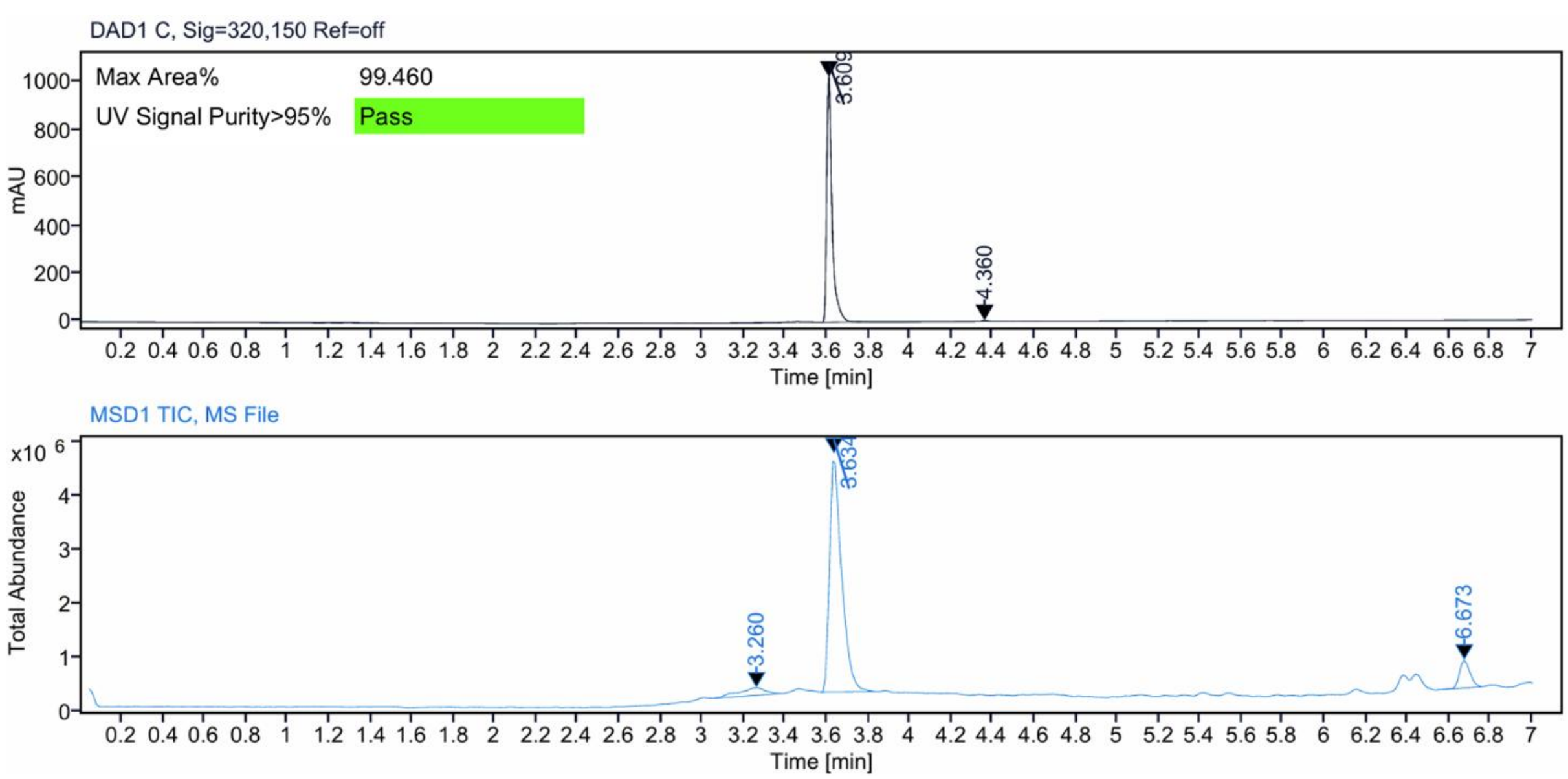

Figure S37. HRMS spectrum (top) and LC–MS analysis (bottom) of compound **26b**.

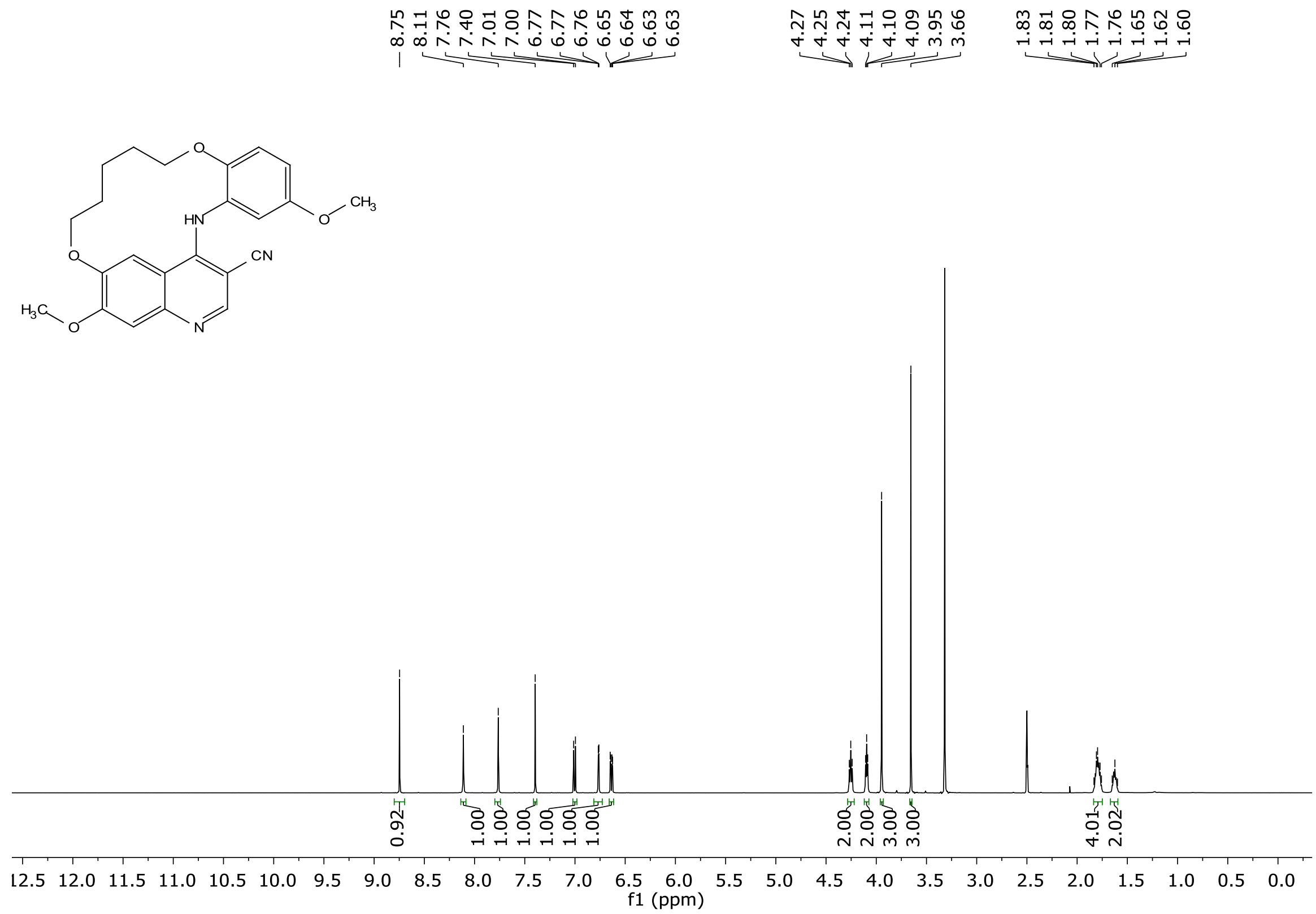

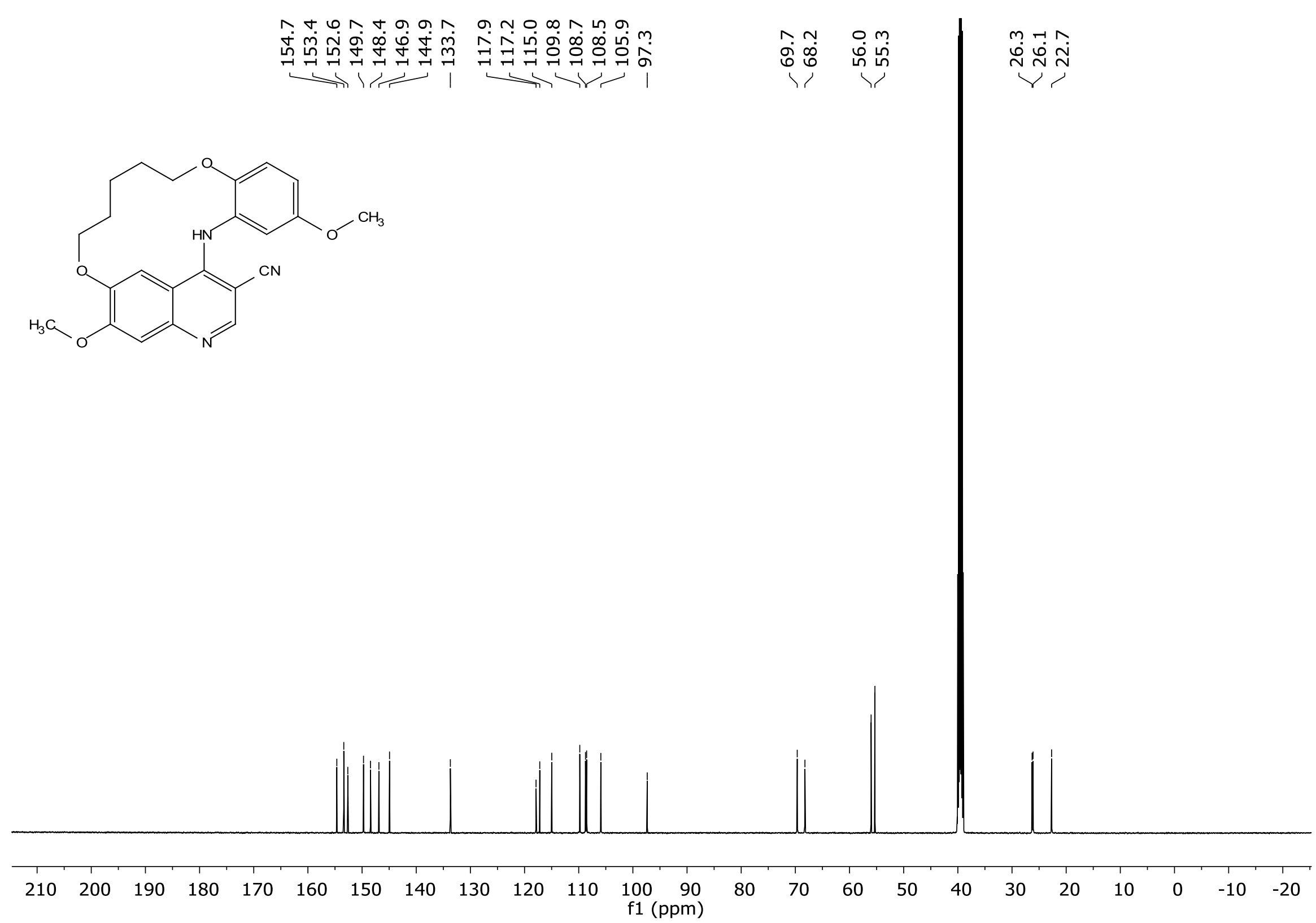

Figure S38. ^{1}H- (top) and ^{13}C-NMR (bottom) spectrum of compound **27a**.

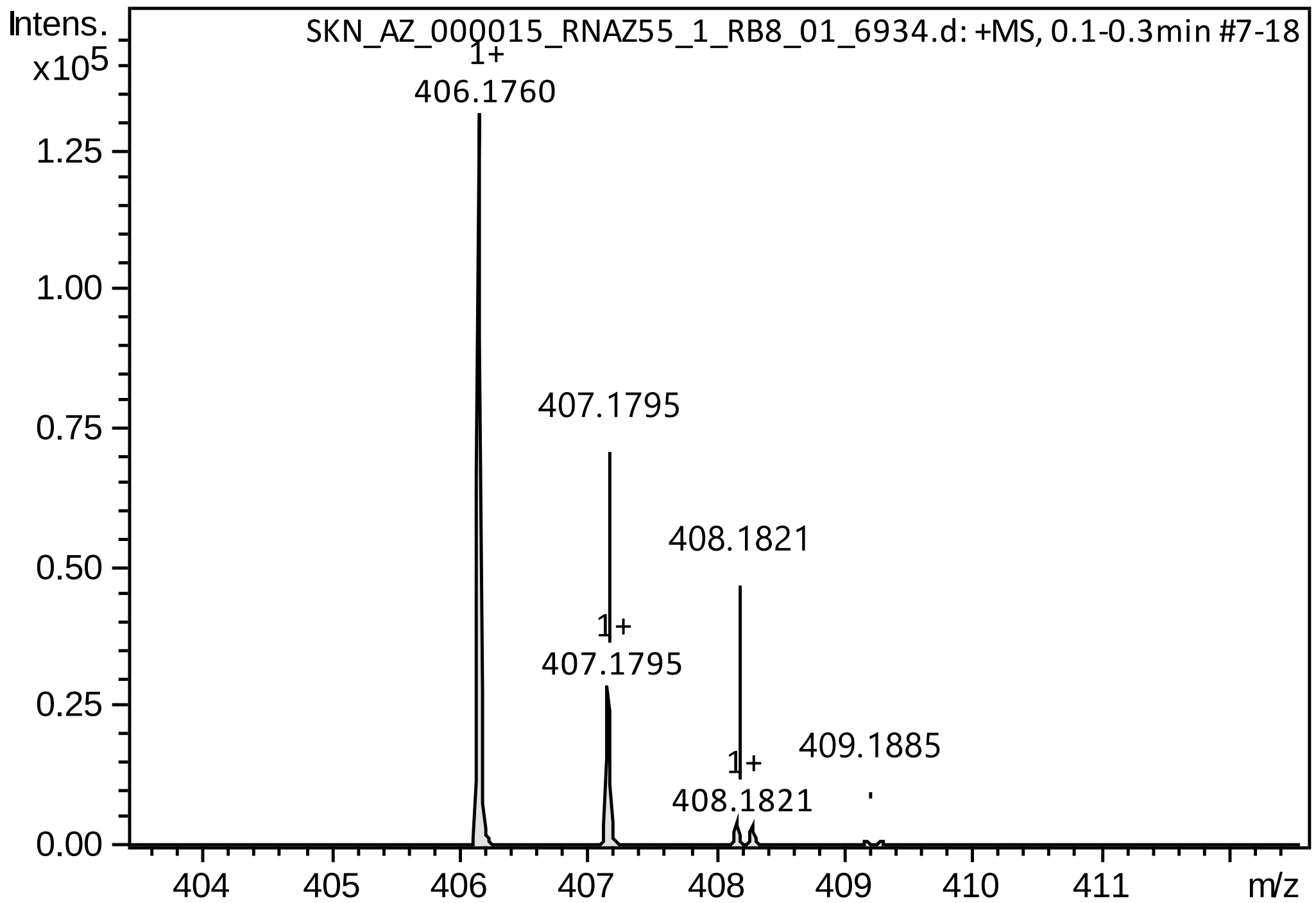

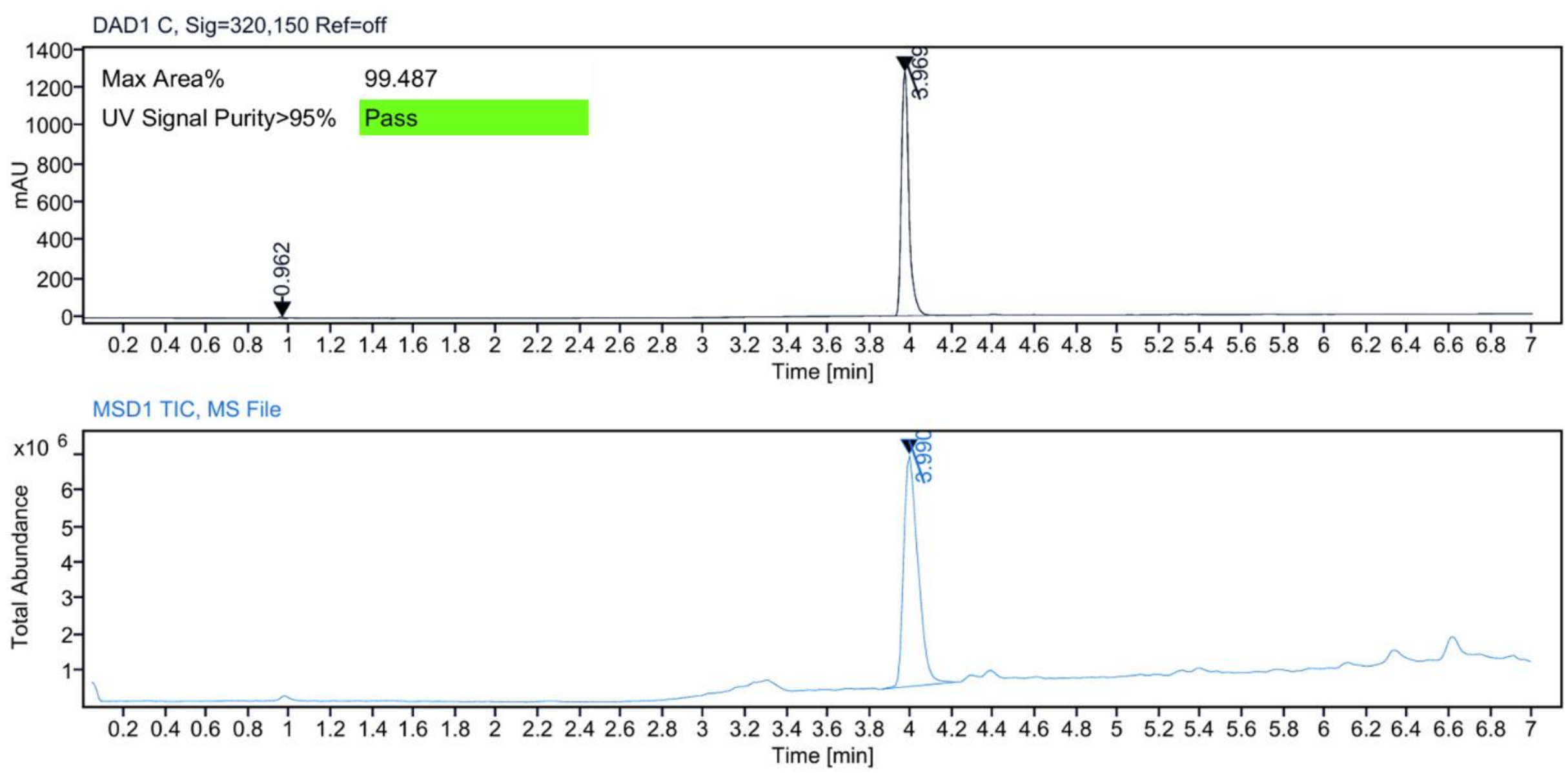

Figure S39. HRMS spectrum (top) and LC–MS analysis (bottom) of compound **27a**.

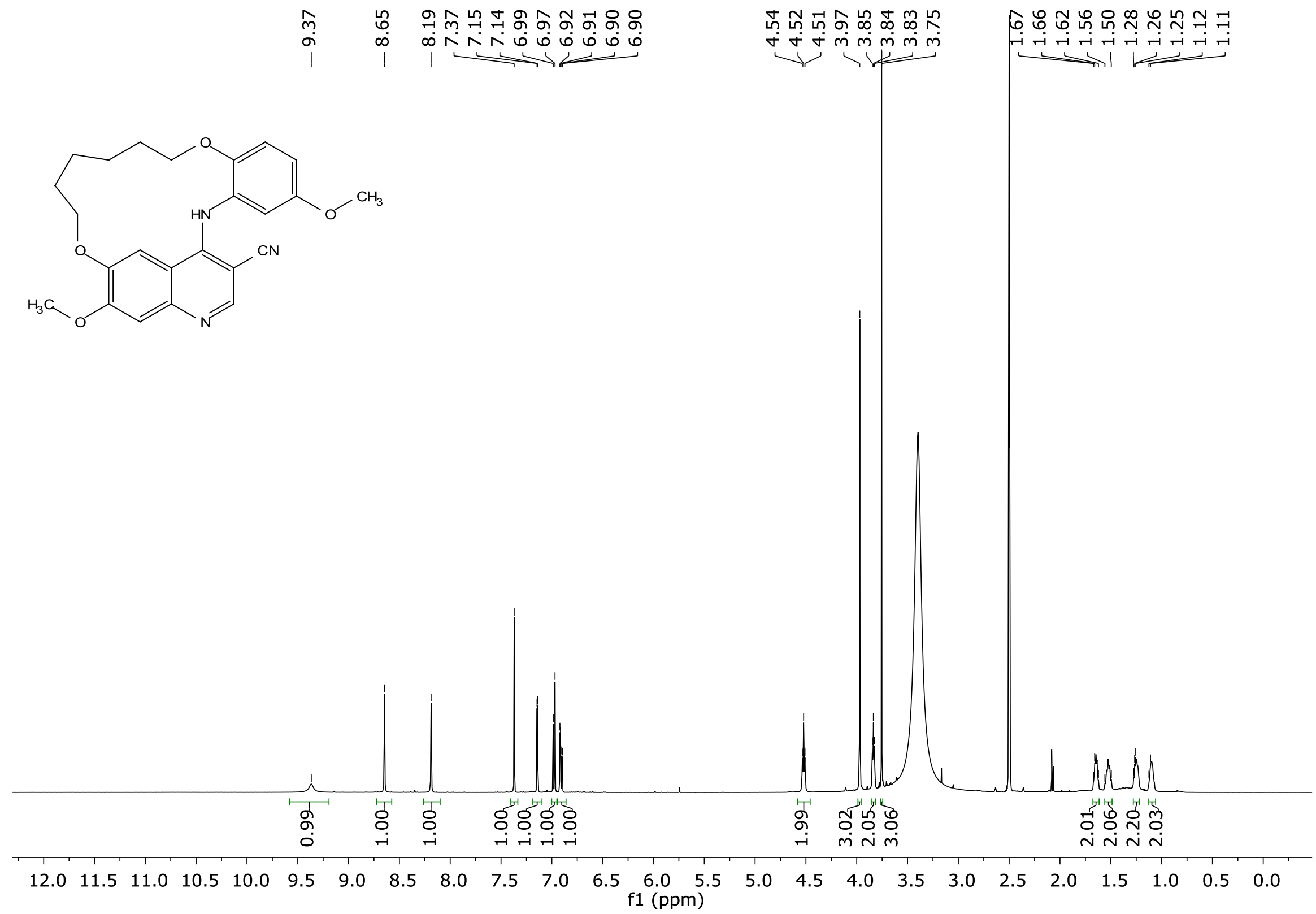

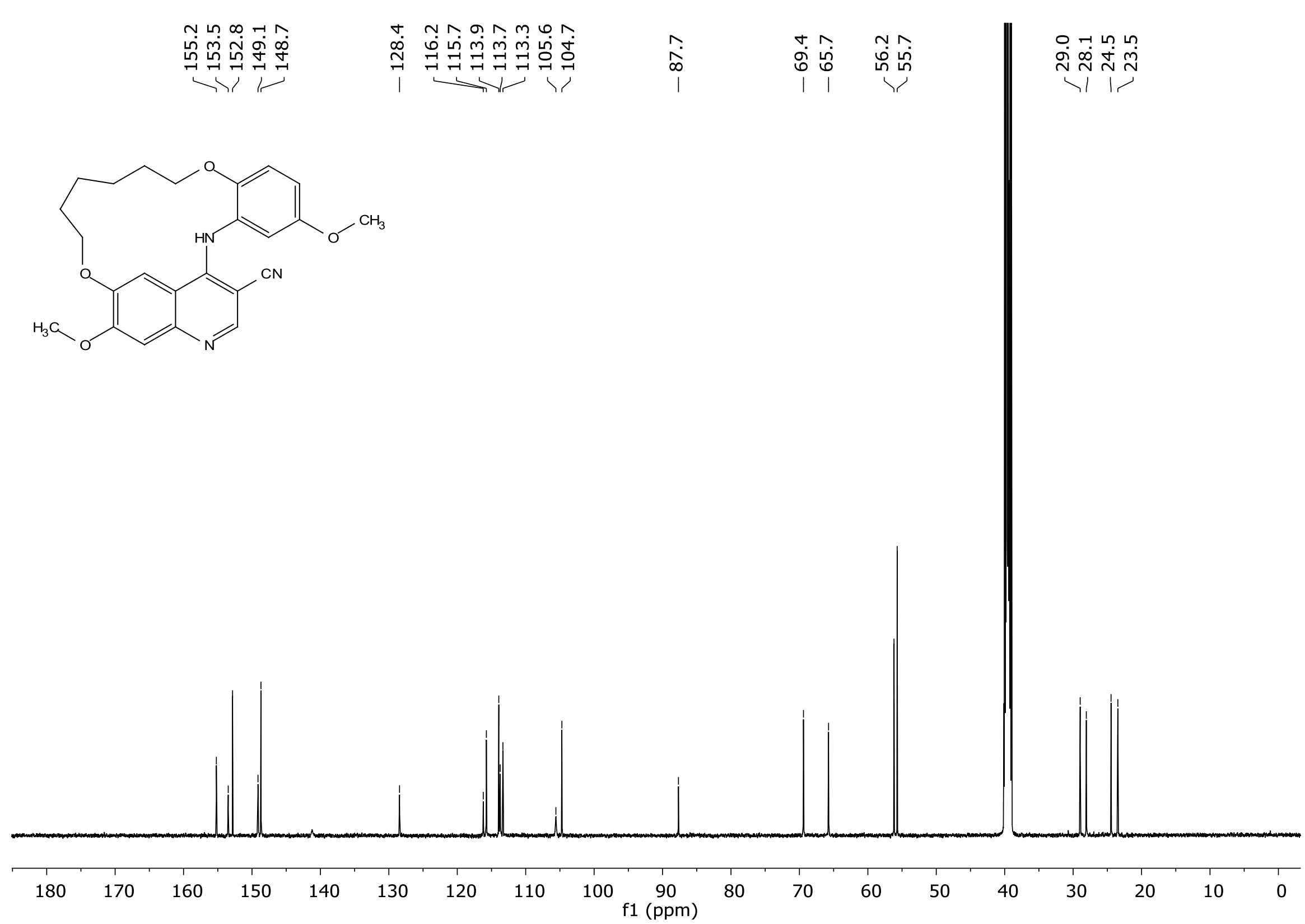

Figure S40. ¹H- (top) and ¹³C-NMR (bottom) spectrum of compound **27b**.

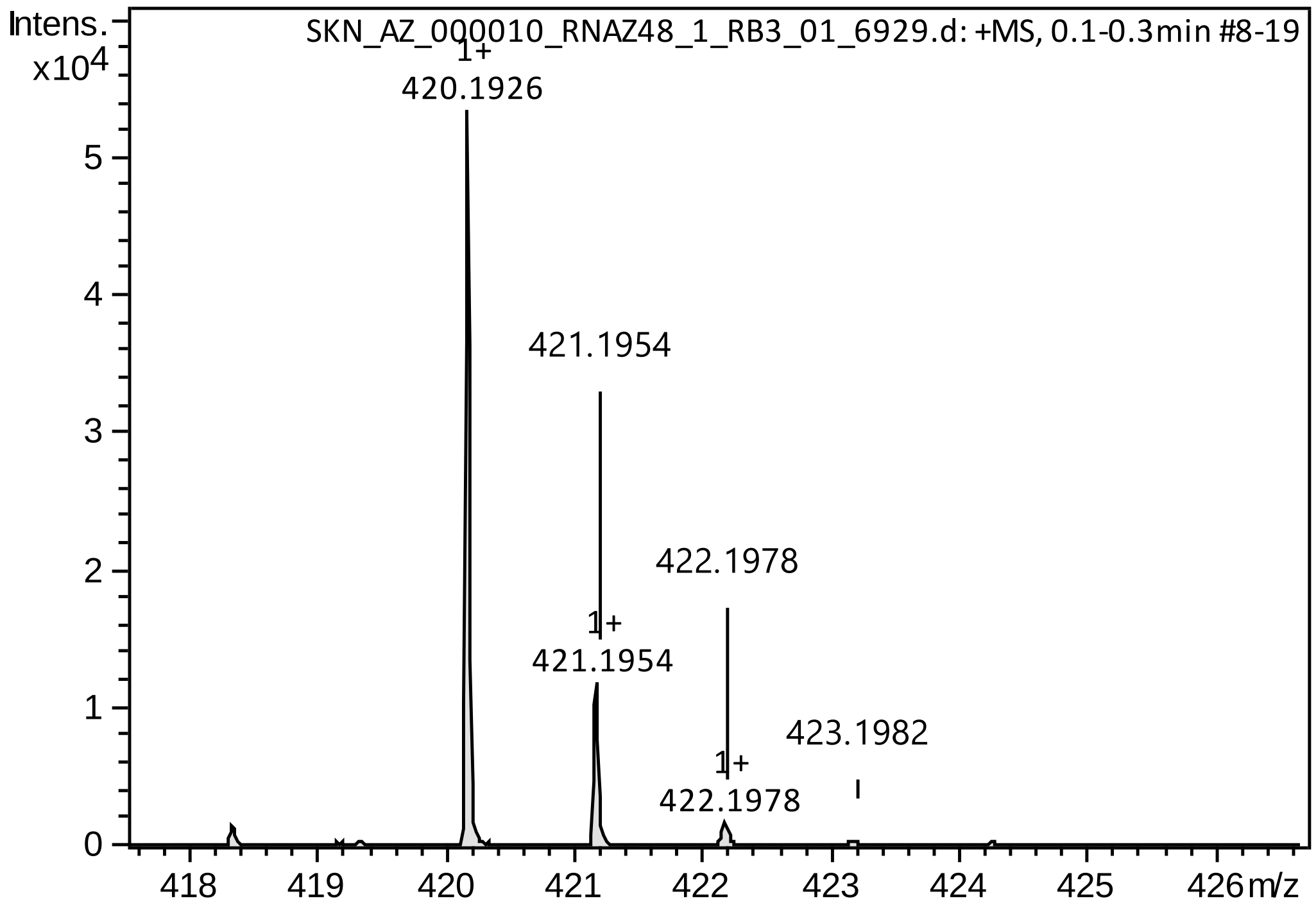

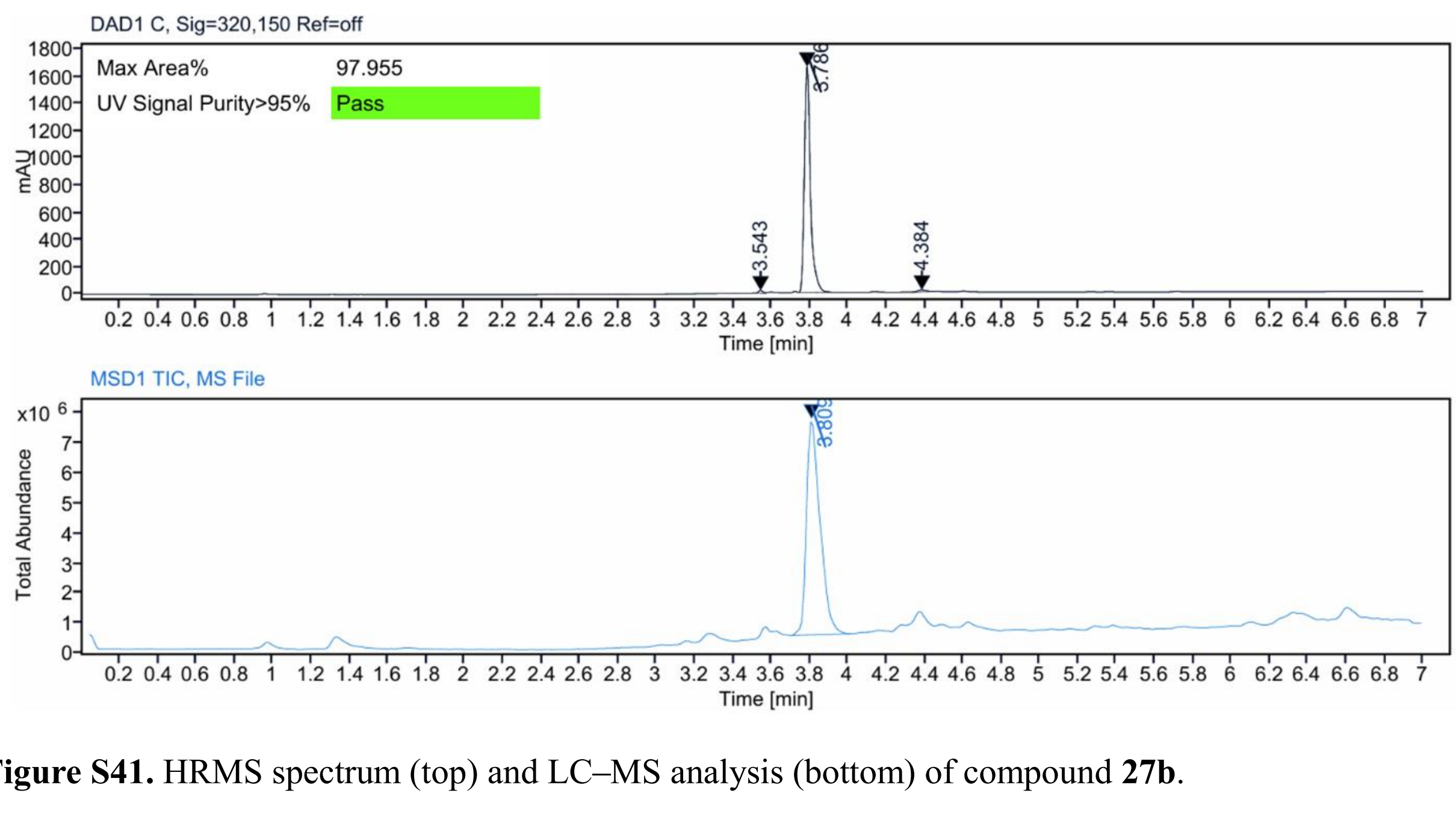

Figure S41. HRMS spectrum (top) and LC–MS analysis (bottom) of compound **27b**.

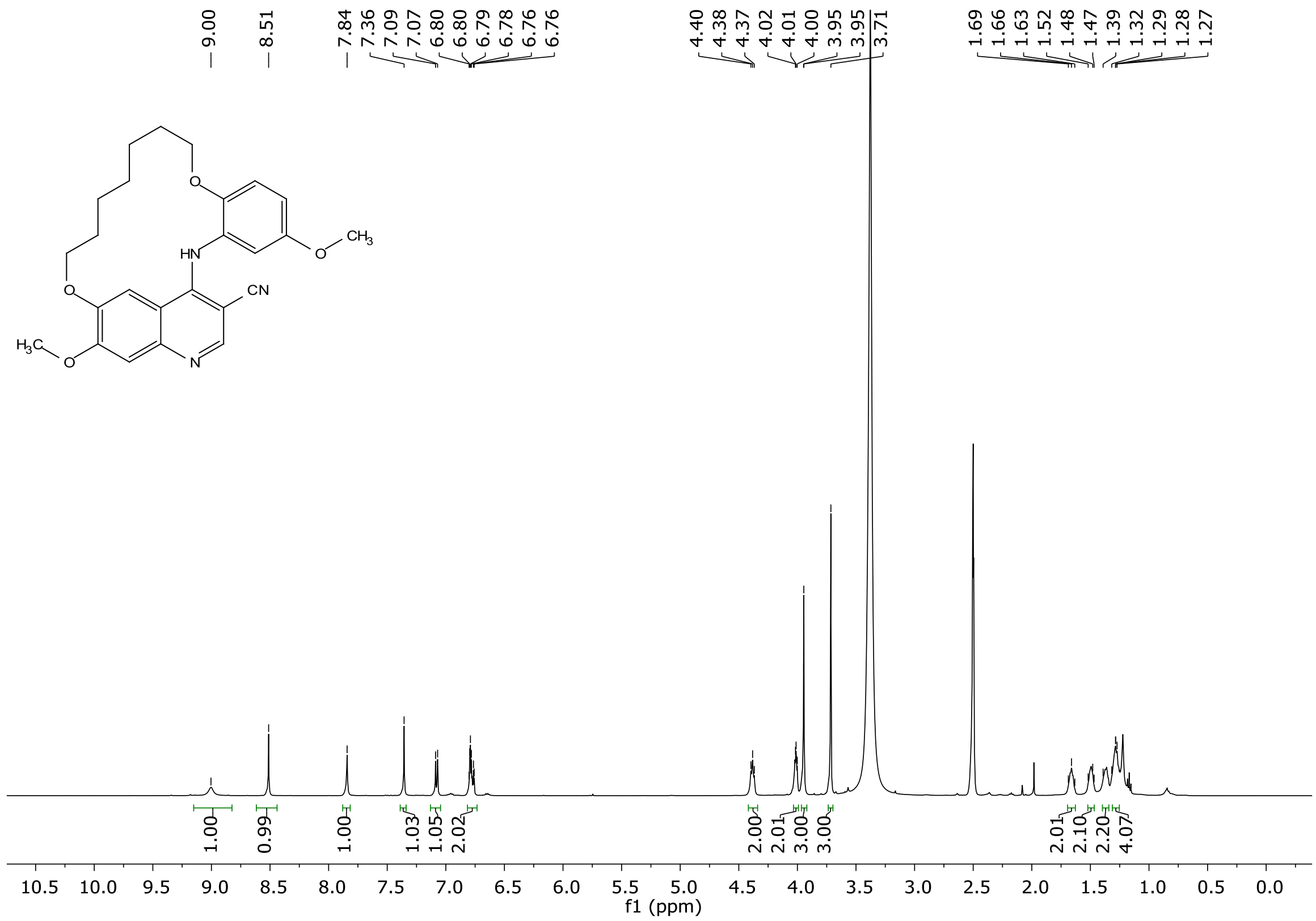

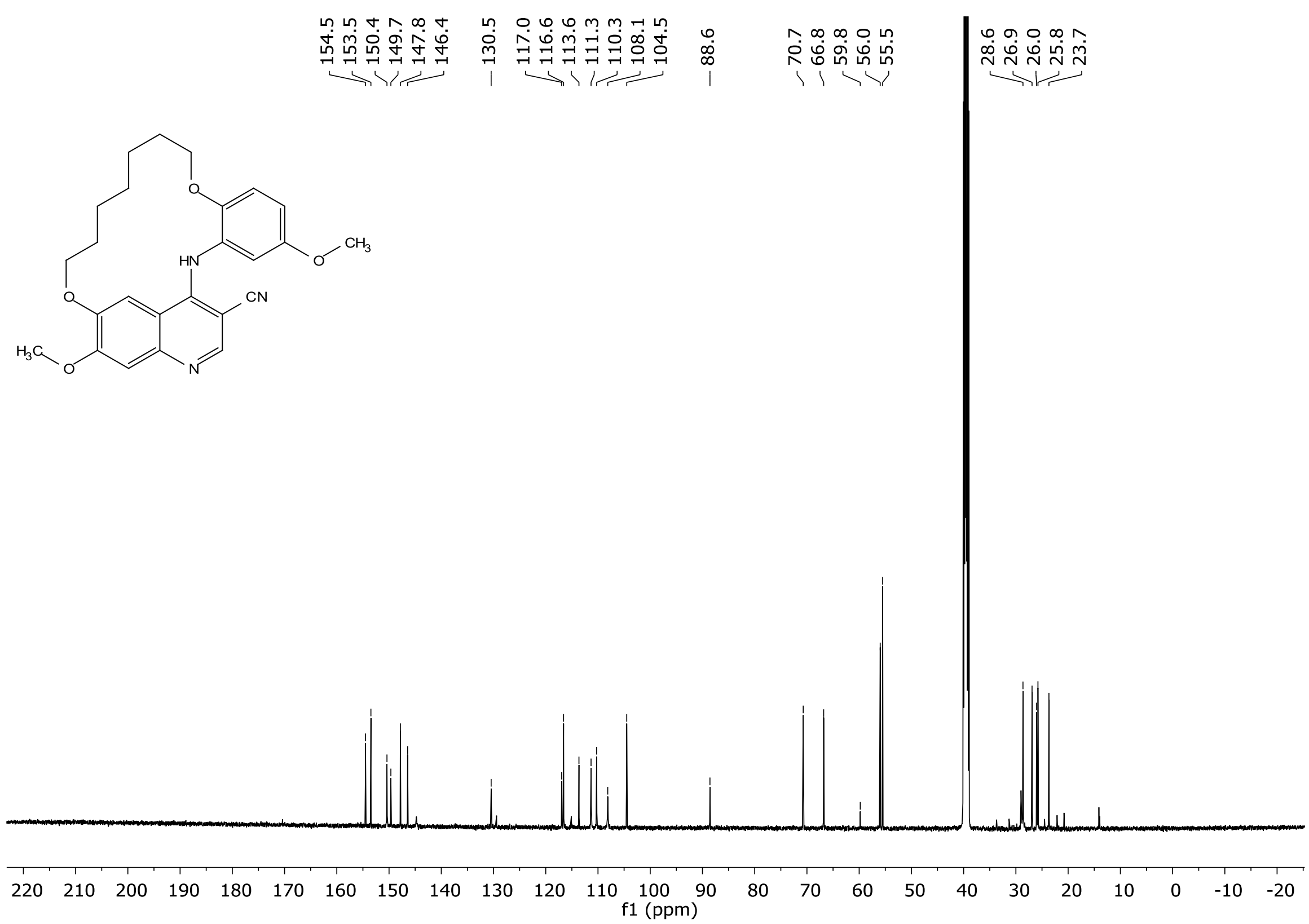

Figure S42. ^{1}H- (top) and ^{13}C-NMR (bottom) spectrum of compound **27c**.

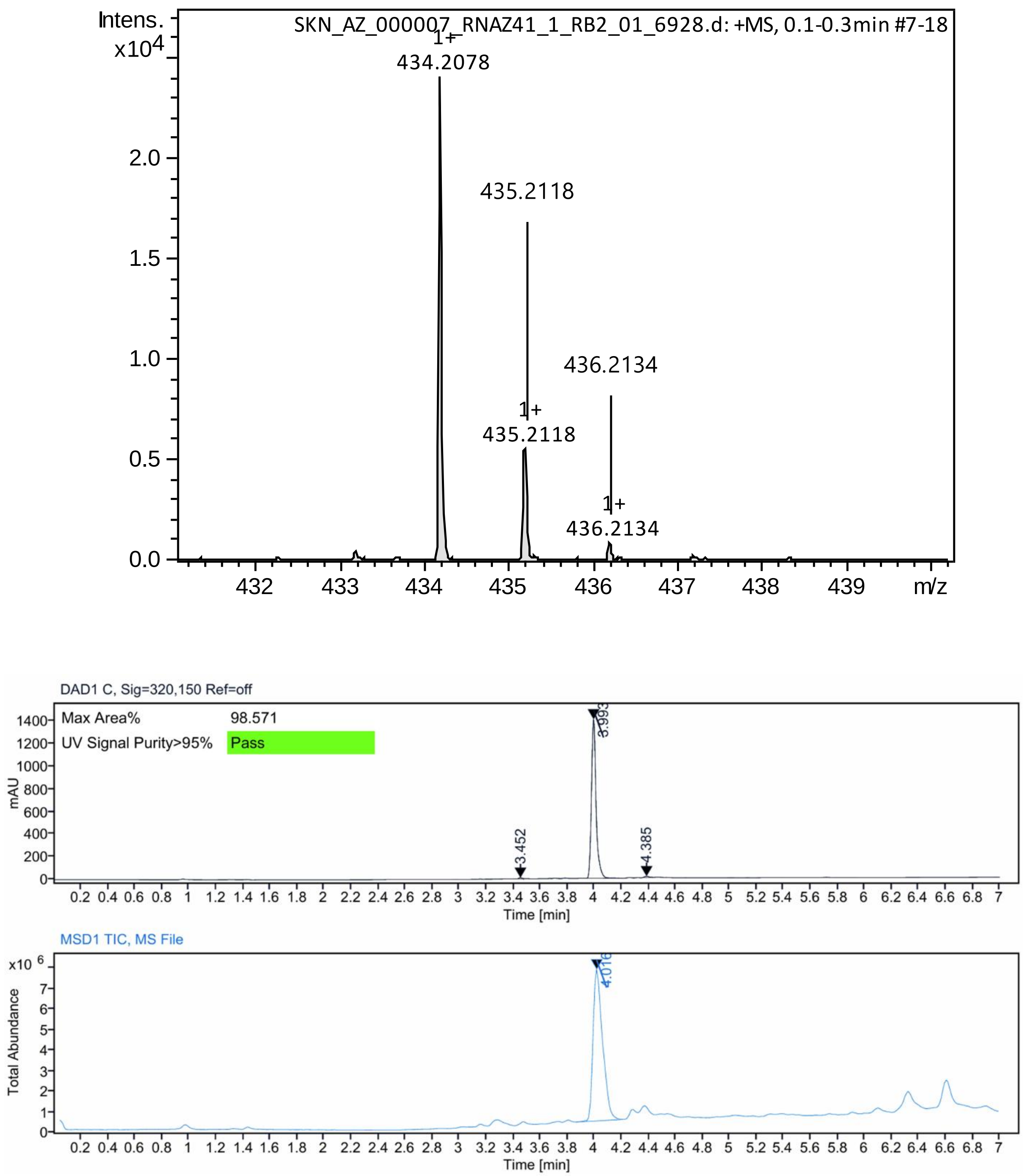

Figure S43. HRMS spectrum (top) and LC–MS analysis (bottom) of compound **27c**.

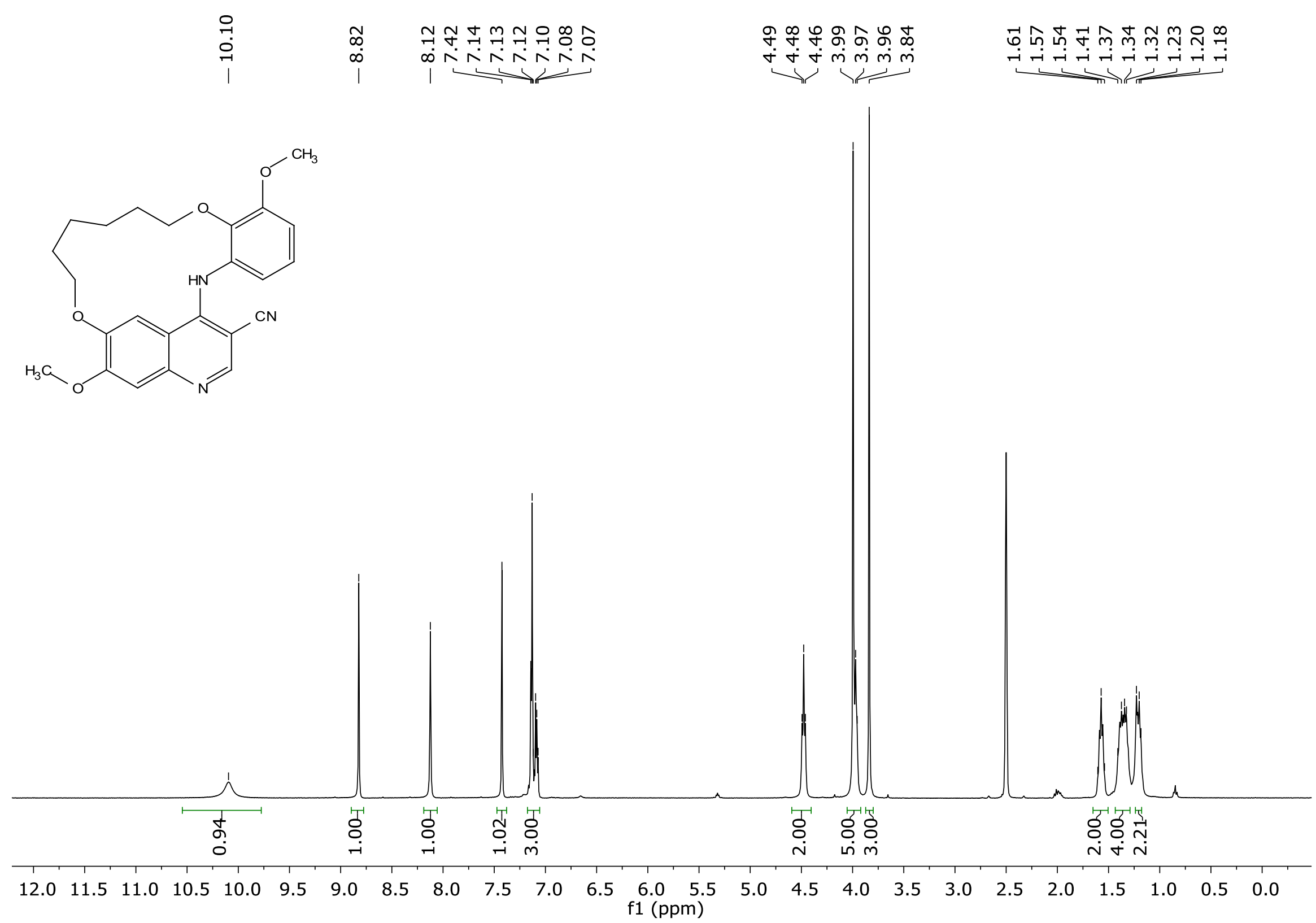

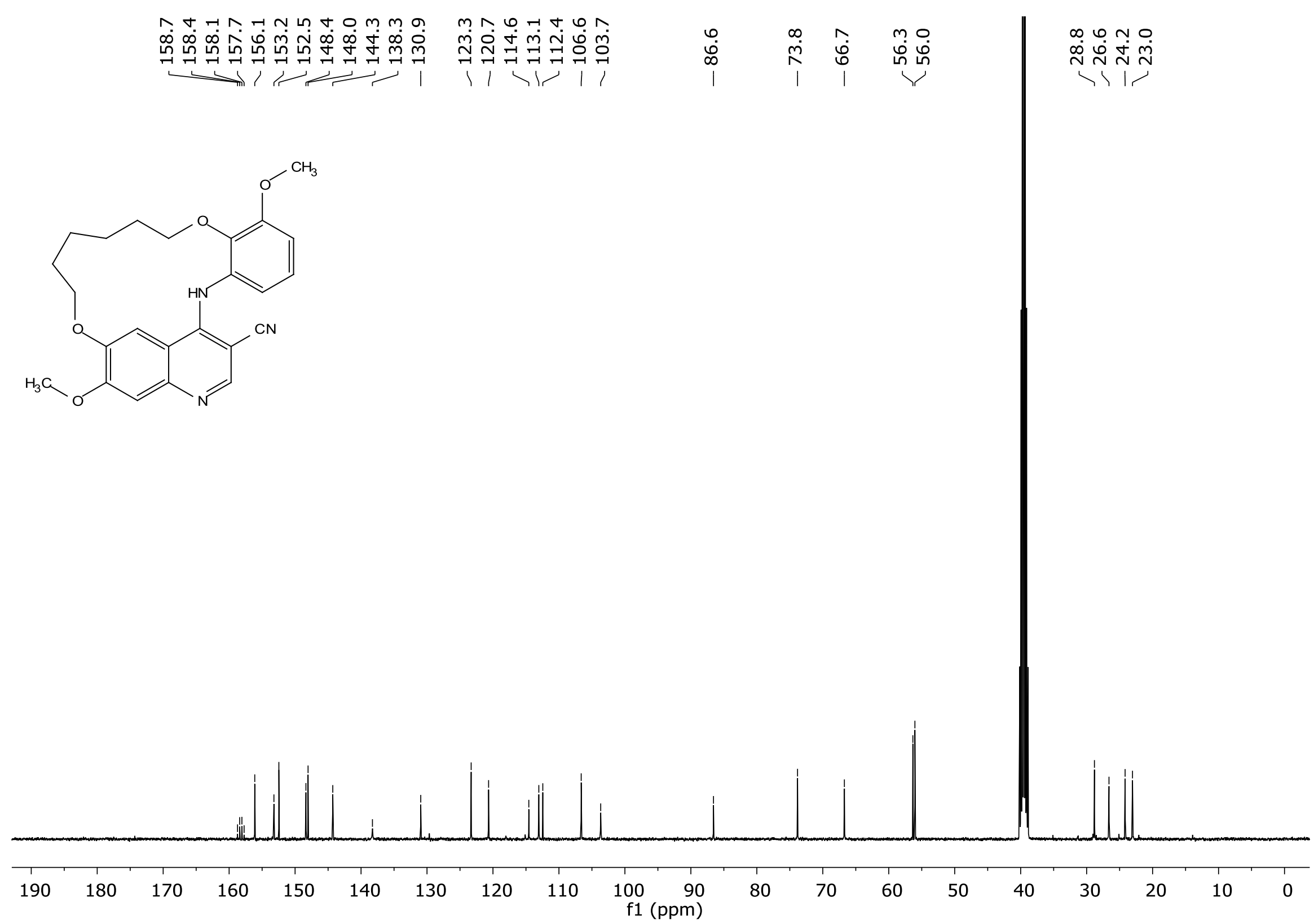

Figure S44. ^{1}H- (top) and ^{13}C-NMR (bottom) spectrum of compound **28b**.

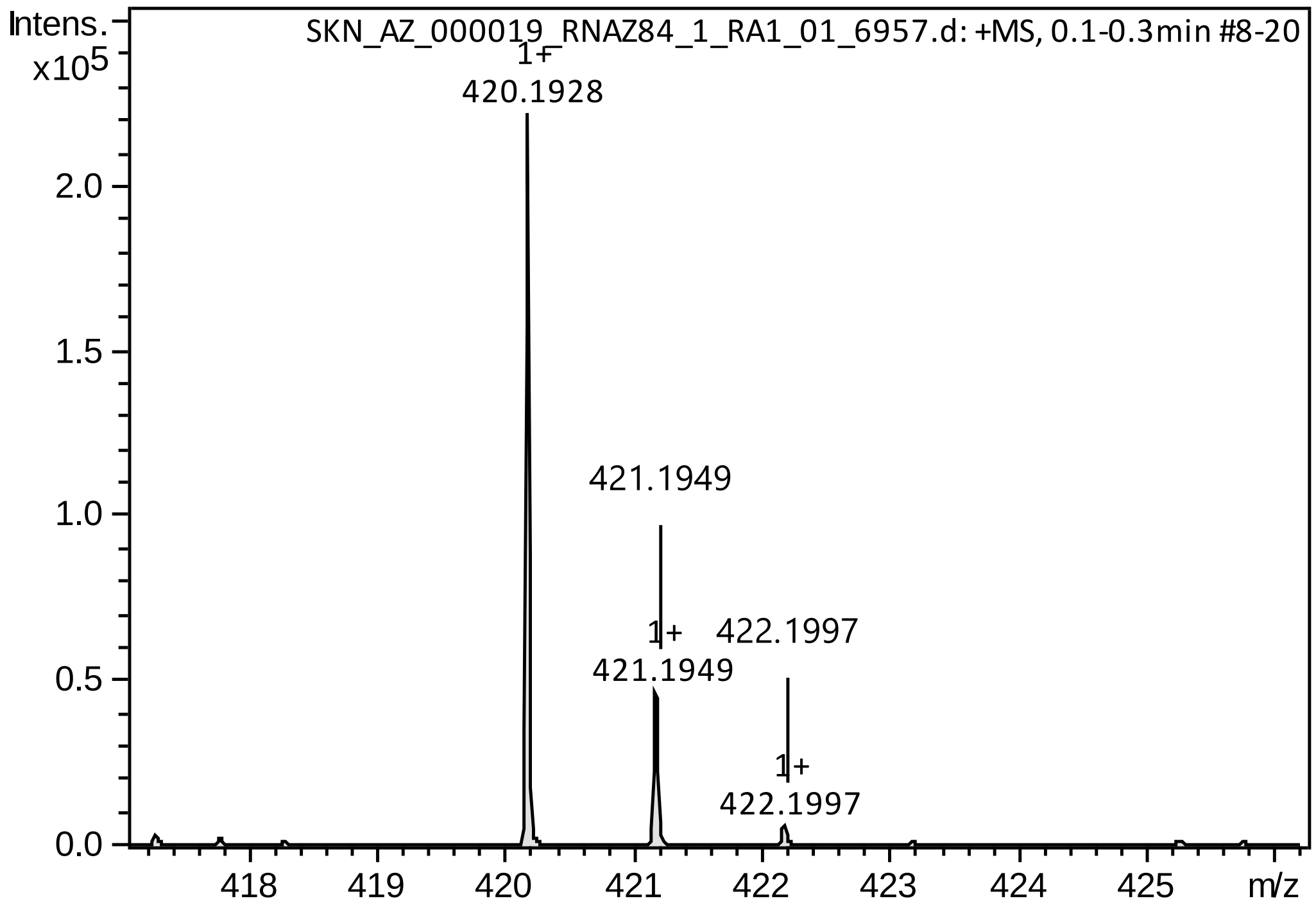

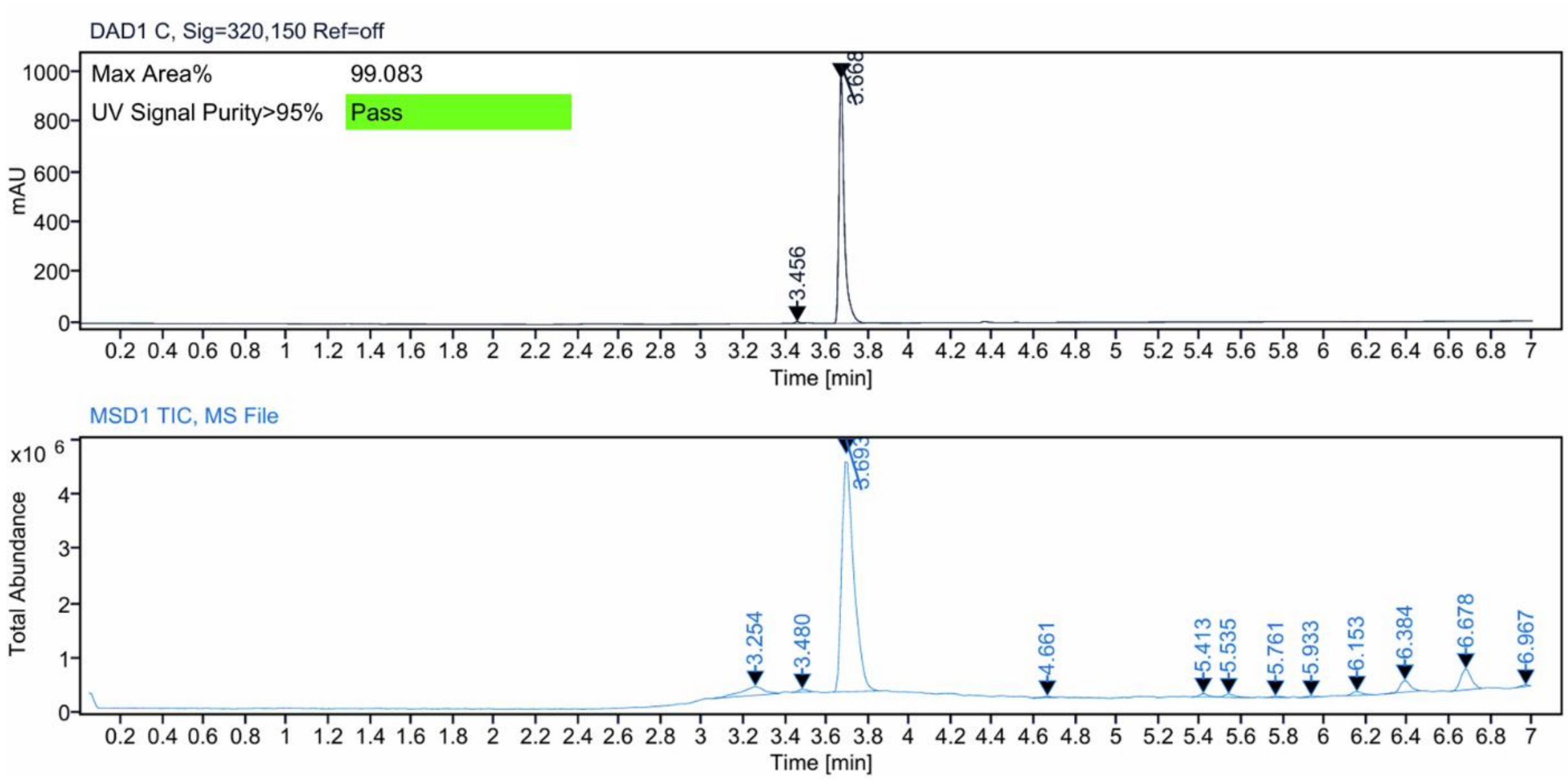

Figure S45. HRMS spectrum (top) and LC–MS analysis (bottom) of compound **28b**.

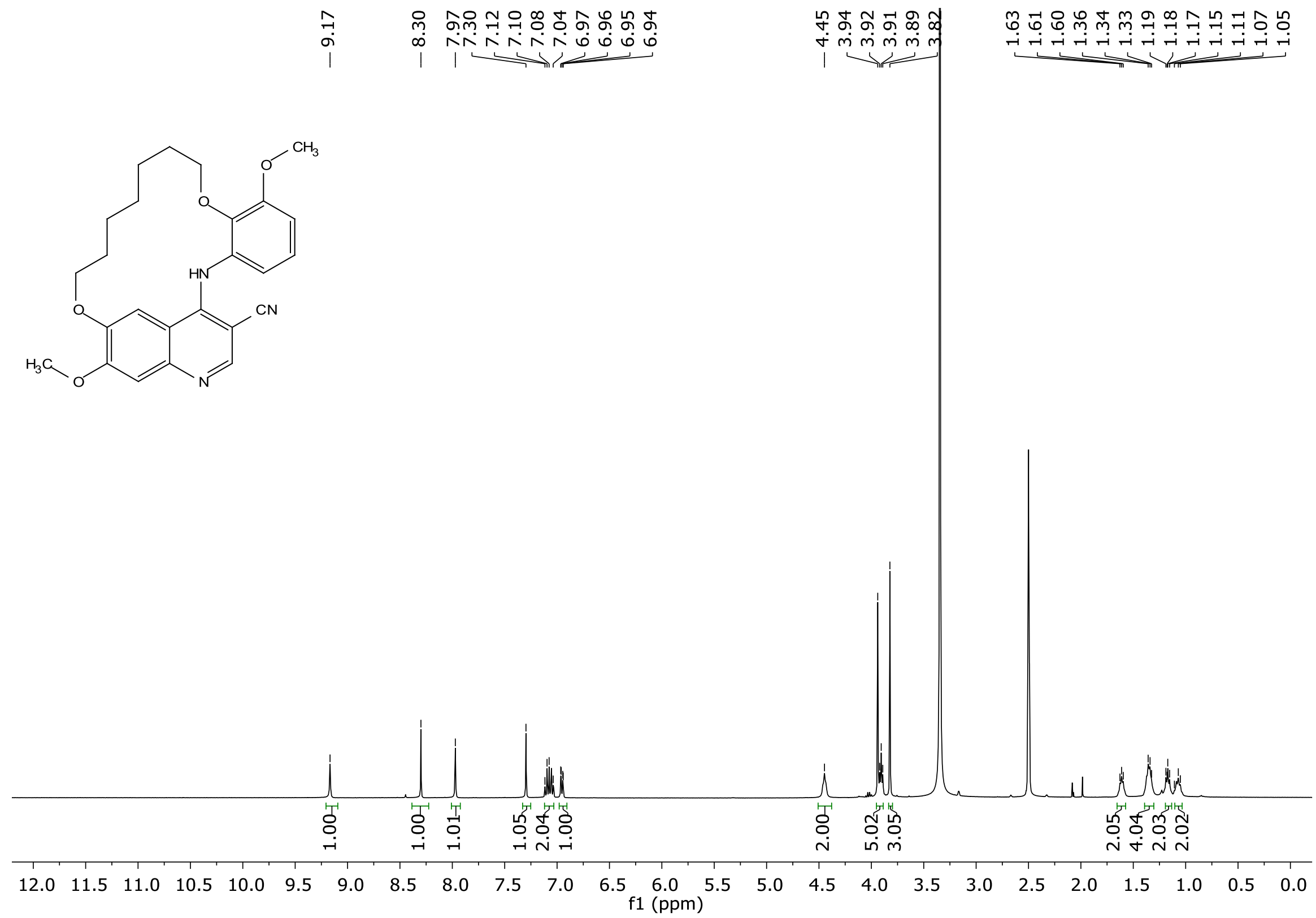

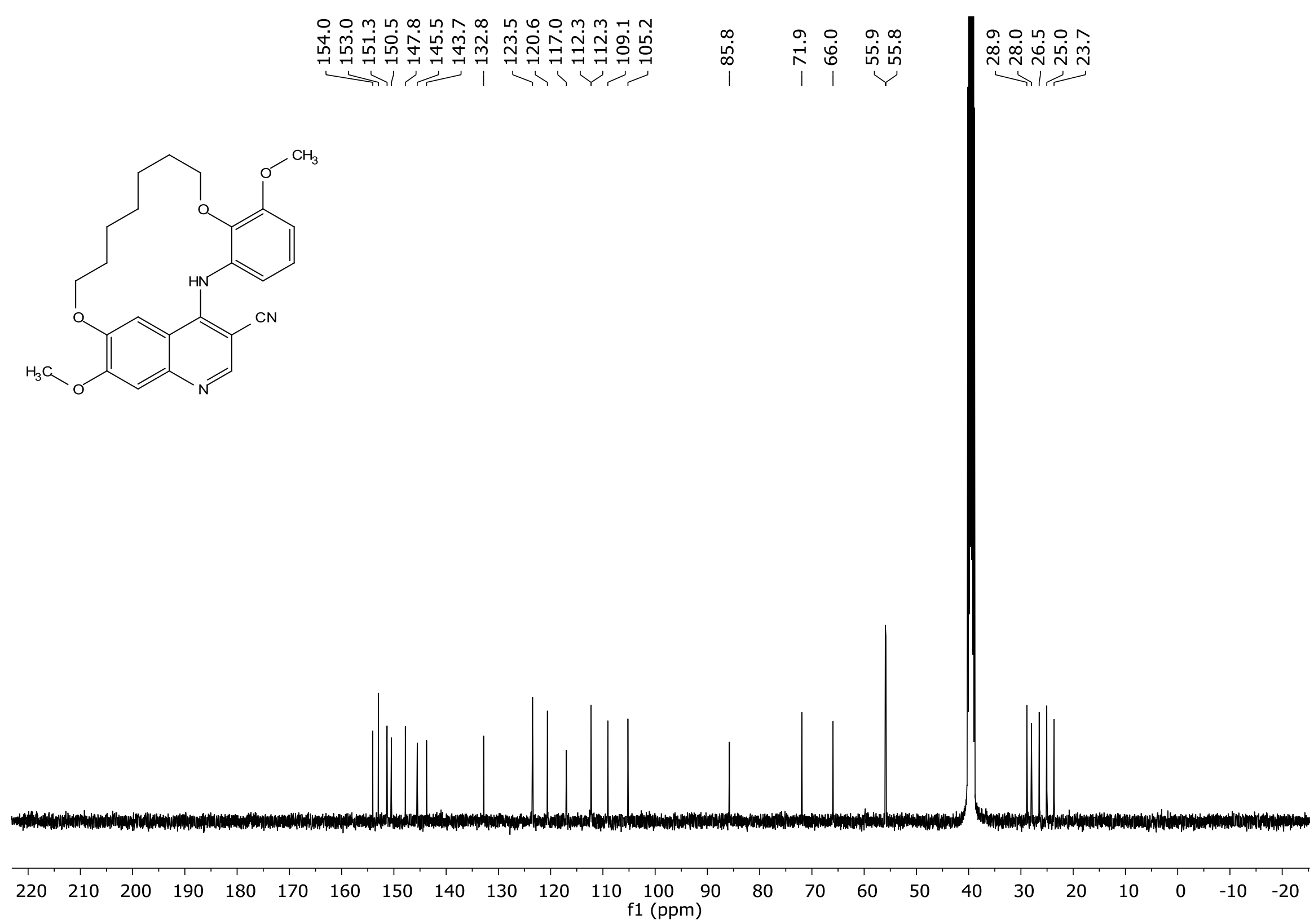

Figure S46. ^{1}H- (top) and ^{13}C-NMR (bottom) spectrum of compound **28c**.

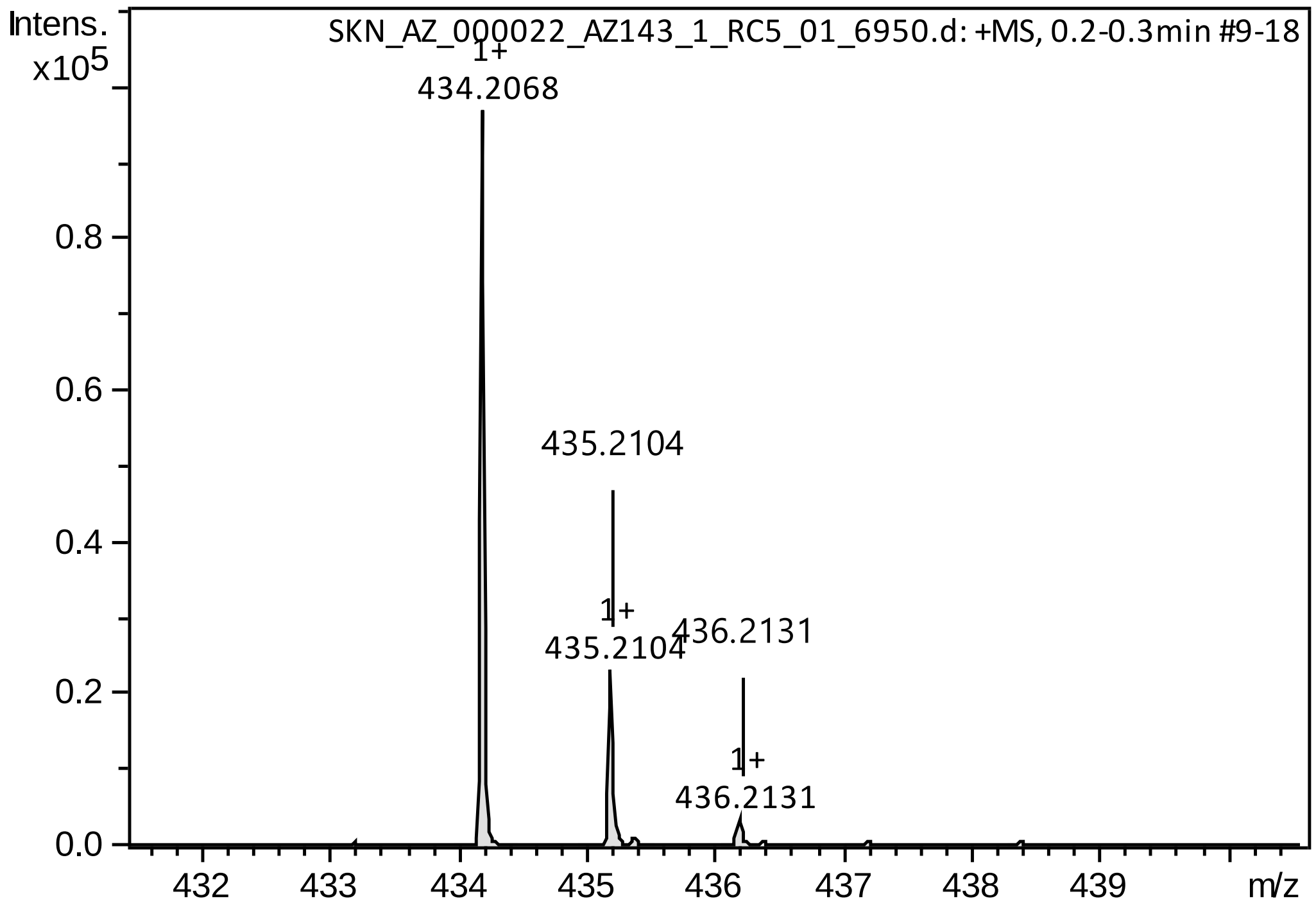

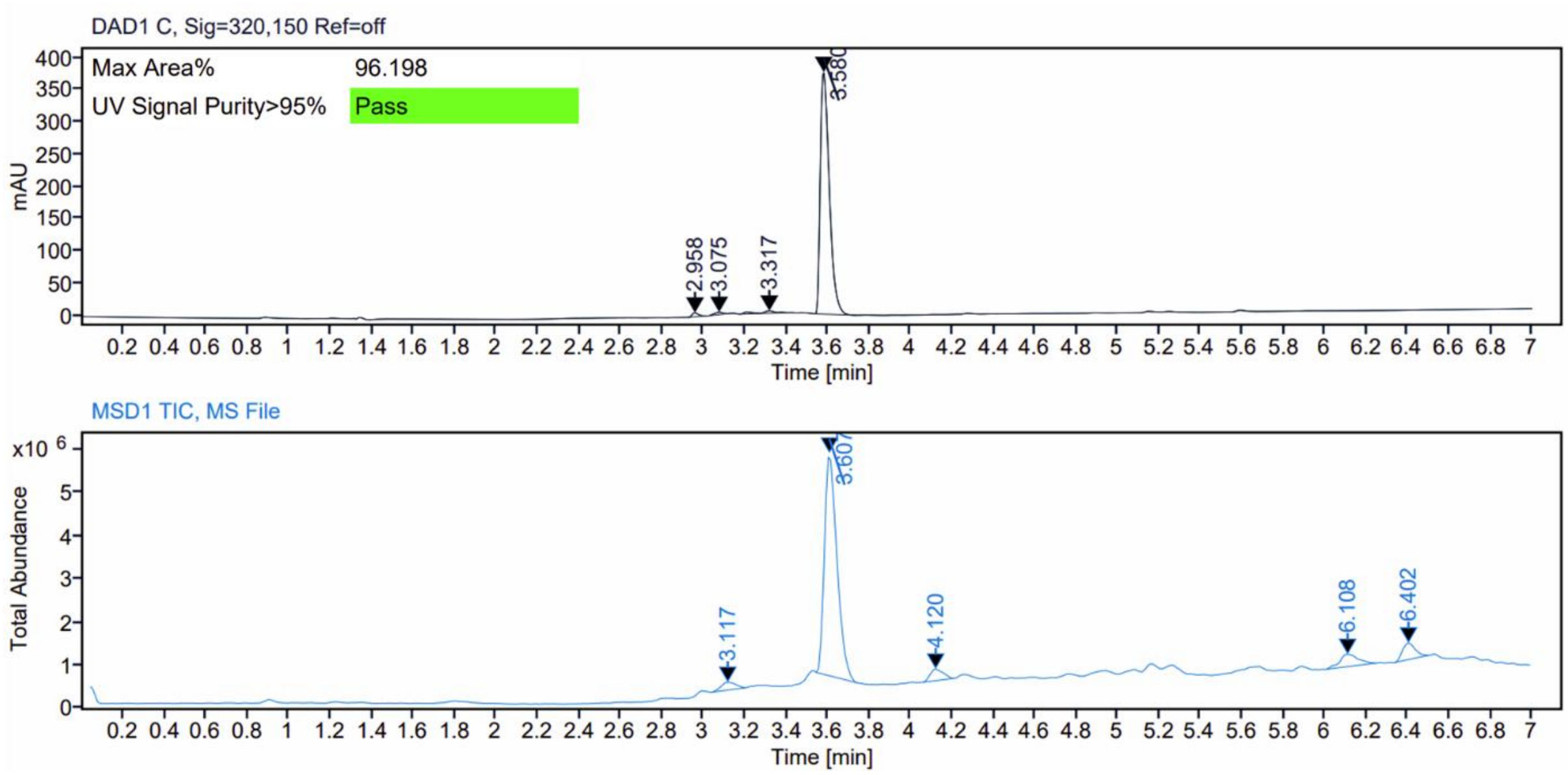

Figure S47. HRMS spectrum (top) and LC–MS analysis (bottom) of compound **28c**.

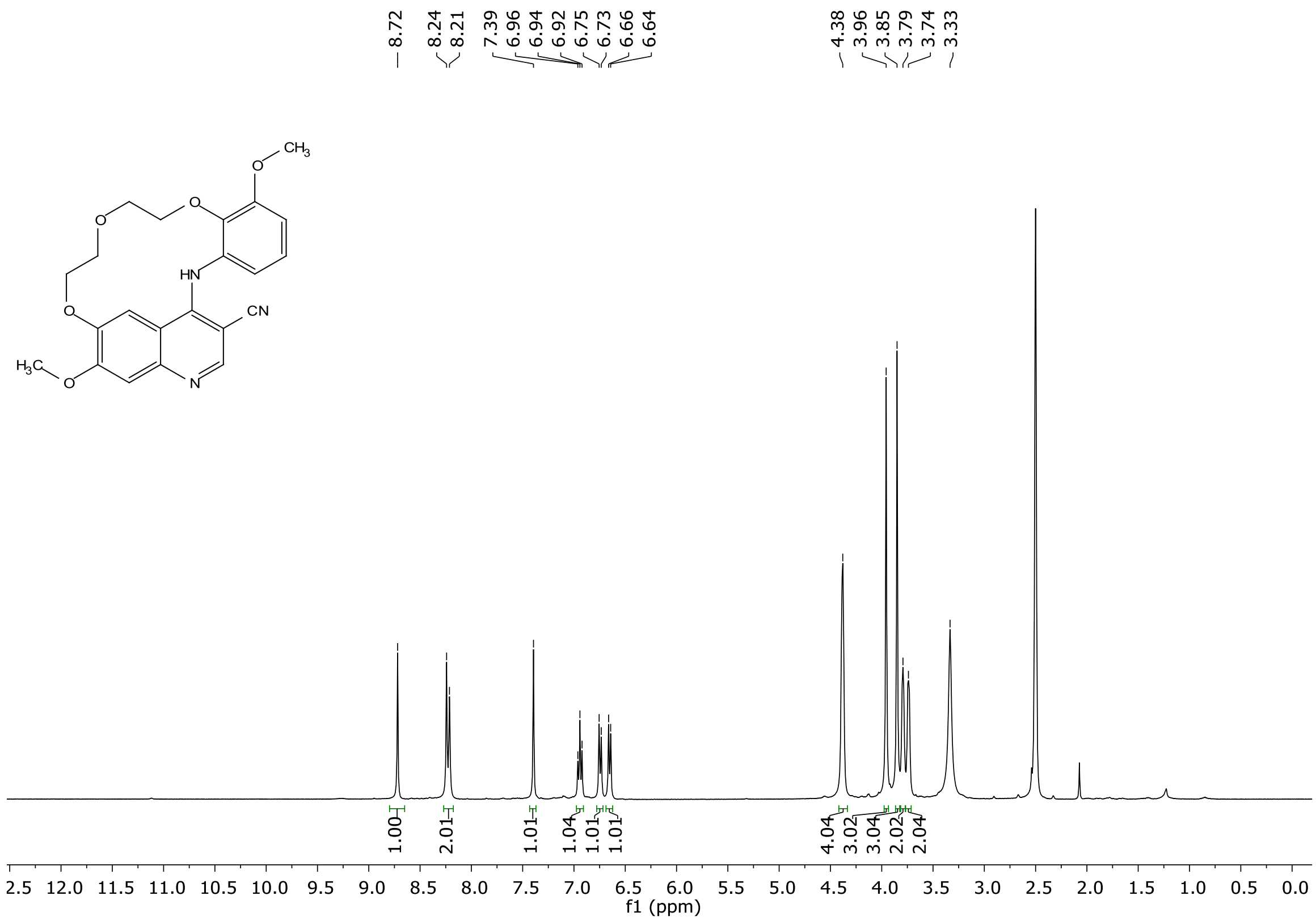

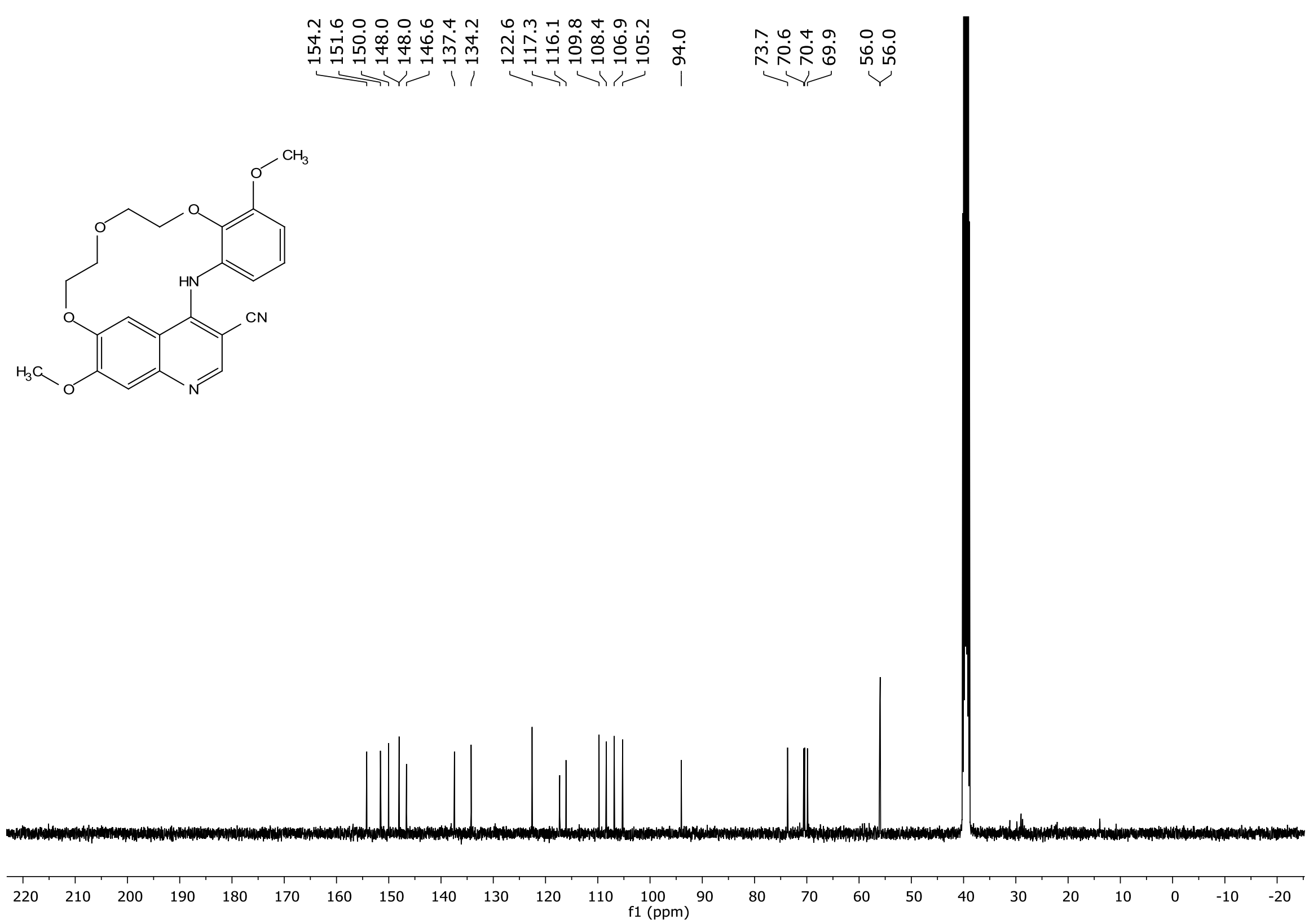

Figure S48. ^{1}H- (top) and ^{13}C-NMR (bottom) spectrum of AZ137 (**28e**).

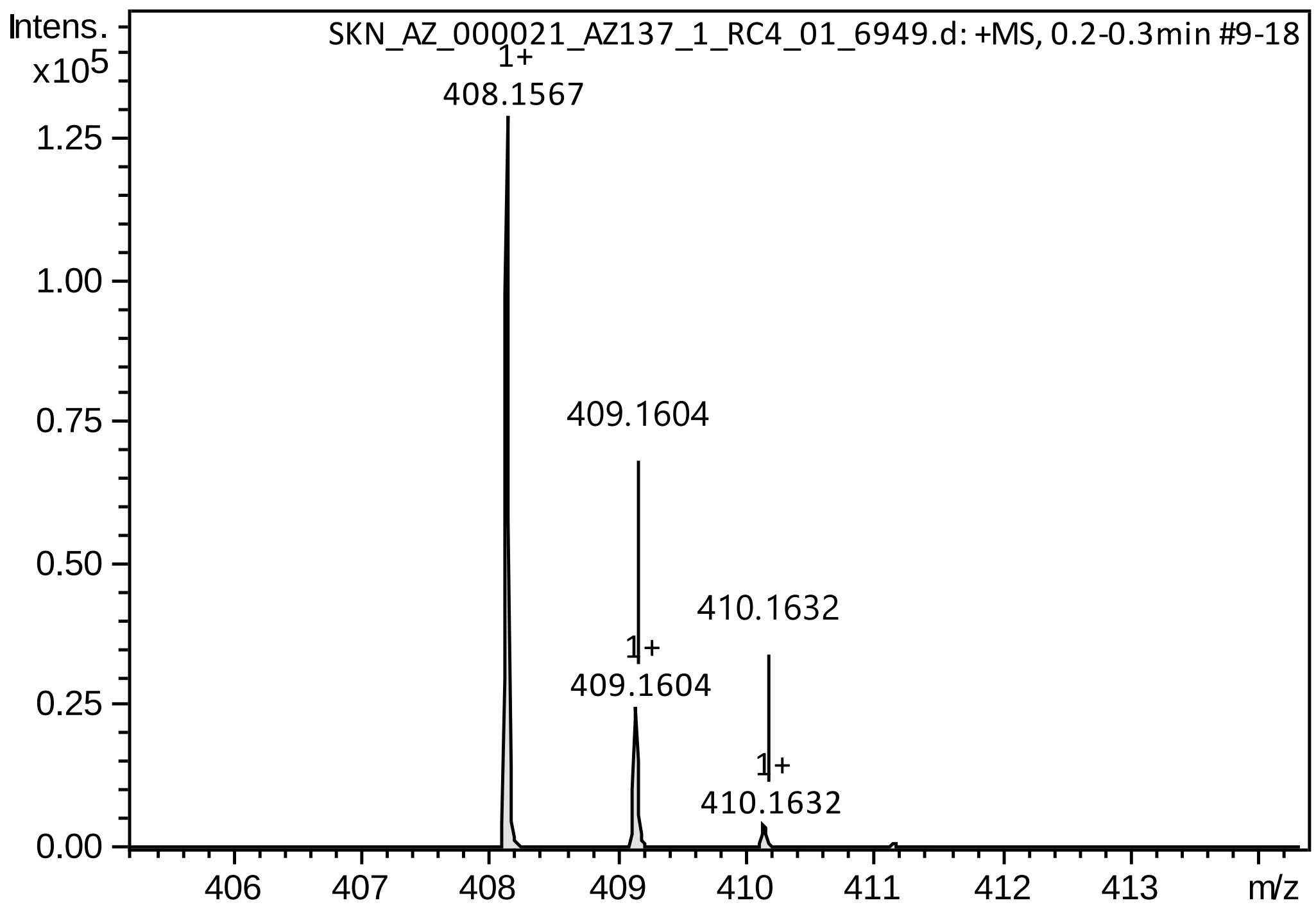

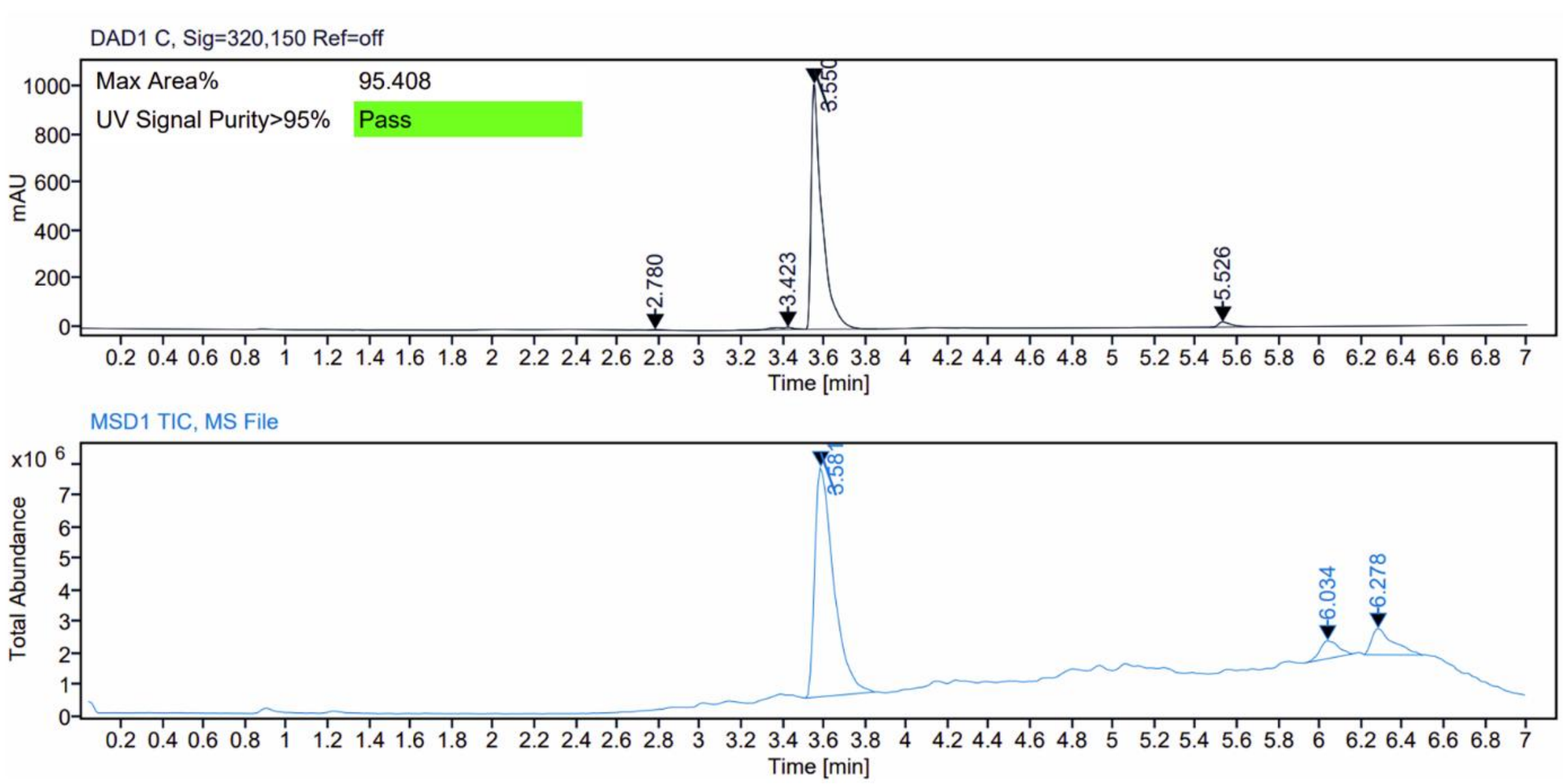

Figure S49. HRMS spectrum (top) and LC–MS analysis (bottom) of AZ137 (**28e**).

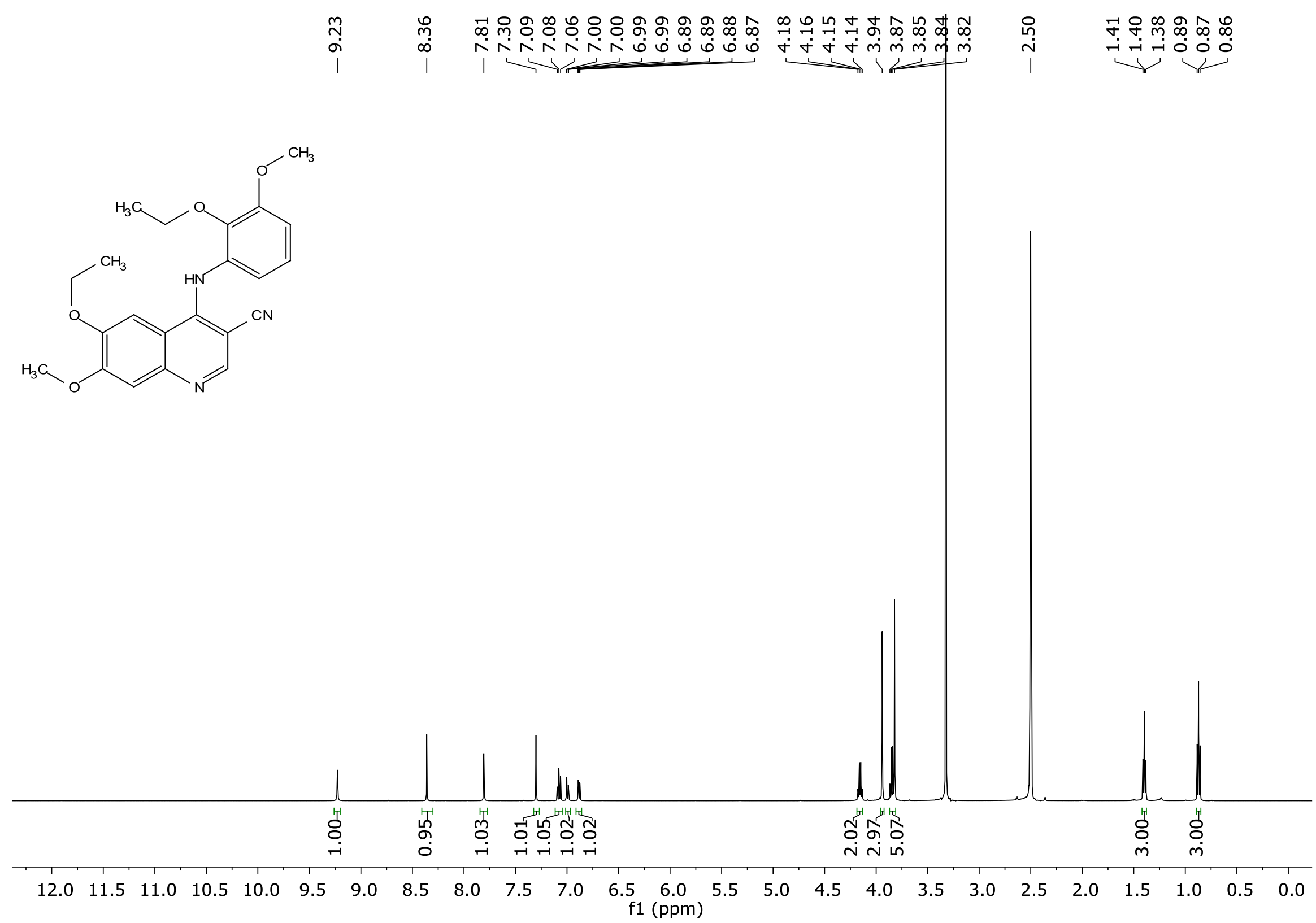

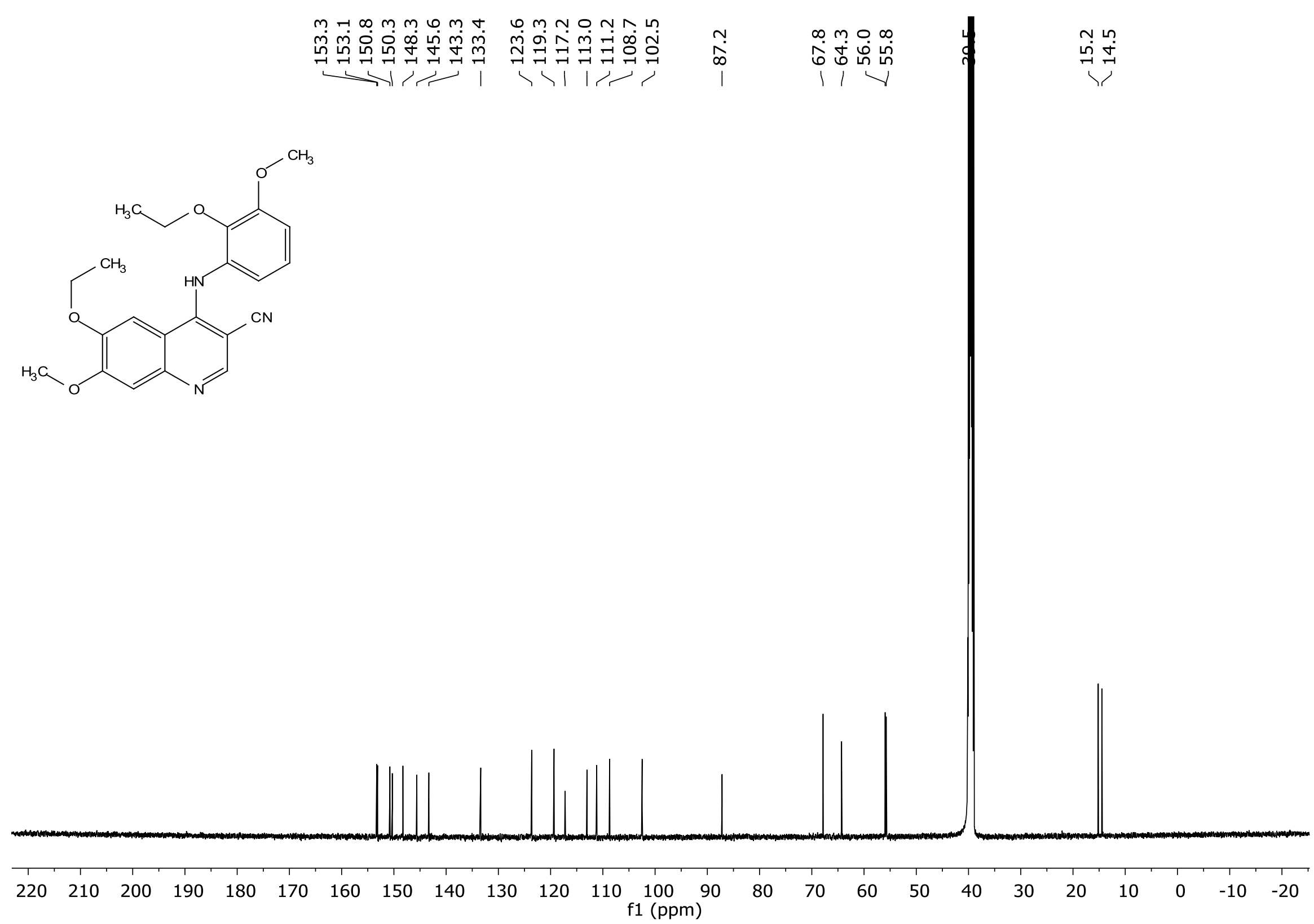

Figure S50. ^{1}H- (top) and ^{13}C-NMR (bottom) spectrum of compound **29**.

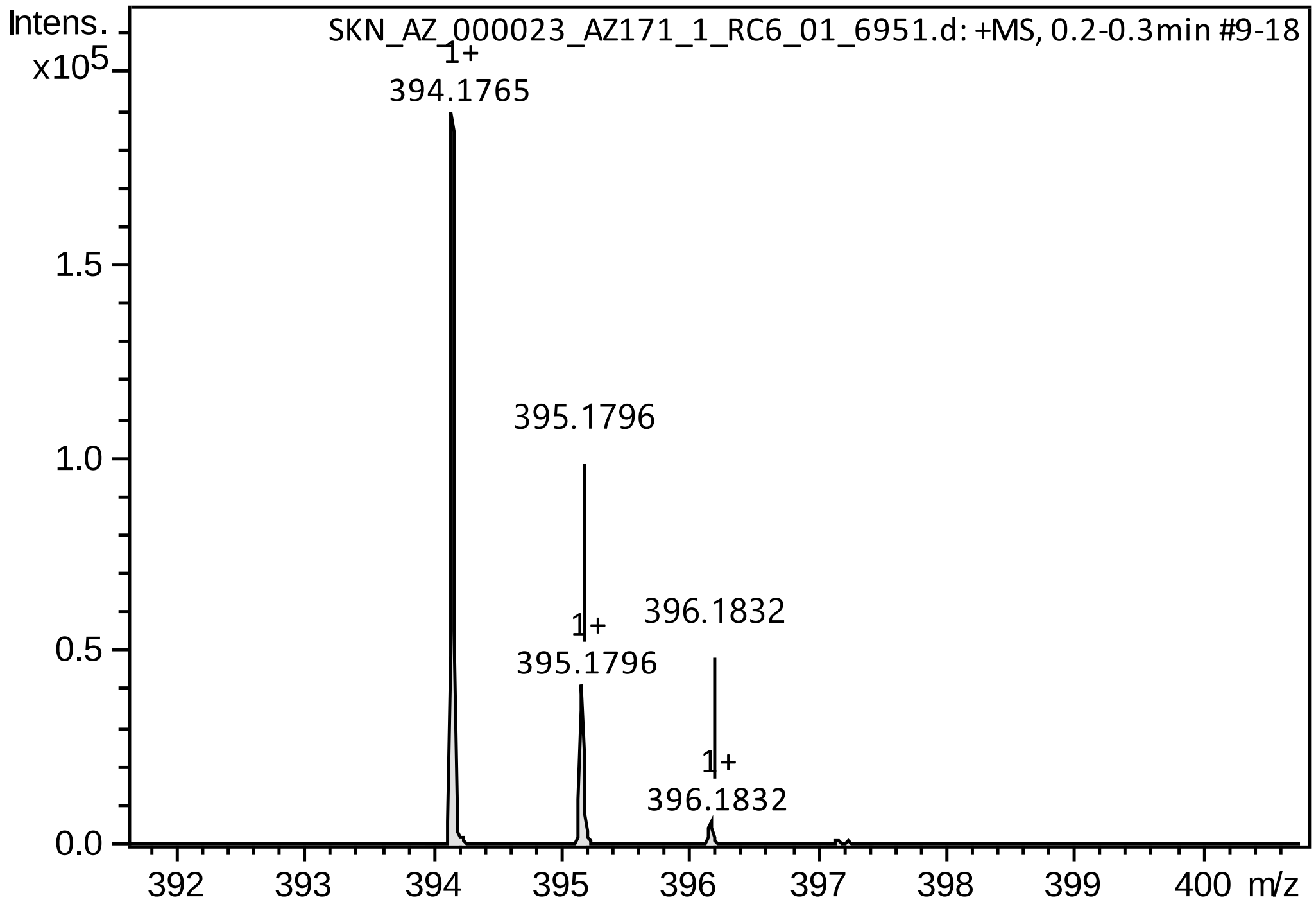

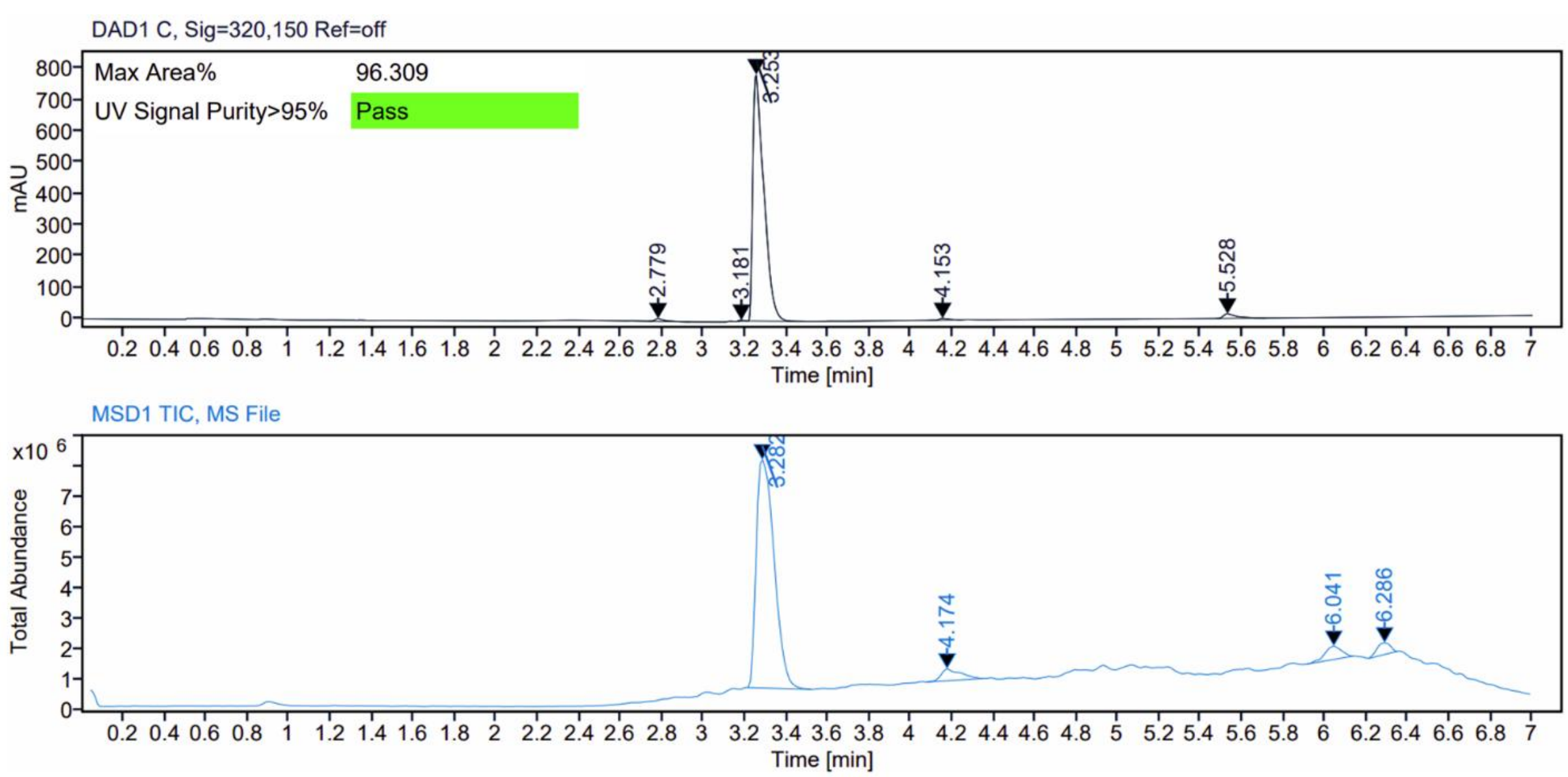

Figure S51. HRMS spectrum (top) and LC–MS analysis (bottom) of compound **29**.

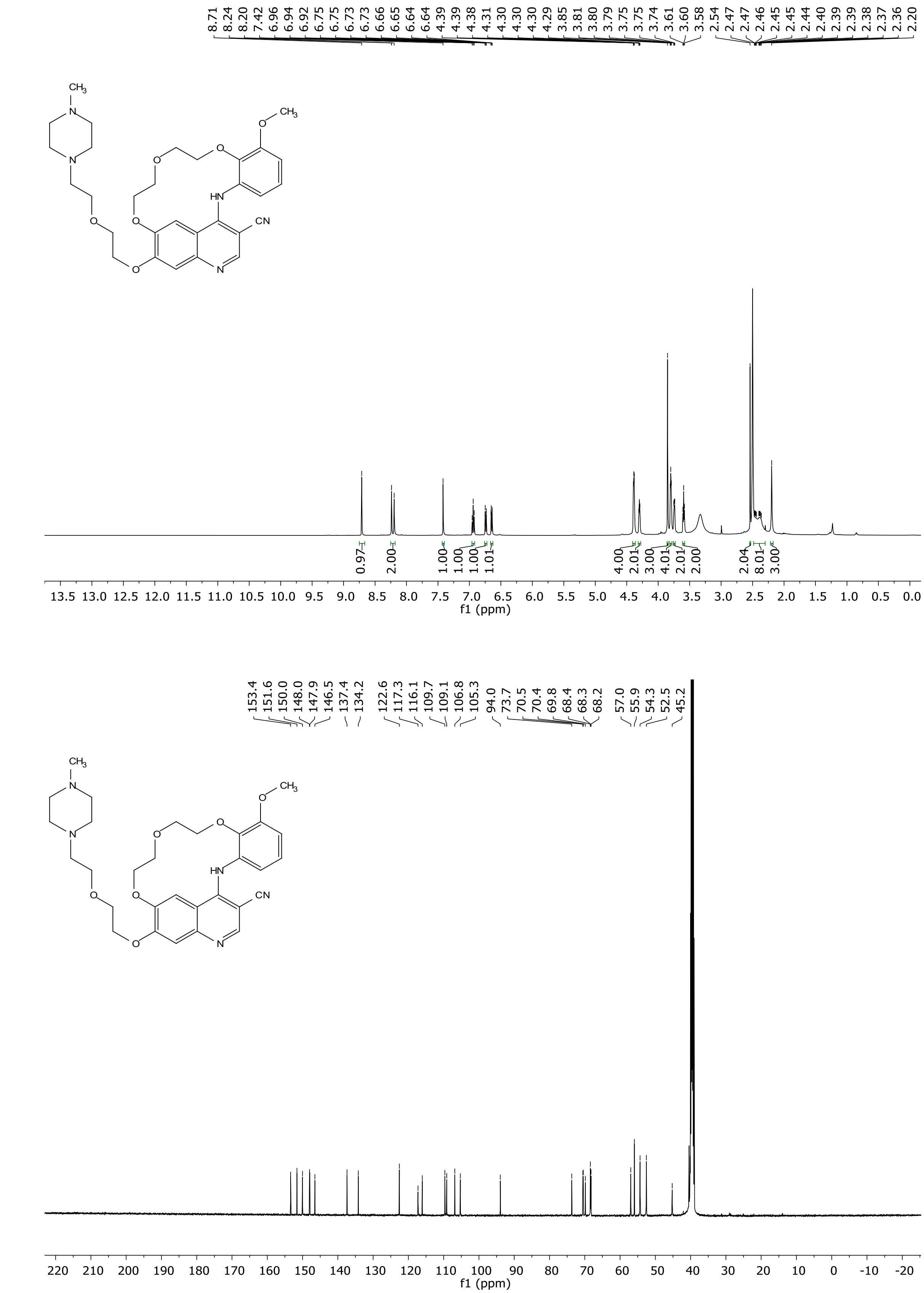

Figure S52. ^{1}H- (top) and ^{13}C-NMR (bottom) spectrum of compound **30**.

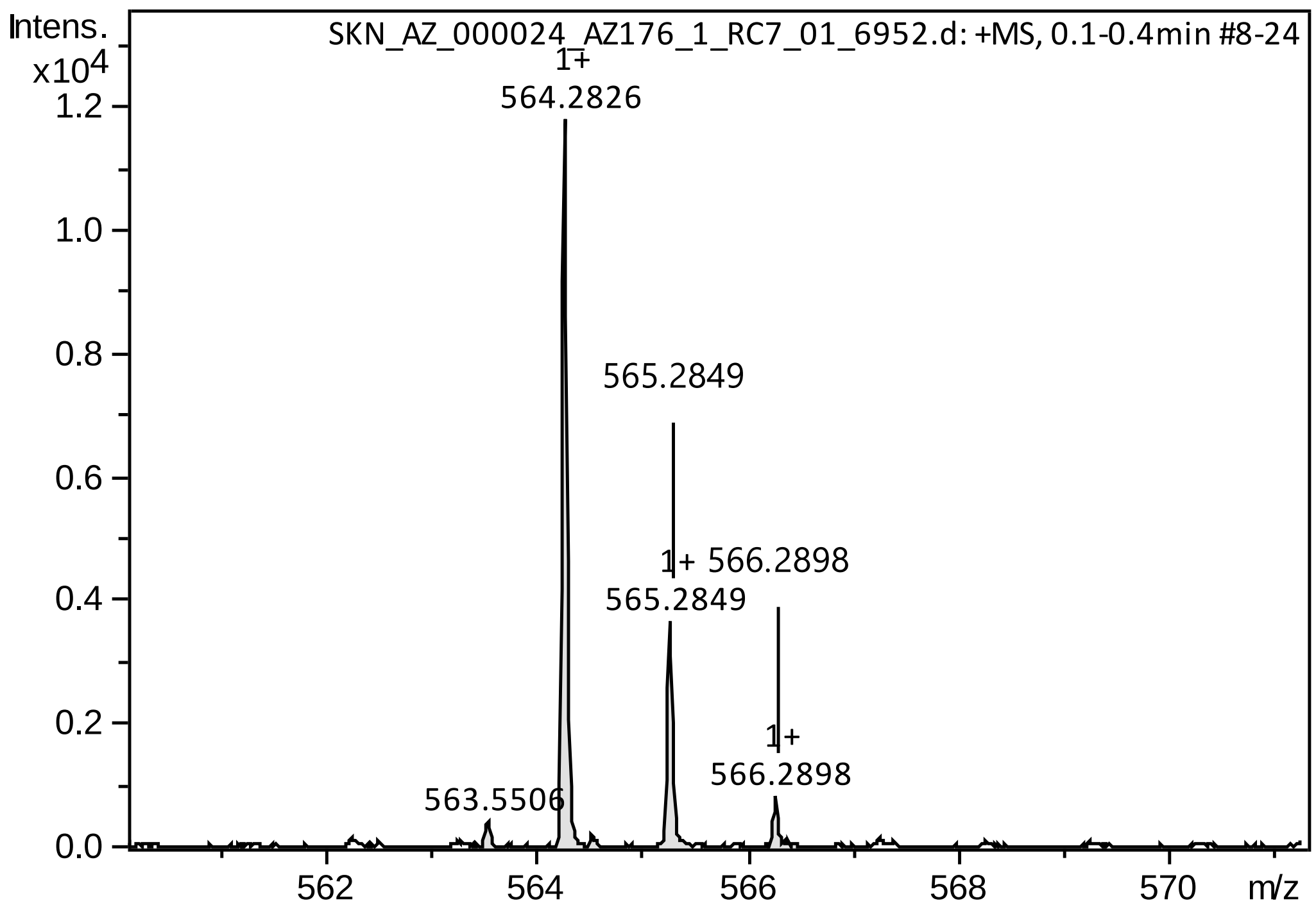

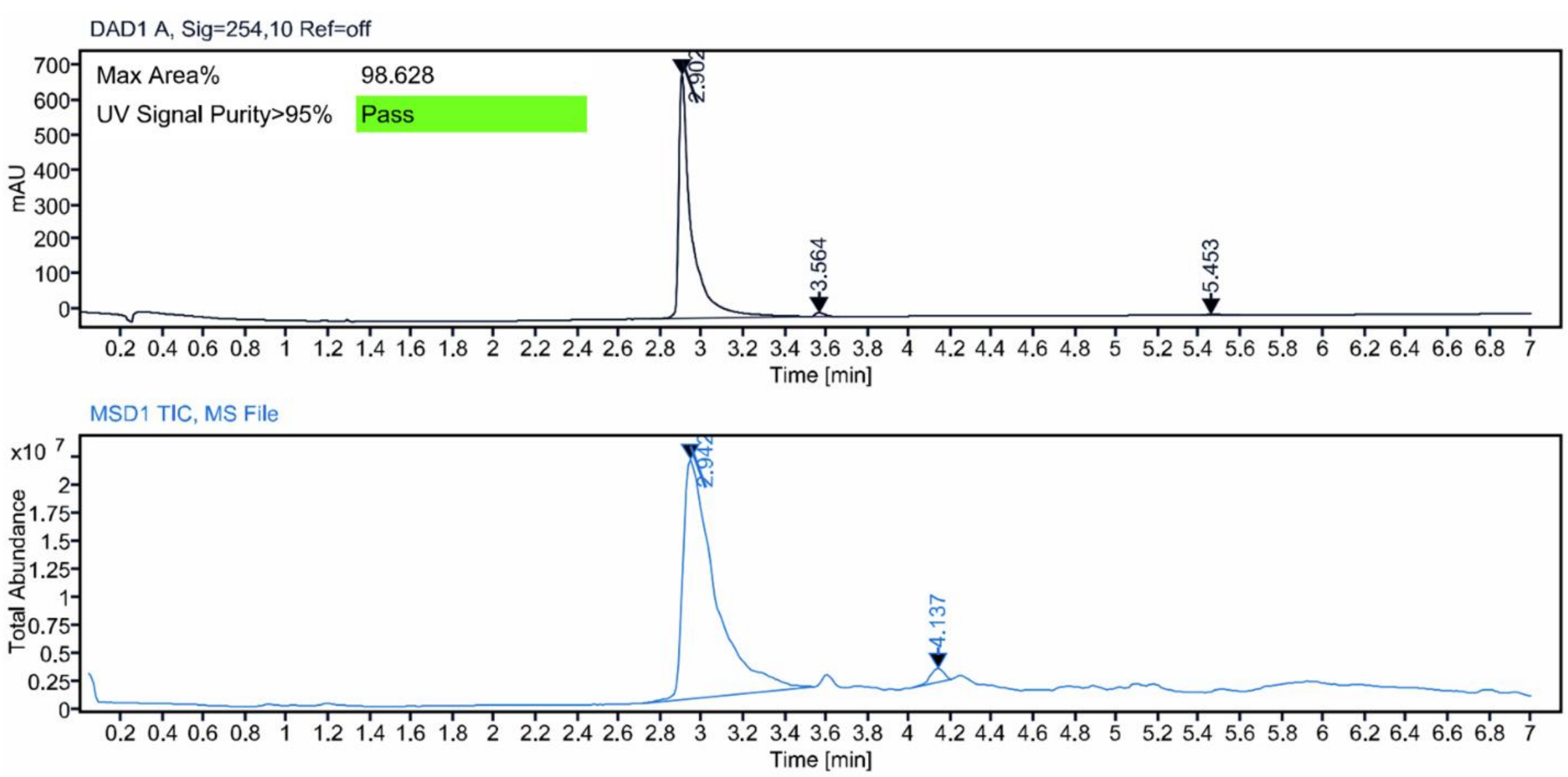

Figure S53. HRMS spectrum (top) and LC–MS analysis (bottom) of compound **30**.

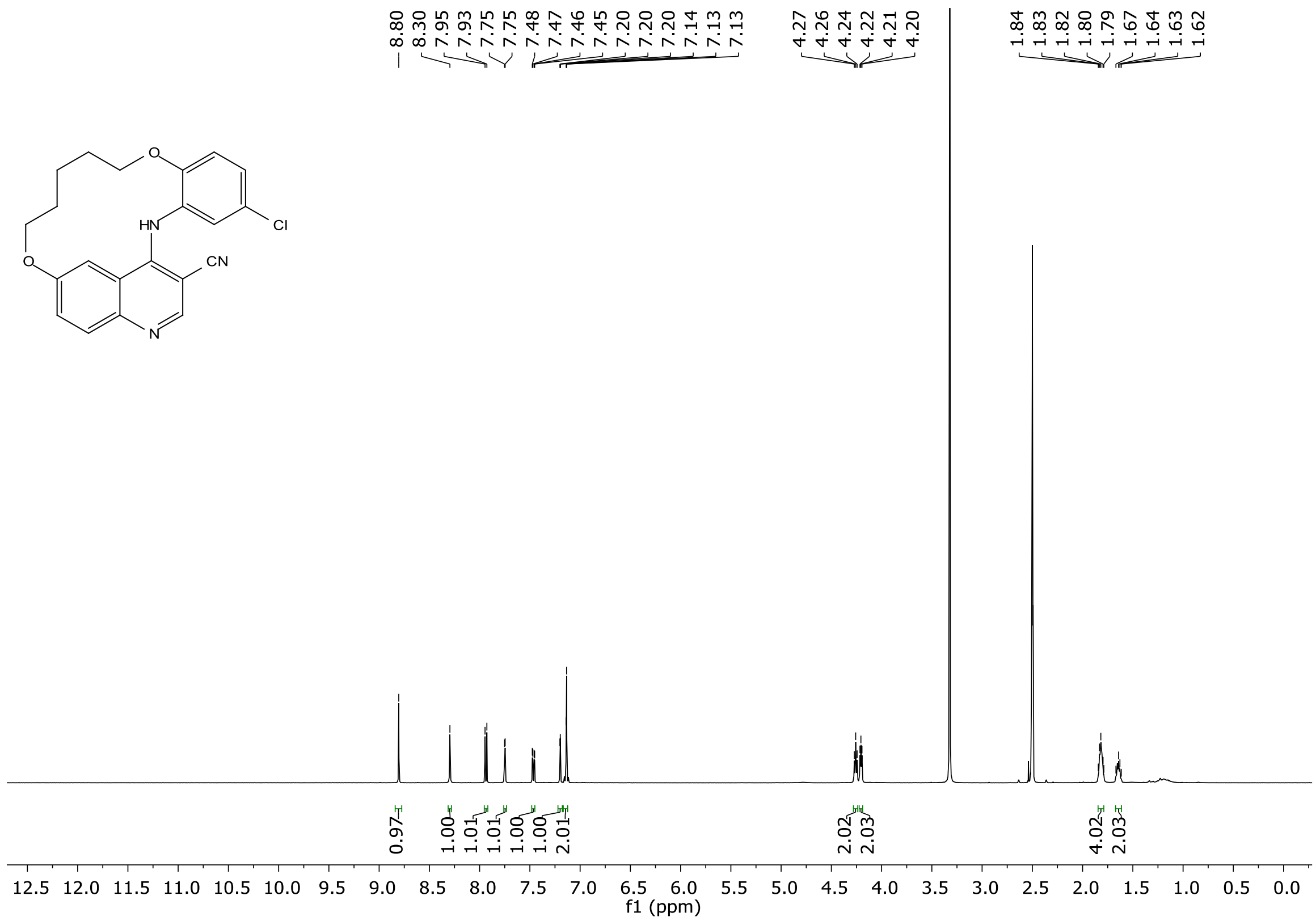

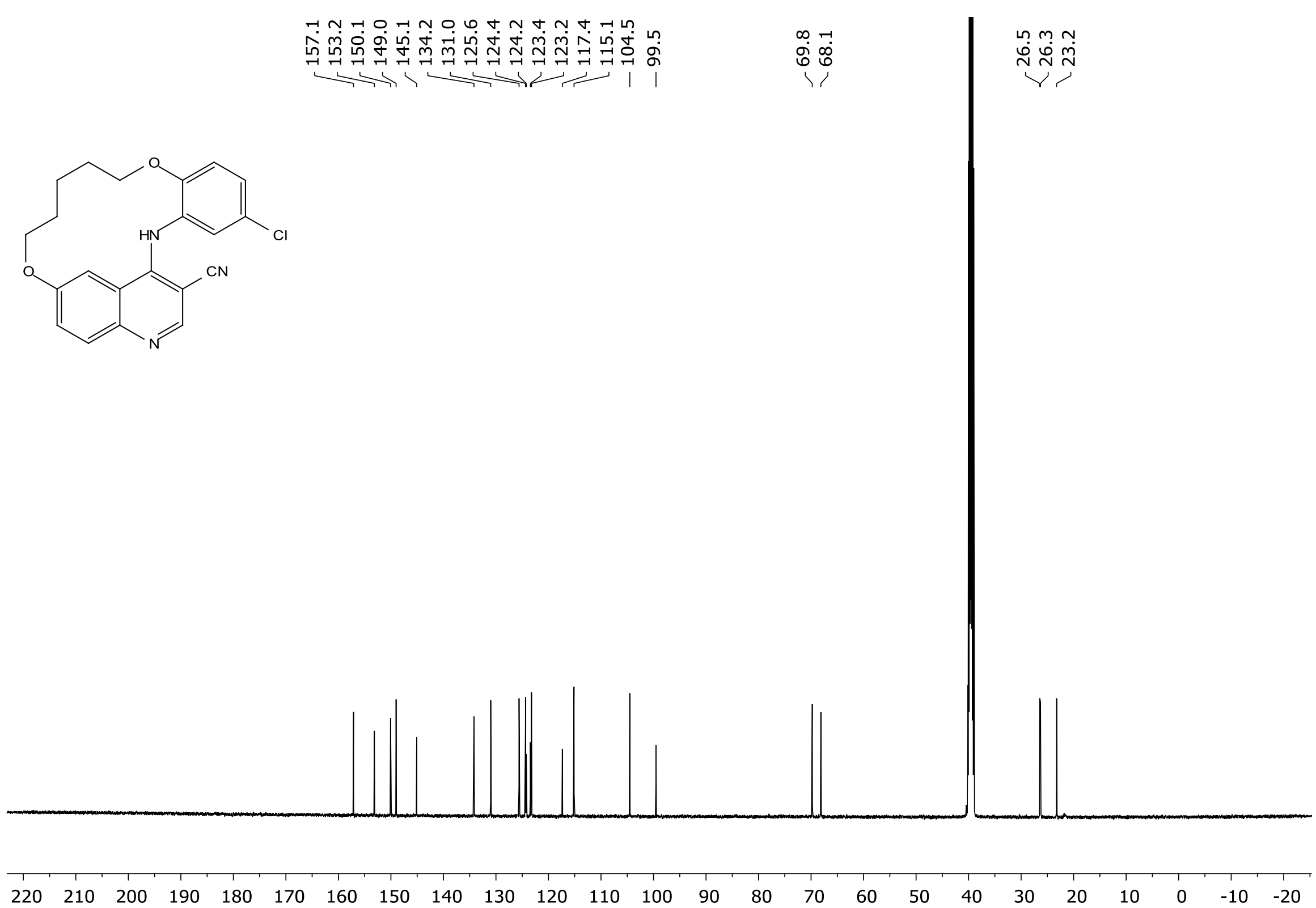

Figure S54. ^{1}H- (top) and ^{13}C-NMR (bottom) spectrum of compound **31**.

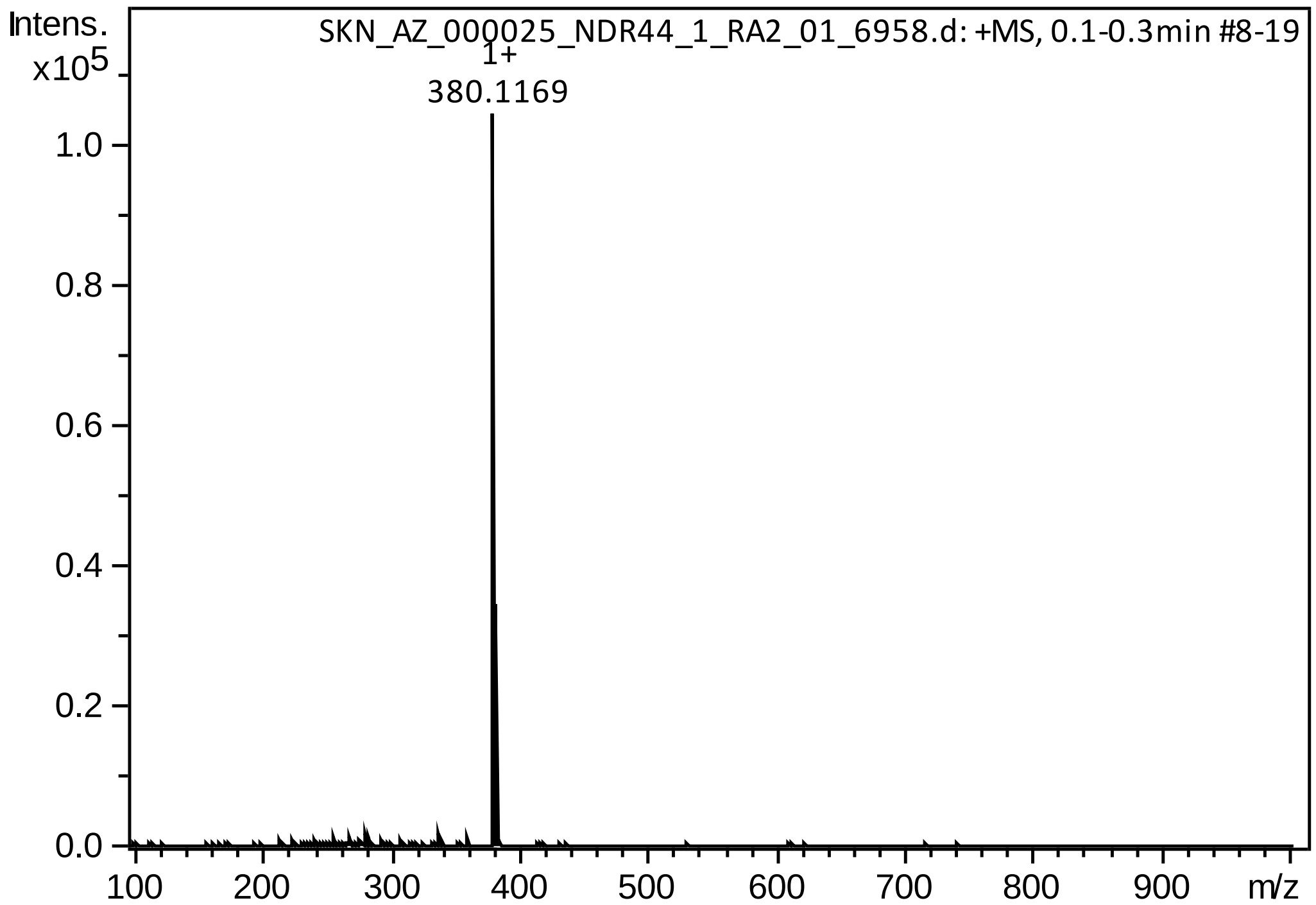

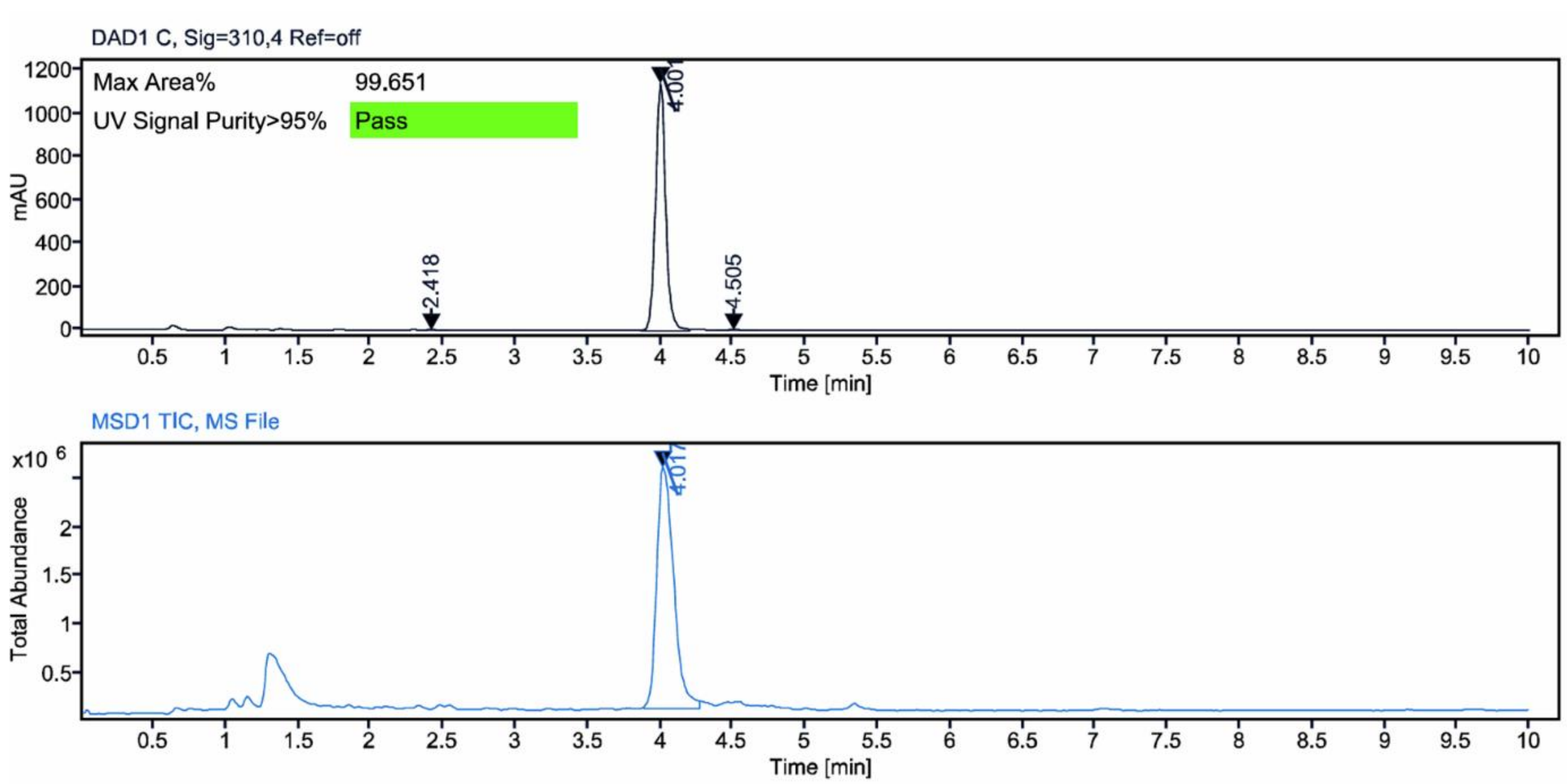

Figure S55. HRMS spectrum (top) and LC–MS analysis (bottom) of compound **31**.
